# Predicting glycosylation stereoselectivity using machine learning†

**DOI:** 10.1039/d0sc06222g

**Published:** 2020-12-26

**Authors:** Sooyeon Moon, Sourav Chatterjee, Peter H. Seeberger, Kerry Gilmore

**Affiliations:** Department of Biomolecular Systems, Max-Planck-Institute of Colloids and Interfaces Am Mühlenberg 1 14476 Potsdam Germany kerry.m.gilmore@uconn.edu; Freie Universität Berlin, Institute of Chemistry and Biochemistry Arnimallee 22 14195 Berlin Germany

## Abstract

Predicting the stereochemical outcome of chemical reactions is challenging in mechanistically ambiguous transformations. The stereoselectivity of glycosylation reactions is influenced by at least eleven factors across four chemical participants and temperature. A random forest algorithm was trained using a highly reproducible, concise dataset to accurately predict the stereoselective outcome of glycosylations. The steric and electronic contributions of all chemical reagents and solvents were quantified by quantum mechanical calculations. The trained model accurately predicts stereoselectivities for unseen nucleophiles, electrophiles, acid catalyst, and solvents across a wide temperature range (overall root mean square error 6.8%). All predictions were validated experimentally on a standardized microreactor platform. The model helped to identify novel ways to control glycosylation stereoselectivity and accurately predicts previously unknown means of stereocontrol. By quantifying the degree of influence of each variable, we begin to gain a better general understanding of the transformation, for example that environmental factors influence the stereoselectivity of glycosylations more than the coupling partners in this area of chemical space.

## Abstract 2

A random forest algorithm, trained on a concise dataset and validated experimentally, accurately predicts the stereoselectivity of a complex organic coupling varying all reaction parameters as well as previously unknown mechanistic influences.

## Graphical abstract

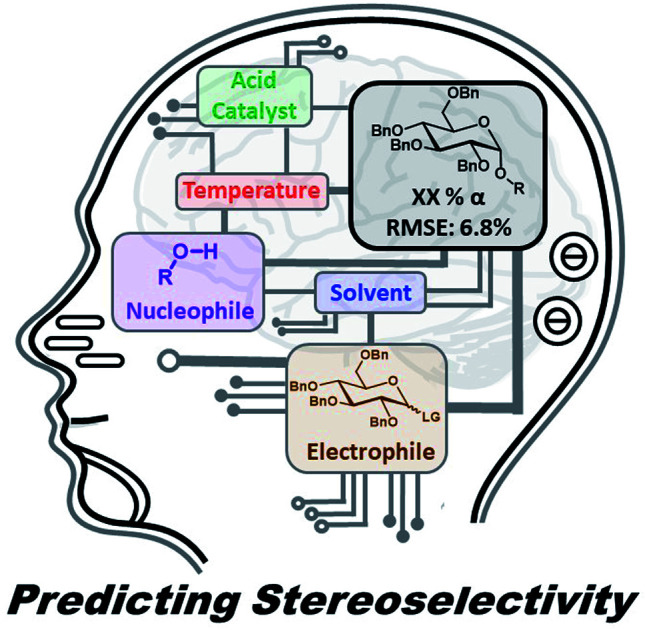

## Introduction

Predicting the outcome of an organic reaction generally requires a detailed understanding of the steric and electronic factors influencing the potential energy^1,2^ surface^3^ and intermediate(s).^4^ Quantum mechanical calculations have significantly increased our ability to identify and quantify these factors. However, the correlation of these physical properties with reaction outcome becomes exceedingly challenging with each increase in dimensionality (*e.g.*, additional reaction participants, pathways). Layering onto this the additional and often subtle nuances impacting the regio- or stereoselectivity^5^ of a reaction complicates proceedings.

Machine learning is a powerful tool for chemists^6,7^ to identify patterns in complex datasets from composite libraries or high-throughput experimentation.^8^ Chemical challenges including retrosynthesis,^9^ reaction performance^10^ and products,^11,12^ the identification of new materials and catalysts,^13–15^ as well as enantioselectivity^16,17^ have been addressed. However, a significant challenge is predictability of reactions involving S_N_1 or S_N_1-type mechanisms^18^ in the absence of chiral catalysts/ligands,^19^ due to the potentially unclear mechanistic pathways resulting from the instability of the carbocationic intermediate.^16,17,20^

**Fig. 1:**
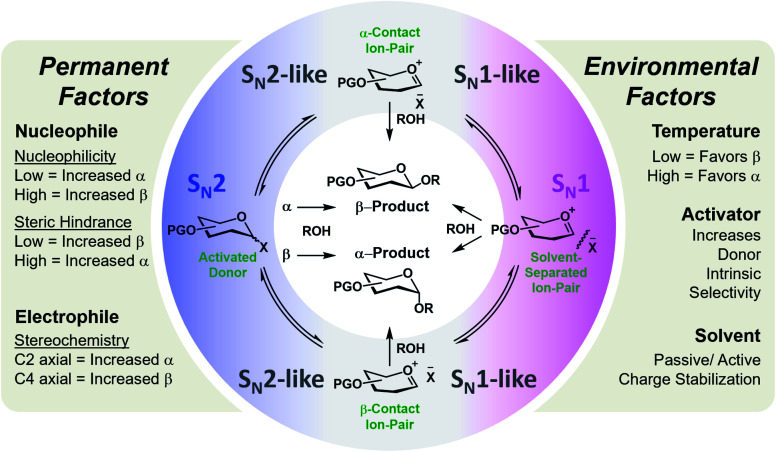
General representation of the potential mechanistic pathways of glycosylations leading to either the alpha (α) or beta (β) anomer of the formed C–O bond. The empirically-derived permanent and environmental factors and their influence on stereoselectivity are provided.^24^

Glycosylation is one of the most mechanistically complex organic transformations,^20–22^ where an electrophile (donor), upon activation with a Lewis or Brønsted–Lowry Acid, is coupled to a nucleophile (acceptor) to form a C–O bond and a stereogenic center. This reaction involves numerous potential transient cationic intermediates and conformations and can proceed *via* mechanistic pathways spanning S_N_1 to S_N_2.^23^ The stereochemical outcome is determined by numerous permanent (defined by the starting materials) or environmental factors (defined by the selected conditions/catalyst) whose degree of influence, interdepency, and relevance is poorly understood.^20,24,25^ A systematic assessment of these factors on a flow platform allowed for the isolated interrogation of these variables. The empirical study indicated general trends/influences of these factors (Fig. 1) and hypothesized their relative rankings with respect to dominance.^24^ However, a data sciences approach is required to positively identify, quantify, and apply this knowledge for the accurate prediction of stereoselectivities of new coupling partners and conditions. While transfer learning has been applied to machine learning models for the prediction of selectivities of glycosylations (reported between preprint and publication of this work), the stereoselectivity of couplings predicted were controlled by the C-2 acyl protecting group that provide a well-established, highly reproducible means of stereocontrol in these reactions.^26^

## Results and discussion

### Algorithm training and description of datasets

**Fig. 2:**
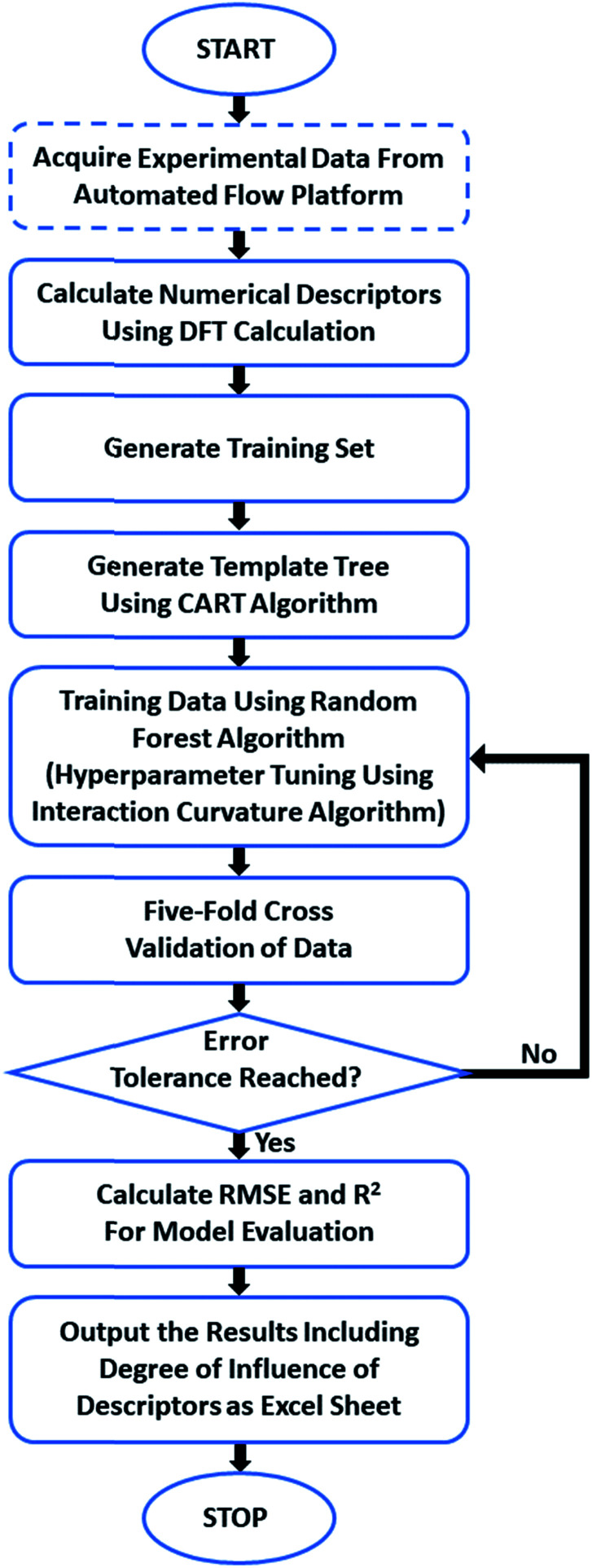
General workflow of the process from data input to prediction output.

We have trained a random forest algorithm using a dataset of glycosylation reactions with a variety of stereoselective outcomes to accurately predict the stereoselectivity of new glycosylations, varying coupling partners, acid catalyst, solvents, and temperature (pS5–S9, Table 1 of ESI†). Regression-based random forest algorithms have proven powerful in modeling chemical reaction performance.^10,27^ This algorithm generates several weak models in the form of decision trees. The nodes of each of these decision trees are generated by random shuffling of the descriptors in the training set. The final model is an “ensemble” of a combined weighted sum of decision trees, representing a collective decision of all individual trees that generate good predictions and reduces overfitting. The learning performance of the algorithm can be significantly enhanced by hyperparameter tuning (pS35 of ESI†).^28^ Due to the heterogeneous nature of the descriptors in this work (*vide infra*), each tree was generated using the CART (classification and regression tree) algorithm with pruning, which does not require preprocessing or normalization.^29^ An interaction–curvature algorithm was further utilized to reduce the selection bias of the split predictors of the standard CART algorithm (Fig. 2).

A set of numerical descriptors that accurately describe the relevant steric and electronic parameters of all reaction participants – starting materials, reagents, and solvent – is key to building an accurate, extrapolatable model to predict the subtle nuances of stereoselectivity. The concise nature of the training set (268 data points, Table S1 (pS5–S9), ESI†)^30,31^ renders manual selection of descriptors – quantifying sterics/electronics – using chemical intuition^32^ particularly important.^33^

The training dataset is a lightly modified version of the dataset presented in our previous work,^24^ removing two subsets of data (variance of the residence time and nucleophile equivalents) and adding data for β-glucose electrophile (pS6, lines 68–74 and 101–106 of Table S1, ESI†) and three additional solvents (pS9, lines 238–268 of Table S1, ESI†). Two holdout datasets were experimentally generated (HD1, HD2). The first was comprised of new electrophiles, nucleophiles, acid catalysts, and solvents. Holdout dataset 2 was comprised of examples probing the influence of electrophile leaving group stereochemistry.

### Descriptor generation

Structures of all starting compounds were optimized, and DFT calculations performed at the B3LYP 6-31G(d) or B3LYP 6-311G(d) levels of theory using SPARTAN (pS37–S49 of ESI†). The lower level of theory was utilized for optimization of the electrophile molecules due to their size, and the values obtained were acceptable compared to those obtained at the more computationally expensive 6-311G(d) level of theory. The maximum number of potential descriptors per model was set to 18 to avoid overfitting by keeping the ratio of datapoints : descriptors >10 : 1.^34,35^ The best-performing descriptors for each participant class were determined by the accuracy of the resultant trained models in predicting stereoselectivities of the relevant portions of holdout dataset 1 (*e.g.* determining the accuracy of predicting the novel electrophiles in HD1 with systematic screening of electrophile descriptors). Ten descriptors were identified that, along with temperature, allow for the assignment of quantified values to the relevant steric/electronic properties of the chemicals involved.

The identified descriptors, described below (see potential descriptors excel sheet for a list of all descriptors screened), are either classified as regressors (intra-/extrapolatable values) or categorical (binary values). While the model can be developed solely using regressor values, it exhibits marginally poorer overall accuracy for holdout dataset 1 and necessitates additional calculations (*vide infra*). The ability to interchange descriptors will facilitate the expansion of the developed model into adjacent or similar chemical subspaces as well as for multi-stage predictive algorithms, designing both reagents and environmental conditions to maximize the stereoselectivity of the desired transformation.

**Fig. 3:**
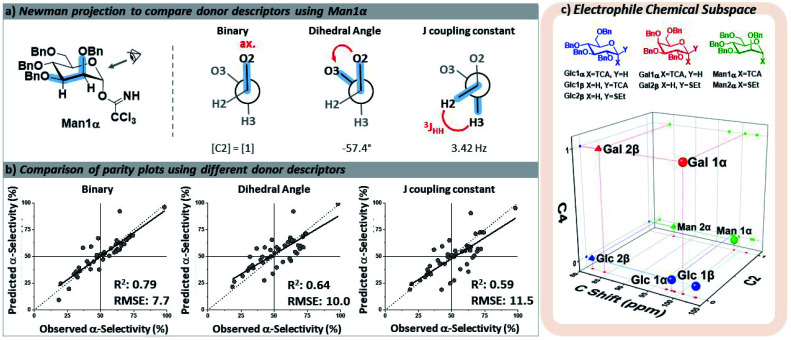
(a) Three potential means of describing the stereochemistry of the ether groups around the pyran core. (b) Parity plot of the resultant models using each set of descriptors for the electrophile (all also including the calculated ^13^C NMR shift of C1). Predictions were made of holdout dataset 1. (c) Three-dimensional map of the electrophile chemical subspace covered by the developed model, defined by the orientation of the C2 and C4 substituents on the pyran ring and the calculated ^13^C NMR shift of C1. Glc – glucose, Gal – galactose, Man – mannose, Bn – benzyl, TCA – trichloroacetimidate, SEt – ethylthio.

The key parameters needed to describe the electrophile were differences in the reactivity of the anomeric position and the orientations of the pyran ring substituents that may influence the selectivity through both conformational preferences^36^ and hyperconjugative interactions.^37,38^ The different leaving groups at the anomeric position were distinguished using the calculated ^13^C NMR chemical shift,^39^ which provided more clear distinctions between leaving groups than the ^1^H NMR shift^40^ of the anomeric proton. The relative orientations of the ether moieties around the pyran presented a challenge for descriptor selection, as our model performed well with both regressor and categorial descriptors. The accuracies of the three best performing descriptors (proton *J*-couplings around the ring, dihedral angles of the C–O bonds, and treating the relative axial/equatorial orientations of the substituents as binary) are shown in Fig. 3. The binary classification is the most accurate and represents the simplest descriptor, and the loss of additional/more nuanced information provided by regressor values – *e.g.* the influence and nature of the leaving groups – is, at present, acceptable.

**Fig. 4:**
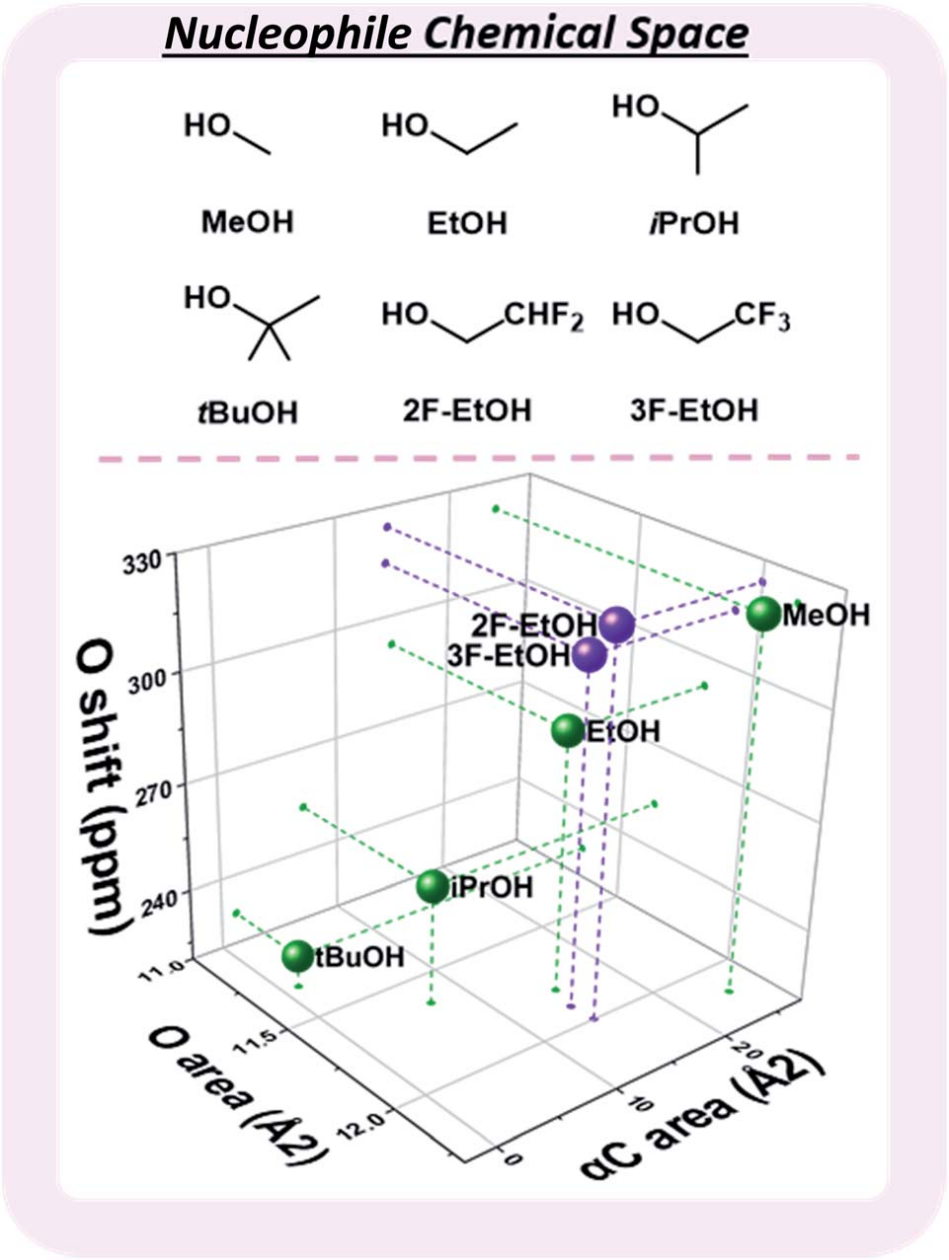
Three-dimensional map of the nucleophile chemical subspace covered by the developed model, defined by the exposed surface areas of the nucleophilic oxygen and the carbon alpha to the nucleophile, as well as the calculated ^17^O NMR shift. MeOH – methanol, EtOH – ethanol, iPrOH – isopropanol, *t*BuOH – *tert*-butanol, 2F-EtOH – 2,2-difluoroethanol, 3F-EtOH – 2,2,2-trifluoroethanol.

Observed nucleophile reactivity has been correlated with a range of parameters.^41–43^ Where available, Mayr's nucleophilicity and field inductive parameters correlate with glycosylation stereoselectivity.^44^ To ensure general applicability, the ^17^O NMR chemical shift of the oxygen nucleophile was calculated to capture the relevant hyperconjugative influences. The steric environment of the nucleophile was described by the exposed surface areas of the oxygen and α-carbon in a space-filling model (Fig. 4). While screening whether simple categorical descriptors can be utilized, specifically the whole values 0–3 to describe the substitution at the α-carbon (as opposed to the exposed surface area), we found that the regressor value proved superior (see ESI†).

**Fig. 5:**
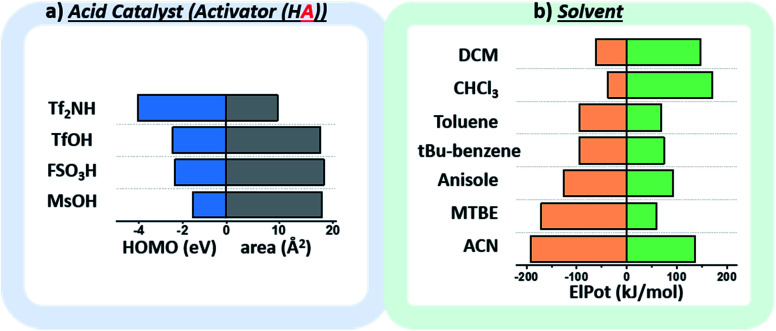
(a) Plot of the descriptors used to quantify the relevant factors of the conjugate base of the activator. Area (Å^2^) corresponds to the exposed surface area of the oxygen (O^−^) or nitrogen anion (N^−^) in a space-filling model. HOMO: highest occupied molecular orbital (eV). (b) Plot of the descriptors used to quantify the relevant factors of the solvent, the maximum (MaxElPot), and minimum (MinElPot) values of the electrostatic potential (kJ mol^−1^). Tf_2_NH – bis(trifluoromethane)sulfonamide, TfOH – trifluoromethanesulfonic acid, FSO_3_H – fluorosulfonic acid, MsOH – methanesulfonic acid, DCM – dichloromethane, CHCl_3_ – chloroform, *t*Bu-benzene – *tert*-butylbenzene, MTBE – methyl *tert*-butyl ether, ACN – acetonitrile.

The chosen environmental conditions – solvent, acid catalyst, and temperature – are even more influential on the stereoselectivity than the intrinsic properties of the nucleophile and electrophile (*vide infra*). While regressor values for similar species have been calculated previously, the identification of the descriptors for acid catalysts relevant to this transformation was critical. The conjugate base of the acid catalyst has a significant impact on glycosylation stereoselectivity,^45^ as evidenced by several studies observing an α-triflate intermediate^20,46^ – the product of the conjugate base trapping the oxycarbenium ion.^47^ Two values were identified that capture the nuanced role of this species (Fig. 5a): the HOMO energy value of the conjugate base and the exposed surface area of the oxygen or nitrogen anion in a space-filling model.

While the influence of the solvent in glycosylations^48,49^ has been categorized by polarity and donicity (coordinating ability) values,^20^ donicities are experimentally derived values and only available for select solvents. The calculated minimum and maximum electrostatic potentials describe the ability of the solvent to stabilize and interact with charged intermediates (Fig. 5b). These descriptors perform well, such that even previously unreported means of solvent-control over stereoselectivity are accurately predicted (*vide infra*).

### Model training and algorithm comparison

The tuned random forest algorithm was trained using these descriptors on the training dataset^24^ containing systematic combinations of seven electrophiles, six nucleophiles, four acid catalysts, and seven solvents over a solvent-dependent temperature range of −50 to +100 °C (pS5–S9, Table S1, ESI†). For comparison, three additional models were trained using Gaussian process regression (GPR), support vector machine (SVM), and regression tree (RT) algorithms. While for some specific predictions different algorithms would have lower RMSEs, random forest (RF) proved superior. The average RMSE of the four models were: RF – 5.9%, RT – 11%, GPR – 7.9%, SVM – 10%. In general terms, RT tended to overestimate the preference for beta-product formation at low temperatures, GPR captured the trend of stereoselectivity change with respect to temperature but lacked precision, and SVM often predicted no influence of temperature yielding a racemic mixture of products (see pages S14–S28 of the ESI†).

**Fig. 6:**
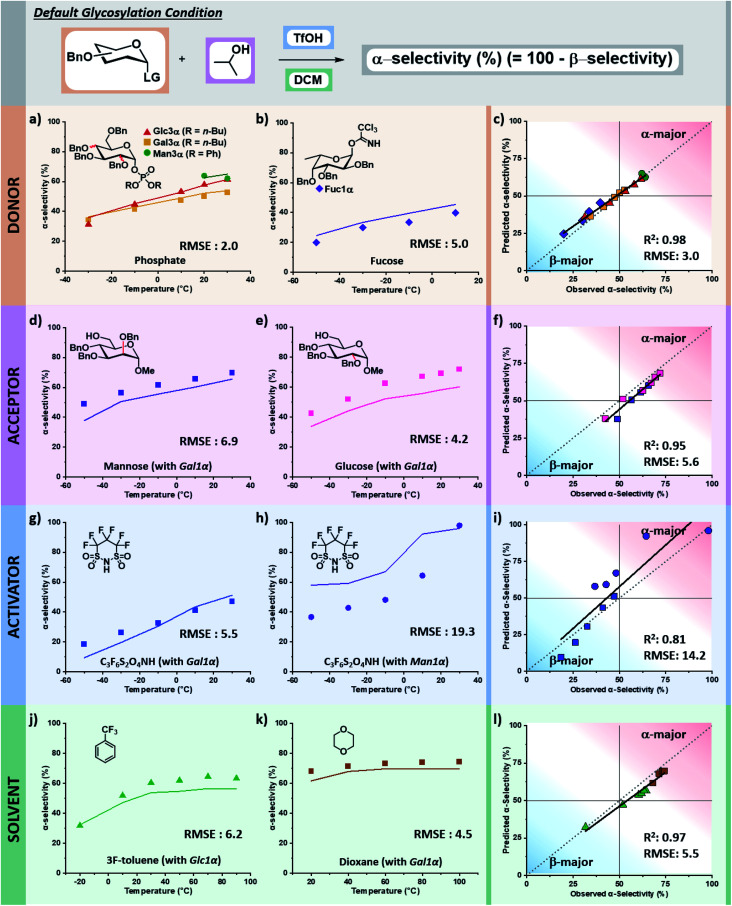
Prediction of stereoselectivity for glycosylations using different anomeric leaving groups, electrophiles, nucleophiles, activators, and solvents. Descriptors used were: electrophile (C1: ^13^C NMR shift, C2: stereochemistry axial = 1 or equatorial = 0, C4: stereochemistry axial = 1 or equatorial = 0), nucleophile (O: ^17^O NMR shift, O: exposed surface area, αC: exposed surface area), acid catalyst (A^−^: HOMO energy, A^−^: exposed surface area), solvent (minimum electrostatic potential, maximum electrostatic potential), and temperature (−50 to 100 °C). (a) Prediction of stereoselectivity for glycosylations involving a glycosyl phosphate leaving group. Bu – butyl, Ph – phenyl, RMSE – root mean square error. TMSOTf was used as acid catalyst, which has the same descriptors as TfOH. (b) Prediction of stereoselectivity using a fucose (Fuc) electrophile with iPrOH in DCM. (c) Parity plot of electrophile (electrophile) predictions. (d and e) Prediction of mannose and glucose nucleophile, respectively, with galactose α-imidate electrophile in DCM. (f) Parity plot of nucleophile (nucleophile) predictions. (g) Prediction of 4,4,5,5,6,6-hexafluoro-1,3,2-dithiazinane 1,1,3,3-tetraoxide (C_3_F_6_S_2_O_4_NH) activator with galactose electrophile and *t*BuOH nucleophile in DCM. (h) Prediction of C_3_F_6_S_2_O_4_NH with mannose electrophile and iPrOH in DCM. (i) Parity plot of activator (acid catalyst) predictions. (j) Prediction of α,α,α-trifluorotoluene (3F-toluene) solvent with glucose α-imidate electrophile and iPrOH. (k) Prediction of 1,4-dioxane solvent with galactose α-imidate electrophile and iPrOH. (l) Parity plot of solvent predictions. Figure code: fucose (◆); glucose (▲); galactose (■); mannose (●); experimental (data points); predicted (solid colored line).

The trained RF model was then used to predict the stereoselectivities of the entirety of holdout dataset 1, containing unseen variants of each of the four chemical species in the reaction over the accessible temperature ranges (defined by the solvent and reactor). Holdout dataset 1 (see holdout dataset 1 excel sheet of ESI†) was generated using the same reproducible microreactor platform^24^ as the training dataset. The results of these predictions, as compared to the experimentally observed selectivities, are presented as the percentage of alpha product formed *versus* temperature. The corresponding parity plots for each are also provided (Fig. 6).

### Validation of descriptors and prediction accuracy of holdout dataset 1

The selectivity of electrophiles bearing phosphate leaving groups is accurately predicted to be similar^24^ to those of glycosyl imidates and thioethers for glucose, galactose, and mannose electrophiles, with a combined root mean square error (RMSE) of 2.0 (Fig. 6a). The model can be applied to other pyran cores, such as l-fucose.^50^ The predicted stereoselectivity of the fucose α-glycosyl imidate electrophile with isopropanol matches well with the experimental data (RMSE: 5.0), favoring the β-anomer at low temperatures and exhibiting a decrease in stereoselectivity with an increase in temperature (Fig. 6b).

While the training dataset contains only simple alkyl alcohols as nucleophiles, the model accurately predicts the stereoselectivities of disaccharide formation. The predicted values for the coupling of α-galactose imidate with both glucose and mannose C6 alcohols matches well with the experimental data, albeit predicting a less α-selective process than observed (RMSE: 6.9 and 4.2, Fig. 6d and e, respectively).

The model predicts more α-selective processes than experimentally observed in glycosylations using superacid 4,4,5,5,6,6-hexafluoro-1,3,2-dithiazinane-1,1,3,3-tetraoxide (C_3_F_6_S_2_O_4_NH) as acid catalyst. This deviation is seen at lower temperatures with galactose, however, the trend is correct and has a low RMSE (5.5, Fig. 6g). The weakest correlation of our model is observed for the C_3_F_6_S_2_O_4_NH-activated mannose coupling with *tert*-butanol in DCM (RMSE: 19.3). Here, a stereoselective plateau is predicted at low temperatures with α-selectivity around 60% – as was observed experimentally for other activators with mannose.^24^ However, experimentally the β-mannosylation product is mainly formed at low temperatures (−50 °C, 63% β-product). This finding is highly unexpected as β-mannosylation is challenging, generally requiring locked electrophile configurations.^21^ With C_3_F_6_S_2_O_4_NH, the perbenzylated electrophile ranges from a 63% β-selectivity at −50 °C to 98% α-selectivity at 30 °C (Fig. 6h).

Finally, the stereoselectivities of glucose and galactose α-imidate electrophiles with isopropanol were predicted for two new solvents (Fig. 6j and k). The strong influence of solvent^48,51^ on the stereoselectivity of glycosylations is nicely captured by the descriptors chosen, and the model is accurate across a wide temperature range for both α,α,α-trifluorotoluene (RMSE: 6.2) and 1,4-dioxane (RMSE: 4.5).

### Model validation of unreported influences on stereoselectivity (holdout dataset 2)

**Fig. 7:**
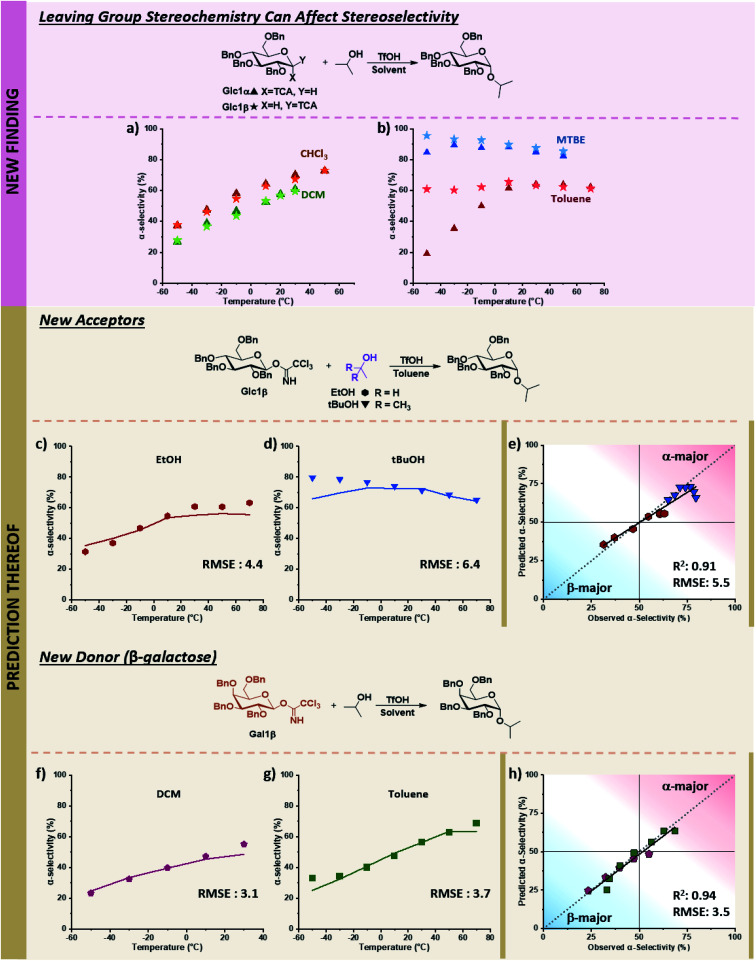
Prediction of novel mechanistic controls of glycosylation reactions using holdout dataset 2, with experimental data shown as points and predicted data shown as lines. The relevant experimental data for the α-electrophiles can be found in Table S1 of the ESI.† (a) Experimental results of coupling α/β-glucose electrophiles with iPrOH (Glc1α and Glc1β) in DCM and CHCl_3_. (b) Experimental results of coupling α/β-glucose electrophiles with iPrOH (Glc1α and Glc1β) in toluene, and MTBE. (c) Prediction and experimental results of β-glucose electrophile (Glc1β) with EtOH in toluene. (d) Prediction and experimental results of β-glucose electrophile (Glc1β) with tBuOH in toluene. (e) Parity plot of EtOH and *t*-BuOH nucleophile predictions with the β-glucose electrophile. (f and g) Prediction and experimental results of β-galactose electrophile (Gal1β) with iPrOH in DCM and toluene, respectively. (h) Parity plot for DCM and toluene solvent predictions of the β-galactose electrophile with iPrOH. Figure code: Glc1α (▲); Glc1β (★); EtOH ( <svg xmlns="http://www.w3.org/2000/svg" version="1.0" width="16.000000pt" height="16.000000pt" viewBox="0 0 16.000000 16.000000" preserveAspectRatio="xMidYMid meet"><metadata> Created by potrace 1.16, written by Peter Selinger 2001-2019 </metadata><g transform="translate(1.000000,15.000000) scale(0.014583,-0.014583)" fill="currentColor" stroke="none"><path d="M400 920 l0 -40 -40 0 -40 0 0 -40 0 -40 -40 0 -40 0 0 -40 0 -40 -40 0 -40 0 0 -40 0 -40 -40 0 -40 0 0 -200 0 -200 40 0 40 0 0 -40 0 -40 80 0 80 0 0 -40 0 -40 40 0 40 0 0 -40 0 -40 80 0 80 0 0 40 0 40 40 0 40 0 0 40 0 40 80 0 80 0 0 40 0 40 40 0 40 0 0 200 0 200 -40 0 -40 0 0 40 0 40 -40 0 -40 0 0 40 0 40 -40 0 -40 0 0 40 0 40 -40 0 -40 0 0 40 0 40 -80 0 -80 0 0 -40z"/></g></svg> ); *t*BuOH (▼); DCM ( <svg xmlns="http://www.w3.org/2000/svg" version="1.0" width="18.545455pt" height="16.000000pt" viewBox="0 0 18.545455 16.000000" preserveAspectRatio="xMidYMid meet"><metadata> Created by potrace 1.16, written by Peter Selinger 2001-2019 </metadata><g transform="translate(1.000000,15.000000) scale(0.015909,-0.015909)" fill="currentColor" stroke="none"><path d="M400 760 l0 -40 -40 0 -40 0 0 -40 0 -40 -80 0 -80 0 0 -40 0 -40 -40 0 -40 0 0 -40 0 -40 40 0 40 0 0 -40 0 -40 -40 0 -40 0 0 -40 0 -40 40 0 40 0 0 -80 0 -80 40 0 40 0 0 -80 0 -80 280 0 280 0 0 40 0 40 40 0 40 0 0 160 0 160 40 0 40 0 0 80 0 80 -40 0 -40 0 0 40 0 40 -40 0 -40 0 0 40 0 40 -80 0 -80 0 0 40 0 40 -120 0 -120 0 0 -40z"/></g></svg> ); toluene (■); experimental values (data points) and predicted values (solid colored lines).

While the descriptors were chosen based on the current understanding of glycosylations, we wondered whether the model could also navigate newly discovered mechanistic peculiarities that influence stereoselectivity. One factor that is generally not considered significant while performing glycosylations is the orientation of the anomeric leaving group.^52,53^ No influence of the α/β-orientation of the leaving group in dichloromethane was reported (Fig. 7a),^24^ and divergences in stereoselectivity based on this factor have sparingly been observed in the literature, *e.g.*, when phenylsilicon trifluoride (PhSiF_3_) is used as a catalyst.^54^

The ability to use solvent to turn on and off the influence of leaving group orientation on glycosylation stereoselectivity has, to the best of our knowledge, not previously been reported. While essentially identical behavior is observed in DCM and chloroform, a slight divergence in MTBE at low temperatures is observed, with an 11% difference at −50 °C where the β-electrophile reaches 96% α-selectivity. This variable becomes important in toluene. Glucose β-imidate electrophile yields almost unchanged stereoselectivity (∼60% alpha) over a 120 °C range! The orientation of the leaving group of the electrophile influences the stereoselectivity by more than 40% at −50 °C (Fig. 7b).

With this limited data in our training dataset (Fig. 7a and b), we tested the ability of our model to predict the influence of other factors on this to-date unreported phenomenon in holdout dataset 2, whose experimental values were obtained on the same microreactor platform as TD and HD1 (see holdout dataset 2 excel sheet of ESI†). The stereoselectivity of glucose α-imidate with ethanol as nucleophile ranges from 10–54% α-product in toluene. The model predicts that the β-electrophile will behave differently, with a much less selective coupling overall (37–56% α-product) and a 27% difference in selectivity at low temperature compared to the α-electrophile. This prediction matches well with the experimental results, with an RMSE of 4.4 over the 120 °C range (Fig. 7c). The model predicts a less α-selective reaction at low temperatures than observed with *t*-BuOH as nucleophile (similar to what is observed using the α-electrophile, pS6, lines 82–88 of Table S1, ESI†), though at higher temperatures, the prediction matches well with experimental values (RMSE: 6.4, Fig. 7d).

Lastly, we sought to explore whether this additional mechanistic complexity exists for other electrophiles (Fig. 7f and g). In DCM, the coupling of α-galactose with isopropanol moderately favors the formation of the β-product (19–51% α-product from −50 to 30 °C, (pS7, lines 119–124, of ESI†)). The model predicts that the β-galactose electrophiles will give similar α-selectivity in DCM over the 80 °C temperature range (24–49% α-product), matching experimental results (RMSE 3.1, Fig. 7f). In toluene, the α-galactose electrophile exhibits a wide range of selectivities with isopropanol, from 10–69% α-product across the 130 °C range (pS7, lines 142–148, of ESI†). The model predicts a slight divergence (15%) in stereoselectivity at low temperatures when the β-galactose electrophile is used (25–64% α-product, −50 to 70 °C), though not as large as what is observed with β-glucose. This prediction again aligns with experimental results (RMSE: 3.7, Fig. 7g). Overall, the model correctly predicts the previously unknown ability to turn on and off the influence of the electrophile leaving group's orientation using solvents under otherwise identical conditions. We hypothesize the decrease of stereoselectivities for β-electrophiles when using toluene may result from an increase in the S_N_1-type pathways. The π-system of the solvent can more easily induce solvolysis of the more planar equatorial leaving group from both faces (as compared to the axial orientation), leading to an accessible oxonium ion instead of an α-triflate intermediate. Additional detailed mechanistic studies are required to discern the degree and nature of mechanistic control.

### Overall influences of permanent and environmental factors on stereoselectivity

**Fig. 8:**
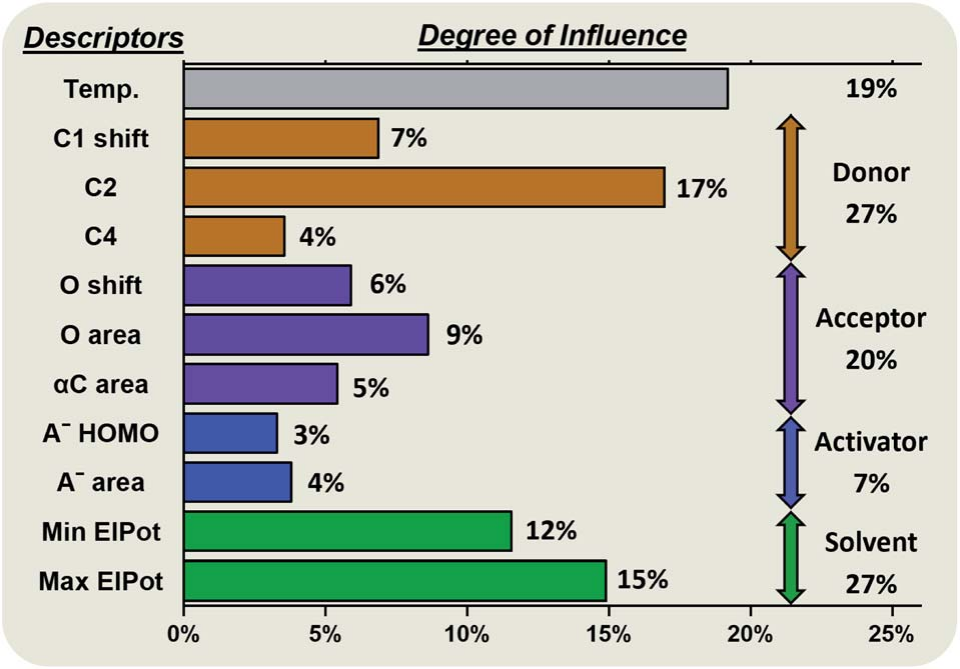
Degree of influence of the eleven factors (defined and described above) influencing the stereoselectivity of glycosylations, rounded to the nearest whole number.

Random forest algorithms can quantify the influence of the variables within the model. Thus, values can be assigned to the identified factors influencing the stereoselectivity of a reaction (Fig. 8), allowing for some cautious generalizations to be made. In the chemical subspaces covered by our model, 47% of the influence over a glycosylation's stereoselectivity is determined by the inherent properties of the coupling partners. The electrophile (27%) is more impactful than the nucleophile (20%). Upon selection of the coupling partners, more than half of the stereoselectivity observed is controlled by the environmental conditions chosen. The most important environmental factors are the solvent (27%) and the reaction temperature (19%).

## Conclusion

A concise training dataset generated on a continuous flow platform was utilized to train a random forest algorithm to predict the stereoselectivity of glycosylations as an example for complex, mechanistically fluid transformations. Calculated descriptors were screened and assigned to quantify the individual influencing factors of the coupling partners, active species, and solvent. The predictions of glycosylation stereoselectivities were made of two holdout datasets – testing nucleophiles, electrophiles, catalyst, solvents, and temperature – containing data obtained experimentally on a microreactor platform. The model is highly accurate (overall RMSE: 6.8) in the chemical subspaces explored. Further, the model accurately predicts a previously unknown means of controlling glycosylation stereoselectivity. The approach will be applicable to better understand the stereoselectivity of other transformations based on reactions of nucleophiles and electrophiles.

## Conflicts of interest

None of the authors declare any competing interests.

## Supplementary Material

SC-012-D0SC06222G-s001

SC-012-D0SC06222G-s002

SC-012-D0SC06222G-s003

SC-012-D0SC06222G-s004

SC-012-D0SC06222G-s005

SC-012-D0SC06222G-s006
