# Supplementary material for: Predicting glycosylation stereoselectivity using machine learning
Source: Chem Sci. 2020 Dec 26;12(8):2931–9. doi: 10.1039/d0sc06222g (PMC8179398; doi:10.1039/d0sc06222g)
Supplement: SC-012-D0SC06222G-s001 [file SC-012-D0SC06222G-s001.pdf]

Electronic Supplementary Material (ESI) for Chemical Science.
This journal is © The Royal Society of Chemistry 2020

SUPPLEMENTARY INFORMATION

Predicting Glycosylation Stereoselectivity Using Machine Learning

Sooyeon Moon,[1,2]† Sourav Chatterjee,[1]† Peter H. Seeberger,[1,2] Kerry Gilmore[1*]

[1] *Department of Biomolecular Systems, Max-Planck-Institute of Colloids and Interfaces, Am Mühlenberg 1, 14476 Potsdam, Germany*
[2] *Freie Universität Berlin, Institute of Chemistry and Biochemistry, Arnimallee 22, 14195 Berlin, Germany*

Corresponding Author
kerry.gilmore@mpikg.mpg.de; Current Address: Department of Chemistry, University of Connecticut, 55 N. Eagleville rd, Storrs, CT, USA
† These authors contributed equally to this work.

Contents

Training Set 3
Default Glycosylation Condition 4
New Electrophile, Nucleophile, Activator and Solvent 10
Quantification of descriptors 10
New Test Set 14
Prediction of phosphate leaving group 14
Prediction of Electrophile- Fucose 15
Prediction of Nucleophile- Mannose 17
Prediction of Nucleophile- Glucose 18
Prediction of Activator- 4,4,5,5,6,6-hexafluoro-1,3,2-dithiazinane 1,1,3,3-tetraoxide with Galactose Electrophile 20
Prediction of Solvent- α,α,α-trifluorotoluene 22
Prediction of Solvent-1,4-dioxane 24
Prediction of leaving group stereochemistry 25
β-glucose electrophile with ethanol in toluene 25
β-glucose electrophile with *tert*-butanol in toluene 26
β-galactose electrophile with isopropanol in DCM 27
β-galactose electrophile with isopropanol in toluene 28
General experimental details for preparing building blocks 30
Procedure for drying solvents 30
Calculation of descriptor 30
Analysis Section 30
Machine Learning software development 35
1. Data input and preconditioning section: 35
2. Model Input Section: 36
3. Machine Learning and Data Processing section: 36
4. Prediction Section: 36
5. Data output Section: 37
Software for screening of different Machine Learning Algorithms as a benchmark study 37
XYZ Coordinates 37
References: 50
NMR spectra 51

Training Set

The following compounds were included in the training set and are separated into the four categories (electrophile (donor), nucleophile (acceptor), activator (acid catalyst), and solvent).

Electrophile

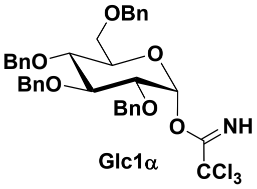
Glc1α

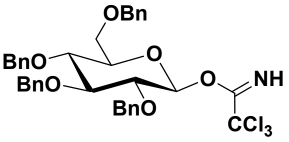
Glc1β

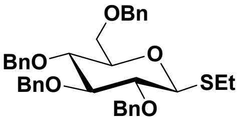
Glc2β

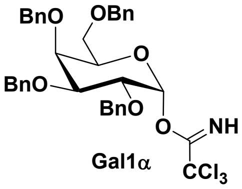
Gal1α

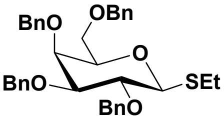
Gal2β

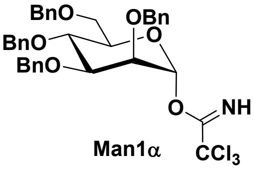
Man1α

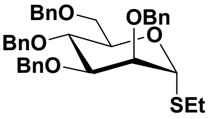
Man2α

Nucleophile

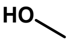
MeOH

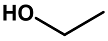
EtOH

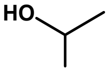
*i*PrOH

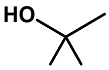
*t*BuOH

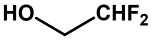
2F-EtOH

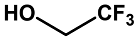
3F-EtOH

Activator

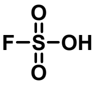
FSO_3H

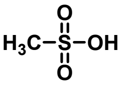
MsOH

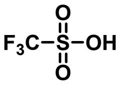
TfOH

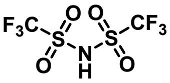
Tf_2NH

Solvent

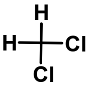
DCM

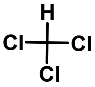
Chloroform

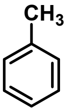
toluene

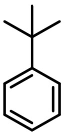
tert-butylbenzene

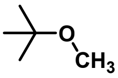
MTBE

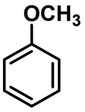
anisole

$N{\equiv}C{-}CH_3$
ACN

Figure S1: The compounds included in the training set.

Default Glycosylation Condition

Glycosylations were performed in an automated microreactor flow platform.[1] Compounds listed in the training set above were combined using the following stoichiometries (Scheme S1). Yields and selectivities were determined by on-line HPLC following calibrations on pure products. The complete training dataset is provided in Table S1 and as a supplementary excel file.

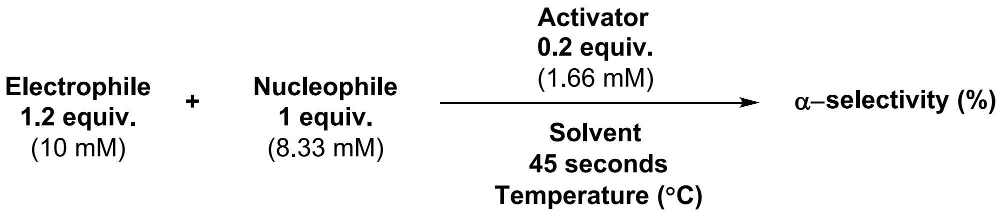

Scheme S1: Reaction condition of automated microreactor platform.[1]

Table S1: Training data collected from the automated flow platform.

Entry	Temp. (°C)	Donor	Acceptor	Activator	Solvent	Yield (%)	α ratio (%)	β ratio (%)	Product
1	-50	Glc1α	MeOH	TfOH	DCM	92.4	29.6	70.4	1α, 1β
2	-30	Glc1α	MeOH	TfOH	DCM	95.7	32.5	67.5	1α, 1β
3	-10	Glc1α	MeOH	TfOH	DCM	98.7	38.7	61.3	1α, 1β
4	10	Glc1α	MeOH	TfOH	DCM	98.1	46.5	53.5	1α, 1β
5	20	Glc1α	MeOH	TfOH	DCM	94.1	48.7	51.3	1α, 1β
6	30	Glc1α	MeOH	TfOH	DCM	93.7	52.6	47.4	1α, 1β
7	-50	Glc1α	EtOH	TfOH	DCM	83.2	15.5	84.5	2α, 2β
8	-30	Glc1α	EtOH	TfOH	DCM	83.5	29.4	70.6	2α, 2β
9	-10	Glc1α	EtOH	TfOH	DCM	100.0	36.0	64.0	2α, 2β
10	10	Glc1α	EtOH	TfOH	DCM	87.0	46.2	53.8	2α, 2β
11	20	Glc1α	EtOH	TfOH	DCM	90.7	50.2	49.8	2α, 2β
12	30	Glc1α	EtOH	TfOH	DCM	83.5	55.5	44.5	2α, 2β
13	-50	Glc1α	iPrOH	TfOH	DCM	98.3	26.9	73.1	3α, 3β
14	-30	Glc1α	iPrOH	TfOH	DCM	88.5	38.8	61.2	3α, 3β
15	-10	Glc1α	iPrOH	TfOH	DCM	90.1	46.7	53.3	3α, 3β
16	10	Glc1α	iPrOH	TfOH	DCM	99.2	52.6	47.4	3α, 3β
17	20	Glc1α	iPrOH	TfOH	DCM	84.0	57.7	42.3	3α, 3β
18	30	Glc1α	iPrOH	TfOH	DCM	82.1	61.0	39.0	3α, 3β
19	-50	Glc1β	iPrOH	TfOH	DCM	88.6	27.7	72.3	3α, 3β
20	-45	Glc1β	iPrOH	TfOH	DCM	79.4	30.6	69.4	3α, 3β
21	-40	Glc1β	iPrOH	TfOH	DCM	70.6	32.7	67.3	3α, 3β
22	-35	Glc1β	iPrOH	TfOH	DCM	65.0	34.4	65.6	3α, 3β
23	-30	Glc1β	iPrOH	TfOH	DCM	77.9	36.7	63.3	3α, 3β
24	-25	Glc1β	iPrOH	TfOH	DCM	72.4	37.4	62.6	3α, 3β
25	-20	Glc1β	iPrOH	TfOH	DCM	71.2	38.6	61.4	3α, 3β
26	-15	Glc1β	iPrOH	TfOH	DCM	94.7	41.9	58.1	3α, 3β
27	-10	Glc1β	iPrOH	TfOH	DCM	94.7	43.7	56.3	3α, 3β
28	-5	Glc1β	iPrOH	TfOH	DCM	95.9	45.6	54.4	3α, 3β
29	5	Glc1β	iPrOH	TfOH	DCM	93.5	50.5	49.5	3α, 3β
30	10	Glc1β	iPrOH	TfOH	DCM	94.8	53.2	46.8	3α, 3β
31	15	Glc1β	iPrOH	TfOH	DCM	87.1	55.0	45.0	3α, 3β
32	20	Glc1β	iPrOH	TfOH	DCM	89.4	56.8	43.2	3α, 3β
33	25	Glc1β	iPrOH	TfOH	DCM	88.7	58.3	41.7	3α, 3β
34	30	Glc1β	iPrOH	TfOH	DCM	79.1	59.6	40.4	3α, 3β
35	-50	Glc1α	tBuOH	TfOH	DCM	77.9	38.1	61.9	4α, 4β
36	-30	Glc1α	tBuOH	TfOH	DCM	87.7	44.8	55.2	4α, 4β
37	-10	Glc1α	tBuOH	TfOH	DCM	88.3	54.5	45.5	4α, 4β
38	10	Glc1α	tBuOH	TfOH	DCM	88.3	62.8	37.2	4α, 4β
39	20	Glc1α	tBuOH	TfOH	DCM	91.5	66.8	33.2	4α, 4β
40	30	Glc1α	tBuOH	TfOH	DCM	80.7	71.0	29.0	4α, 4β
41	-10	Glc2β	iPrOH	TfOH	DCM	44.6	38.0	62.0	3α, 3β
42	10	Glc2β	iPrOH	TfOH	DCM	88.1	54.5	45.5	3α, 3β
43	20	Glc2β	iPrOH	TfOH	DCM	89.5	57.5	42.5	3α, 3β
44	30	Glc2β	iPrOH	TfOH	DCM	90.4	63.8	36.2	3α, 3β
45	-50	Glc1α	tBuOH	Tf2NH	DCM	67.2	6.1	93.9	4α, 4β
46	-30	Glc1α	tBuOH	Tf2NH	DCM	62.1	16.8	83.2	4α, 4β
47	-10	Glc1α	tBuOH	Tf2NH	DCM	55.2	35.2	64.8	4α, 4β
48	20	Glc1α	tBuOH	Tf2NH	DCM	59.5	47.9	52.1	4α, 4β
49	-50	Glc1α	iPrOH	Tf2NH	DCM	95.5	8.7	91.3	3α, 3β
50	-30	Glc1α	iPrOH	Tf2NH	DCM	89.3	16.4	83.6	3α, 3β
51	-10	Glc1α	iPrOH	Tf2NH	DCM	82.2	26.8	73.2	3α, 3β
52	10	Glc1α	iPrOH	Tf2NH	DCM	79.2	42.1	57.9	3α, 3β
53	20	Glc1α	iPrOH	Tf2NH	DCM	76.7	47.5	52.5	3α, 3β
54	30	Glc1α	iPrOH	Tf2NH	DCM	76.0	55.0	45.0	3α, 3β

55	-50	Glc1α	EtOH	Tf2NH	DCM	64.2	5.8	94.2	2α, 2β
56	-30	Glc1α	EtOH	Tf2NH	DCM	64.1	10.4	89.6	2α, 2β
57	-10	Glc1α	EtOH	Tf2NH	DCM	62.6	19.6	80.4	2α, 2β
58	10	Glc1α	EtOH	Tf2NH	DCM	65.5	33.6	66.4	2α, 2β
59	20	Glc1α	EtOH	Tf2NH	DCM	57.4	39.0	61.0	2α, 2β
60	30	Glc1α	EtOH	Tf2NH	DCM	53.8	45.0	55.0	2α, 2β
61	-50	Glc1α	iPrOH	TfOH	Toluene	65.7	19.1	80.9	3α, 3β
62	-30	Glc1α	iPrOH	TfOH	Toluene	84.0	35.5	64.5	3α, 3β
63	-10	Glc1α	iPrOH	TfOH	Toluene	86.2	49.9	50.1	3α, 3β
64	10	Glc1α	iPrOH	TfOH	Toluene	85.0	61.6	38.4	3α, 3β
65	30	Glc1α	iPrOH	TfOH	Toluene	84.5	64.1	35.9	3α, 3β
66	50	Glc1α	iPrOH	TfOH	Toluene	81.3	63.9	36.1	3α, 3β
67	70	Glc1α	iPrOH	TfOH	Toluene	70.2	62.3	37.7	3α, 3β
68	-50	Glc1β	iPrOH	TfOH	Toluene	84.8	60.8	39.2	3α, 3β
69	-30	Glc1β	iPrOH	TfOH	Toluene	85.4	60.3	39.7	3α, 3β
70	-10	Glc1β	iPrOH	TfOH	Toluene	90.9	62.2	39.8	3α, 3β
71	10	Glc1β	iPrOH	TfOH	Toluene	86.1	65.6	34.4	3α, 3β
72	30	Glc1β	iPrOH	TfOH	Toluene	85.3	63.3	36.7	3α, 3β
73	50	Glc1β	iPrOH	TfOH	Toluene	68.6	62.2	37.8	3α, 3β
74	70	Glc1β	iPrOH	TfOH	Toluene	56.9	61.2	38.8	3α, 3β
75	-50	Glc1α	EtOH	TfOH	Toluene	90.4	9.9	90.1	2α, 2β
76	-30	Glc1α	EtOH	TfOH	Toluene	91.5	20.0	80.0	2α, 2β
77	-10	Glc1α	EtOH	TfOH	Toluene	89.7	32.2	67.8	2α, 2β
78	10	Glc1α	EtOH	TfOH	Toluene	85.8	42.5	57.5	2α, 2β
79	30	Glc1α	EtOH	TfOH	Toluene	82.2	49.6	50.4	2α, 2β
80	50	Glc1α	EtOH	TfOH	Toluene	75.6	53.3	46.7	2α, 2β
81	70	Glc1α	EtOH	TfOH	Toluene	68.4	53.7	46.3	2α, 2β
82	-50	Glc1α	tBuOH	TfOH	Toluene	43.0	69.3	30.7	4α, 4β
83	-30	Glc1α	tBuOH	TfOH	Toluene	64.7	74.2	25.8	4α, 4β
84	-10	Glc1α	tBuOH	TfOH	Toluene	83.7	75.1	24.9	4α, 4β
85	10	Glc1α	tBuOH	TfOH	Toluene	82.8	74.7	25.3	4α, 4β
86	30	Glc1α	tBuOH	TfOH	Toluene	78.5	72.3	27.7	4α, 4β
87	50	Glc1α	tBuOH	TfOH	Toluene	75.7	68.9	31.1	4α, 4β
88	70	Glc1α	tBuOH	TfOH	Toluene	67.5	65.2	34.8	4α, 4β
89	-30	Glc1α	iPrOH	TfOH	ACN	79.7	9.5	90.5	3α, 3β
90	-10	Glc1α	iPrOH	TfOH	ACN	74.1	14.0	86.0	3α, 3β
91	10	Glc1α	iPrOH	TfOH	ACN	71.4	21.7	78.3	3α, 3β
92	30	Glc1α	iPrOH	TfOH	ACN	75.9	25.1	74.9	3α, 3β
93	50	Glc1α	iPrOH	TfOH	ACN	78.4	31.0	69.0	3α, 3β
94	70	Glc1α	iPrOH	TfOH	ACN	76.9	41.8	58.2	3α, 3β
95	-50	Glc1α	iPrOH	TfOH	MTBE	70.5	84.7	15.3	3α, 3β
96	-30	Glc1α	iPrOH	TfOH	MTBE	72.4	89.5	10.5	3α, 3β
97	-10	Glc1α	iPrOH	TfOH	MTBE	67.7	87.6	12.4	3α, 3β
98	10	Glc1α	iPrOH	TfOH	MTBE	80.6	88.2	11.8	3α, 3β
99	30	Glc1α	iPrOH	TfOH	MTBE	65.2	85.0	15.0	3α, 3β
100	50	Glc1α	iPrOH	TfOH	MTBE	57.3	82.4	17.6	3α, 3β
101	-50	Glc1β	iPrOH	TfOH	MTBE	14.5	95.5	4.5	3α, 3β
102	-30	Glc1β	iPrOH	TfOH	MTBE	55.6	93.3	6.7	3α, 3β
103	-10	Glc1β	iPrOH	TfOH	MTBE	54.6	92.6	7.4	3α, 3β
104	10	Glc1β	iPrOH	TfOH	MTBE	60.0	89.7	10.3	3α, 3β
105	30	Glc1β	iPrOH	TfOH	MTBE	56.9	87.6	12.4	3α, 3β
106	50	Glc1β	iPrOH	TfOH	MTBE	47.5	85.4	14.6	3α, 3β

107	-50	Gal1α	MeOH	TfOH	DCM	98.0	14.3	85.7	5α, 5β
108	-30	Gal1α	MeOH	TfOH	DCM	92.4	30.4	69.6	5α, 5β
109	-10	Gal1α	MeOH	TfOH	DCM	96.5	42.1	57.9	5α, 5β
110	10	Gal1α	MeOH	TfOH	DCM	96.8	52.7	47.3	5α, 5β
111	20	Gal1α	MeOH	TfOH	DCM	94.1	55.6	44.4	5α, 5β
112	30	Gal1α	MeOH	TfOH	DCM	95.2	59.5	40.5	5α, 5β
113	-50	Gal1α	EtOH	TfOH	DCM	92.4	10.7	89.3	6α, 6β
114	-30	Gal1α	EtOH	TfOH	DCM	98.3	21.9	78.1	6α, 6β
115	-10	Gal1α	EtOH	TfOH	DCM	99.0	34.5	65.5	6α, 6β
116	10	Gal1α	EtOH	TfOH	DCM	96.6	44.0	56.0	6α, 6β
117	20	Gal1α	EtOH	TfOH	DCM	98.2	47.9	52.1	6α, 6β
118	30	Gal1α	EtOH	TfOH	DCM	98.2	50.9	49.1	6α, 6β
119	-50	Gal1α	iPrOH	TfOH	DCM	98.2	19.3	80.7	7α, 7β
120	-30	Gal1α	iPrOH	TfOH	DCM	99.7	29.6	70.4	7α, 7β
121	-10	Gal1α	iPrOH	TfOH	DCM	96.4	41.0	59.0	7α, 7β
122	10	Gal1α	iPrOH	TfOH	DCM	96.9	46.4	53.6	7α, 7β
123	20	Gal1α	iPrOH	TfOH	DCM	95.0	49.3	50.7	7α, 7β
124	30	Gal1α	iPrOH	TfOH	DCM	78.0	51.4	48.6	7α, 7β
125	-50	Gal1α	tBuOH	TfOH	DCM	51.7	36.1	63.9	8α, 8β
126	-30	Gal1α	tBuOH	TfOH	DCM	99.4	40.3	59.7	8α, 8β
127	-10	Gal1α	tBuOH	TfOH	DCM	100.0	45.6	54.4	8α, 8β
128	10	Gal1α	tBuOH	TfOH	DCM	82.5	50.2	49.8	8α, 8β
129	20	Gal1α	tBuOH	TfOH	DCM	77.9	54.7	45.3	8α, 8β
130	30	Gal1α	tBuOH	TfOH	DCM	74.1	57.2	42.8	8α, 8β
131	-30	Gal2β	iPrOH	TfOH	DCM	15.9	38.6	61.4	7α, 7β
132	-10	Gal2β	iPrOH	TfOH	DCM	87.1	42.1	57.9	7α, 7β
133	10	Gal2β	iPrOH	TfOH	DCM	75.3	45.6	54.4	7α, 7β
134	20	Gal2β	iPrOH	TfOH	DCM	76.6	50.2	49.8	7α, 7β
135	30	Gal2β	iPrOH	TfOH	DCM	78.6	52.8	47.2	7α, 7β
136	-50	Gal1α	2F-EtOH	TfOH	DCM	76.8	62.6	37.4	9α, 9β
137	-30	Gal1α	2F-EtOH	TfOH	DCM	78.3	72.7	27.3	9α, 9β
138	-10	Gal1α	2F-EtOH	TfOH	DCM	83.3	70.6	29.4	9α, 9β
139	10	Gal1α	2F-EtOH	TfOH	DCM	84.1	71.3	28.7	9α, 9β
140	20	Gal1α	2F-EtOH	TfOH	DCM	93.5	70.8	29.2	9α, 9β
141	30	Gal1α	2F-EtOH	TfOH	DCM	86.0	70.7	29.3	9α, 9β
142	-50	Gal1α	iPrOH	TfOH	Toluene	96.7	10.3	89.7	7α, 7β
143	-30	Gal1α	iPrOH	TfOH	Toluene	93.1	14.7	85.3	7α, 7β
144	-10	Gal1α	iPrOH	TfOH	Toluene	95.6	27.0	73.0	7α, 7β
145	10	Gal1α	iPrOH	TfOH	Toluene	96.7	41.5	58.5	7α, 7β
146	30	Gal1α	iPrOH	TfOH	Toluene	90.6	57.3	42.7	7α, 7β
147	50	Gal1α	iPrOH	TfOH	Toluene	85.6	66.5	33.5	7α, 7β
148	70	Gal1α	iPrOH	TfOH	Toluene	77.0	68.9	31.1	7α, 7β
149	-50	Gal1α	iPrOH	TfOH	MTBE	39.6	43.6	56.4	7α, 7β
150	-30	Gal1α	iPrOH	TfOH	MTBE	63.5	55.9	44.1	7α, 7β
151	-10	Gal1α	iPrOH	TfOH	MTBE	85.3	67.1	32.9	7α, 7β
152	10	Gal1α	iPrOH	TfOH	MTBE	86.2	72.0	28.0	7α, 7β
153	30	Gal1α	iPrOH	TfOH	MTBE	81.5	76.0	24.0	7α, 7β
154	50	Gal1α	iPrOH	TfOH	MTBE	79.9	77.8	22.2	7α, 7β
155	-30	Gal1α	iPrOH	TfOH	ACN	29.8	16.1	83.9	7α, 7β
156	-10	Gal1α	iPrOH	TfOH	ACN	73.1	22.0	78.0	7α, 7β
157	10	Gal1α	iPrOH	TfOH	ACN	73.4	24.9	75.1	7α, 7β
158	30	Gal1α	iPrOH	TfOH	ACN	74.7	29.1	70.9	7α, 7β
159	50	Gal1α	iPrOH	TfOH	ACN	69.8	34.6	65.4	7α, 7β
160	70	Gal1α	iPrOH	TfOH	ACN	63.9	38.6	61.4	7α, 7β

161	-50	Man1α	MeOH	TfOH	DCM	96.8	50.6	49.4	10α, 10β
162	-30	Man1α	MeOH	TfOH	DCM	96.0	54.1	45.9	10α, 10β
163	-10	Man1α	MeOH	TfOH	DCM	97.5	57.4	42.6	10α, 10β
164	10	Man1α	MeOH	TfOH	DCM	92.1	59.6	40.4	10α, 10β
165	20	Man1α	MeOH	TfOH	DCM	75.5	64.1	35.9	10α, 10β
166	30	Man1α	MeOH	TfOH	DCM	86.7	64.7	35.3	10α, 10β
167	-50	Man1α	EtOH	TfOH	DCM	99.8	47.9	52.1	11α, 11β
168	-30	Man1α	EtOH	TfOH	DCM	97.3	53.1	46.9	11α, 11β
169	-10	Man1α	EtOH	TfOH	DCM	93.8	56.5	43.5	11α, 11β
170	10	Man1α	EtOH	TfOH	DCM	91.9	59.4	40.6	11α, 11β
171	20	Man1α	EtOH	TfOH	DCM	92.1	59.5	40.5	11α, 11β
172	30	Man1α	EtOH	TfOH	DCM	90.0	59.3	40.7	11α, 11β
173	-50	Man1α	iPrOH	TfOH	DCM	98.1	49.9	50.1	12α, 12β
174	-30	Man1α	iPrOH	TfOH	DCM	95.8	53.1	46.9	12α, 12β
175	-10	Man1α	iPrOH	TfOH	DCM	99.1	56.5	43.5	12α, 12β
176	10	Man1α	iPrOH	TfOH	DCM	99.0	59.4	40.6	12α, 12β
177	20	Man1α	iPrOH	TfOH	DCM	98.7	59.5	40.5	12α, 12β
178	30	Man1α	iPrOH	TfOH	DCM	93.9	64.9	35.1	12α, 12β
179	-50	Man1α	tBuOH	TfOH	DCM	99.4	55.3	44.7	13α, 13β
180	-30	Man1α	tBuOH	TfOH	DCM	92.7	57.6	42.4	13α, 13β
181	-10	Man1α	tBuOH	TfOH	DCM	81.0	61.5	38.5	13α, 13β
182	10	Man1α	tBuOH	TfOH	DCM	78.6	66.1	33.9	13α, 13β
183	20	Man1α	tBuOH	TfOH	DCM	78.3	74.9	25.1	13α, 13β
184	30	Man1α	tBuOH	TfOH	DCM	74.9	94.6	5.4	13α, 13β
185	-50	Man1α	tBuOH	FSO3H	DCM	96.4	55.0	45.0	13α, 13β
186	-30	Man1α	tBuOH	FSO3H	DCM	96.5	57.4	42.6	13α, 13β
187	-10	Man1α	tBuOH	FSO3H	DCM	94.6	57.8	42.2	13α, 13β
188	10	Man1α	tBuOH	FSO3H	DCM	90.7	59.2	40.8	13α, 13β
189	30	Man1α	tBuOH	FSO3H	DCM	100.0	61.3	38.7	13α, 13β
190	-50	Man1α	tBuOH	MsOH	DCM	50.2	57.5	42.5	13α, 13β
191	-30	Man1α	tBuOH	MsOH	DCM	50.4	60.2	39.8	13α, 13β
192	-10	Man1α	tBuOH	MsOH	DCM	50.1	61.2	38.8	13α, 13β
193	10	Man1α	tBuOH	MsOH	DCM	51.6	63.3	36.7	13α, 13β
194	30	Man1α	tBuOH	MsOH	DCM	49.7	63.6	36.4	13α, 13β
195	-40	Man1α	tBuOH	Tf2NH	DCM	67.7	55.5	44.5	13α, 13β
196	-30	Man1α	tBuOH	Tf2NH	DCM	81.4	55.1	44.9	13α, 13β
197	-10	Man1α	tBuOH	Tf2NH	DCM	65.3	62.3	37.7	13α, 13β
198	-5	Man1α	tBuOH	Tf2NH	DCM	65.4	71.4	28.6	13α, 13β
199	5	Man1α	tBuOH	Tf2NH	DCM	61.8	93.7	6.3	13α, 13β
200	10	Man1α	tBuOH	Tf2NH	DCM	61.3	97.7	2.3	13α, 13β
201	20	Man1α	tBuOH	Tf2NH	DCM	56.8	97.3	2.7	13α, 13β
202	30	Man1α	tBuOH	Tf2NH	DCM	45.9	97.6	2.4	13α, 13β
203	-50	Man1α	EtOH	Tf2NH	DCM	74.3	54.8	45.2	11α, 11β
204	-30	Man1α	EtOH	Tf2NH	DCM	70.5	57.0	43.0	11α, 11β
205	-10	Man1α	EtOH	Tf2NH	DCM	69.1	57.0	43.0	11α, 11β
206	10	Man1α	EtOH	Tf2NH	DCM	66.7	61.7	38.3	11α, 11β
207	30	Man1α	EtOH	Tf2NH	DCM	45.4	62.3	37.7	11α, 11β
208	-10	Man2α	iPrOH	TfOH	DCM	21.7	53.8	46.2	12α, 12β
209	10	Man2α	iPrOH	TfOH	DCM	67.9	57.0	43.0	12α, 12β
210	20	Man2α	iPrOH	TfOH	DCM	73.6	57.5	42.5	12α, 12β
211	30	Man2α	iPrOH	TfOH	DCM	76.0	58.0	42.0	12α, 12β

212	-50	Man1α	iPrOH	TfOH	Toluene	67.8	61.2	38.8	12α, 12β
213	-30	Man1α	iPrOH	TfOH	Toluene	98.1	63.8	36.2	12α, 12β
214	-10	Man1α	iPrOH	TfOH	Toluene	100.0	66.7	33.3	12α, 12β
215	10	Man1α	iPrOH	TfOH	Toluene	95.2	70.2	29.8	12α, 12β
216	30	Man1α	iPrOH	TfOH	Toluene	94.7	74.4	25.6	12α, 12β
217	50	Man1α	iPrOH	TfOH	Toluene	90.5	78.5	21.5	12α, 12β
218	70	Man1α	iPrOH	TfOH	Toluene	77.7	87.1	12.9	12α, 12β
219	-30	Man1α	iPrOH	TfOH	ACN	70.8	64.9	35.1	12α, 12β
220	-10	Man1α	iPrOH	TfOH	ACN	70.5	65.9	34.1	12α, 12β
221	10	Man1α	iPrOH	TfOH	ACN	69.4	74.4	25.6	12α, 12β
222	20	Man1α	iPrOH	TfOH	ACN	68.5	91.3	8.7	12α, 12β
223	30	Man1α	iPrOH	TfOH	ACN	79.1	97.1	2.9	12α, 12β
224	50	Man1α	iPrOH	TfOH	ACN	75.5	98.6	1.4	12α, 12β
225	70	Man1α	iPrOH	TfOH	ACN	70.7	98.2	1.8	12α, 12β
226	-50	Man1α	iPrOH	TfOH	MTBE	44.6	72.0	28.0	12α, 12β
227	-30	Man1α	iPrOH	TfOH	MTBE	46.7	75.1	24.9	12α, 12β
228	-10	Man1α	iPrOH	TfOH	MTBE	65.9	76.4	23.6	12α, 12β
229	10	Man1α	iPrOH	TfOH	MTBE	55.2	79.6	20.4	12α, 12β
230	30	Man1α	iPrOH	TfOH	MTBE	54.6	81.6	18.4	12α, 12β
231	50	Man1α	iPrOH	TfOH	MTBE	51.7	83.9	16.1	12α, 12β
232	-50	Man1α	3F-EtOH	TfOH	DCM	95.5	100	0	14α
233	-30	Man1α	3F-EtOH	TfOH	DCM	99.4	100	0	14α
234	-10	Man1α	3F-EtOH	TfOH	DCM	91.1	100	0	14α
235	10	Man1α	3F-EtOH	TfOH	DCM	91.8	100	0	14α
236	20	Man1α	3F-EtOH	TfOH	DCM	93.1	100	0	14α
237	30	Man1α	3F-EtOH	TfOH	DCM	92.7	100	0	14α
238	-30	Gal1α	iPrOH	TfOH	Anisole	61.9	65.4	34.6	7α, 7β
239	-10	Gal1α	iPrOH	TfOH	Anisole	68.0	58.2	41.8	7α, 7β
240	10	Gal1α	iPrOH	TfOH	Anisole	65.9	61.6	38.4	7α, 7β
241	30	Gal1α	iPrOH	TfOH	Anisole	62.5	65.5	34.5	7α, 7β
242	50	Gal1α	iPrOH	TfOH	Anisole	52.2	69.1	30.9	7α, 7β
243	70	Gal1α	iPrOH	TfOH	Anisole	43.5	70.2	29.8	7α, 7β
244	-30	Man1α	iPrOH	TfOH	Anisole	94.5	57.8	42.2	12α, 12β
245	-10	Man1α	iPrOH	TfOH	Anisole	85.3	61.3	38.7	12α, 12β
246	10	Man1α	iPrOH	TfOH	Anisole	71.9	64.5	35.5	12α, 12β
247	30	Man1α	iPrOH	TfOH	Anisole	54.4	66.3	33.7	12α, 12β
248	50	Man1α	iPrOH	TfOH	Anisole	51.0	66.2	33.8	12α, 12β
249	70	Man1α	iPrOH	TfOH	Anisole	60.3	49.1	50.9	12α, 12β
250	-50	Glc1α	iPrOH	TfOH	tBu-Benzene	59.5	17.4	82.6	3α, 3β
251	-30	Glc1α	iPrOH	TfOH	tBu-Benzene	67.7	25.9	74.1	3α, 3β
252	-10	Glc1α	iPrOH	TfOH	tBu-Benzene	98.9	39.6	60.4	3α, 3β
253	10	Glc1α	iPrOH	TfOH	tBu-Benzene	96.9	47.2	52.8	3α, 3β
254	30	Glc1α	iPrOH	TfOH	tBu-Benzene	95.3	54	46	3α, 3β
255	50	Glc1α	iPrOH	TfOH	tBu-Benzene	79.0	58.2	41.8	3α, 3β
256	70	Glc1α	iPrOH	TfOH	tBu-Benzene	77.0	60.1	39.9	3α, 3β
257	-50	Glc1α	iPrOH	TfOH	Chloroform	67.1	37.4	62.6	3α, 3β
258	-30	Glc1α	iPrOH	TfOH	Chloroform	86.6	47.5	52.5	3α, 3β
259	-10	Glc1α	iPrOH	TfOH	Chloroform	89.5	58.1	41.9	3α, 3β
260	10	Glc1α	iPrOH	TfOH	Chloroform	97.9	64.3	35.7	3α, 3β
261	30	Glc1α	iPrOH	TfOH	Chloroform	96.3	70.2	29.8	3α, 3β
262	50	Glc1α	iPrOH	TfOH	Chloroform	93.1	72.9	27.1	3α, 3β
263	-50	Glc1β	iPrOH	TfOH	Chloroform	72.7	37.6	62.4	3α, 3β
264	-30	Glc1β	iPrOH	TfOH	Chloroform	81.2	46.2	53.8	3α, 3β
265	-10	Glc1β	iPrOH	TfOH	Chloroform	88.6	54.6	45.4	3α, 3β
266	10	Glc1β	iPrOH	TfOH	Chloroform	84.3	62.9	37.1	3α, 3β
267	30	Glc1β	iPrOH	TfOH	Chloroform	88.3	67.3	32.7	3α, 3β
268	50	Glc1β	iPrOH	TfOH	Chloroform	89.1	73.0	27.0	3α, 3β

New Electrophile, Nucleophile, Activator and Solvent

The following compounds, organized by electrophile/nucleophile/activator/solvent, were not included in the training set and used as out-of-sample examples of each category. Predictions were run of glycosylations using these chemicals. These predictions were subsequently validated experimentally on the same microreactor platform.

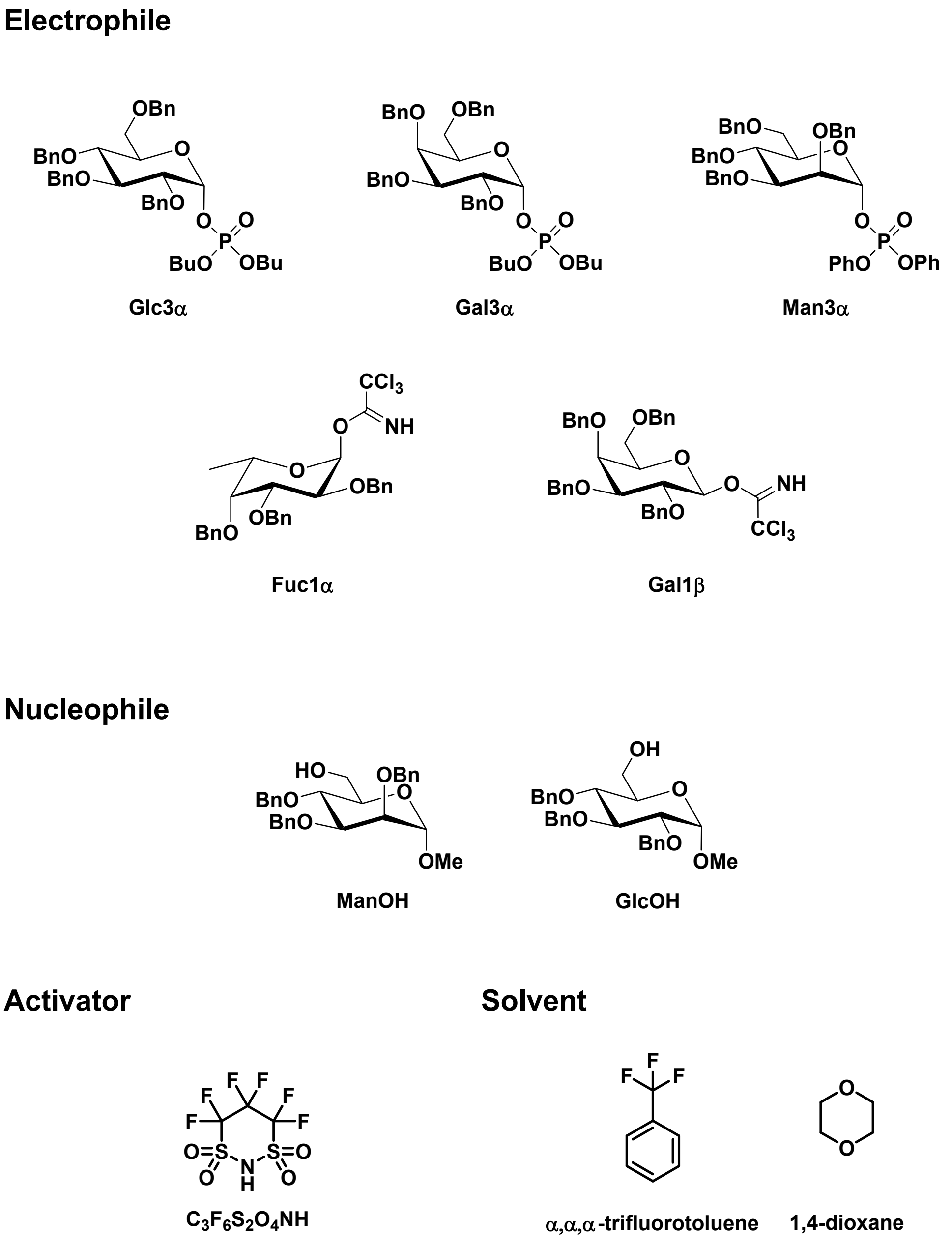

Figure S2: Out-of-sample chemicals used to validate prediction accuracy of the model with variances for each chemical category: electrophiles, nucleophiles, an activator, and solvents.

Quantification of descriptors

As described in the text, the relevant properties of the electrophile are quantified by three descriptors. The values of these descriptors for electrophiles in both the training and holdout sets are provided in Figure S3 and Table S2.

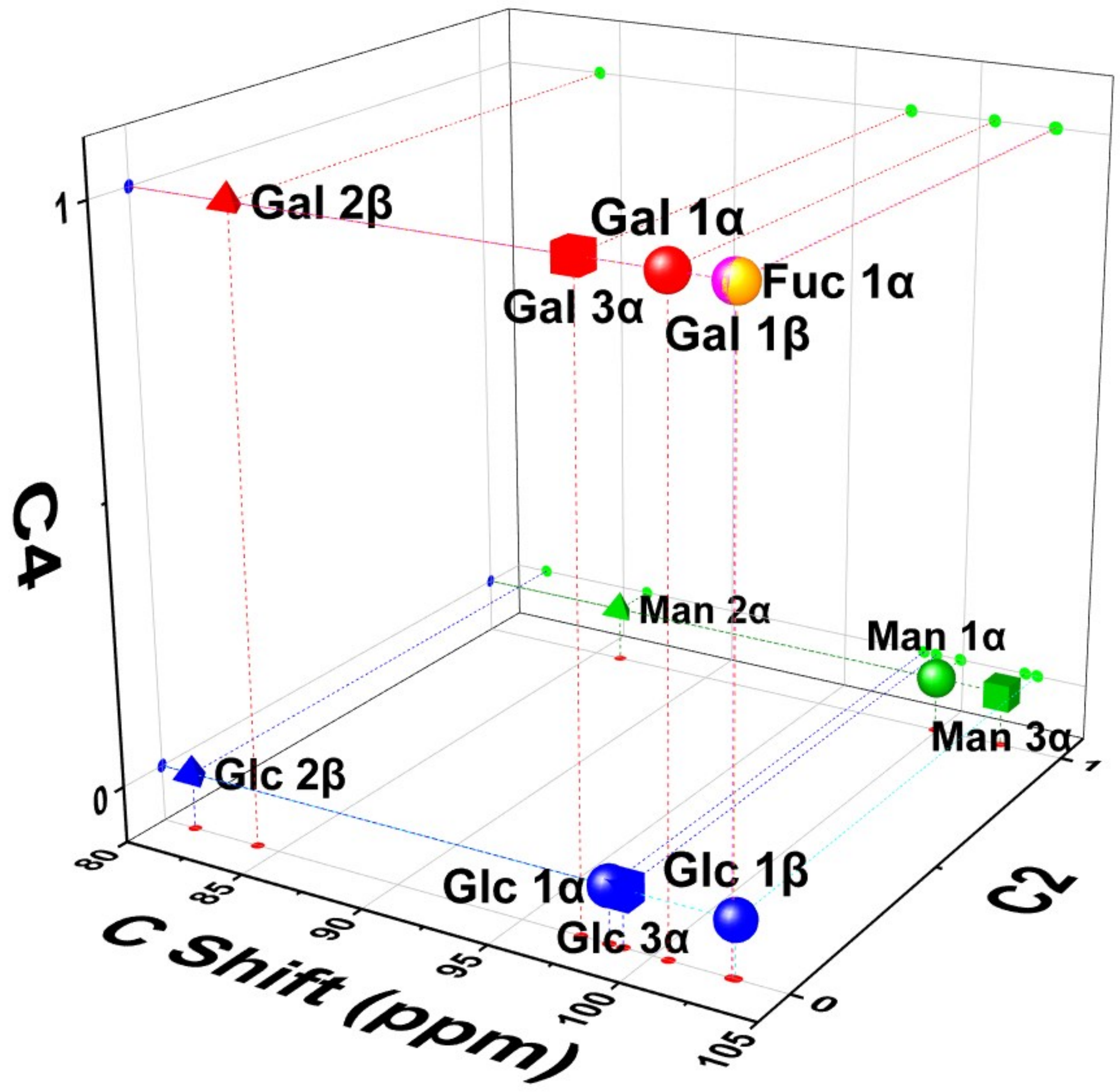

Figure S3: 3D map of total electrophile chemical subspace (X: [13]Carbon NMR chemical shift on C1 (ppm), Y: the orientation of C2 substituents on the pyran ring, Z: the orientation of C4 substituents on the pyran ring, axial – 1, equatorial – 0). Basis set: B3LYP 6-31G* level of theory.

Electrophile	C Shift (ppm)	C2	C4
Glc1α	98.357	0	0
Glc 1β	103.043	0	0
Gal 1α	100.581	0	1
Gal 1β	102.96	0	1
Man 1α	99.895	1	0
Glc 2β	81.354	0	0
Gal 2β	84.093	0	1
Man 2α	86.109	1	0
Glc 3α	98.888	0	0
Gal 3α	97.28	0	1
Man 3α	102.558	1	0
Fuc 1α	102.907	0	1

Table S2: The value of descriptors for electrophiles.

As described in the text, the relevant properties of the nucleophile are quantified by three descriptors. The values of these descriptors for nucleophiles in both the training and validation set are provided in Figure S4 and Table S3.

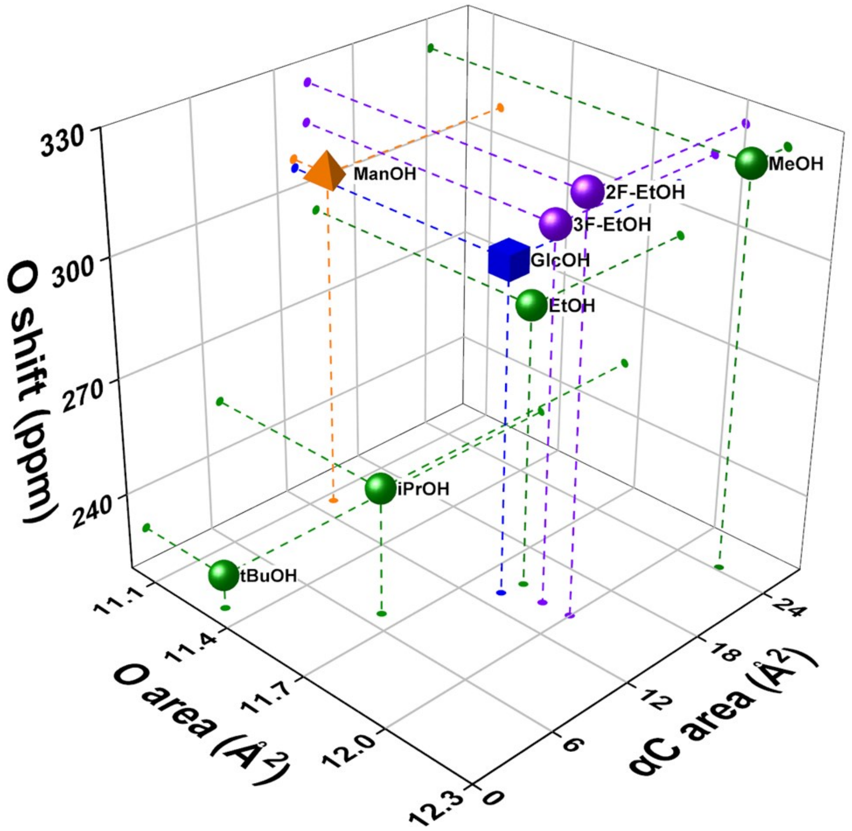

Figure S4: 3D map of nucleophile chemical subspace (X: exposed surface area (Å^2) of oxygen in a space-filling model, Y: exposed surface area (Å^2) of α-carbon in a space-filling model, Z: ^{17}Oxygen NMR chemical shift of hydroxyl group of nucleophile). Basis set: B3LYP 6-311G* level of theory.

Acceptor	O area (Å^2)	αC area (Å^2)	O shift (ppm)
MeOH	12.136	24.304	322.409
EtOH	11.811	14.908	292.11
iPrOH	11.64	7.066	253.13
tBuOH	11.327	1.196	228.836
2F-EtOH	11.999	14.469	324.373
3F-EtOH	11.907	14.32	314.757
GlcOH	11.797	13.355	304.638
ManOH	11.134	13.276	306.933

Table S3: The value of descriptors for nucleophiles.

As described in the text, the relevant properties of the solvent are quantified by two descriptors. The values of these descriptors for solvents in both the training and validation set are provided in Figure S5a and Table S4a. The relevant properties of the activator are quantified by two descriptors. The values of these descriptors for activators in both the training and validation set are provided in Figure S5b and Table S4b.

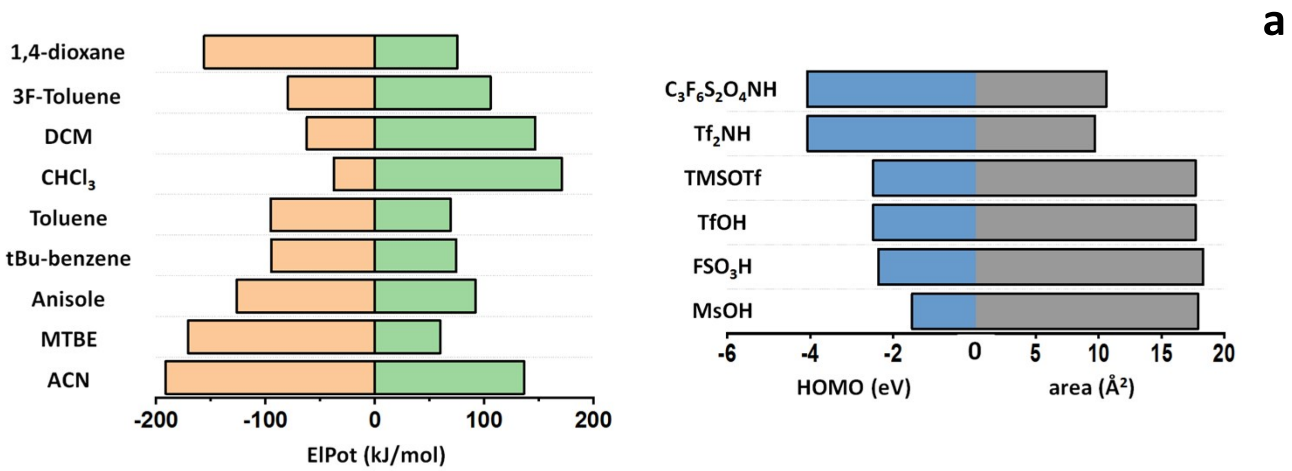

Figure S5: **a**, Plot of solvent descriptors, value of the electrostatic potential (kJ/mol). **b**, Plot of activator descriptors, Left: HOMO: highest occupied molecular orbital (eV) of the conjugate base. Right: oxygen (O^-) or nitrogen anion (N^-) exposed surface area (Å^2) in a space-filling model of the conjugate base. Basis set: B3LYP 6-311G* level of theory.

a

Solvent	Min_EiPot (kJ/mol)	Max_EiPot (kJ/mol)
1,4-dioxane	-156.08	75.7
3F-Toluene	-79.32	105.79
DCM	-62.13	146.58
$CHCl_3$	-37.44	170.92
Toluene	-95.18	69.25
tBu-Benzene	-94.82	74.38
Anisole	-126.36	92.35
MTBE	-170.72	59.82
ACN	-191.22	136.7

b

Activator	HOMO (eV)	O^-/N^-area (Å^2)
$C_3F_6S_2O_4NH$	-4.07	10.6
Tf_2NH	-4.06	9.7
TMSOTf	-2.48	17.7
TfOH	-2.48	17.7
FSO_3H	-2.36	18.3
MsOH	-1.54	17.9

Table S4: The value of descriptors for solvents (Left) and activators (Right).

New Test Set

The experimental results for the out-of-sample glycosylations involving electrophiles bearing a phosphate leaving group are given in Table S5. The accuracy of the random forest algorithm is depicted in Figure S6 and compared to three other algorithms: regression tree, Gaussian process regression, and support vector machine. The TMSOTf activator uses the same descriptors as calculated for TfOH, as they describe the conjugate base (triflate anion).

Prediction of phosphate leaving group

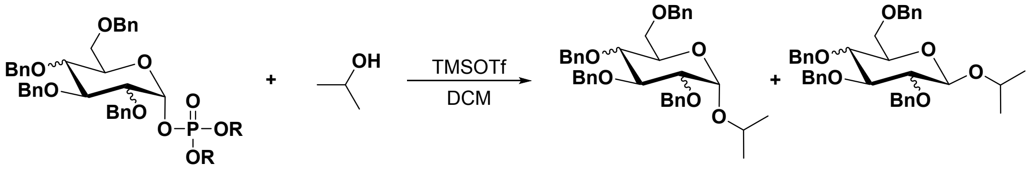

Glc3α (R = *n*-Bu) **3α** **3β**
Gal3α (R = *n*-Bu) **7α** **7β**
Man3α (R = Ph) **12α** **12β**

Entry	Temp. (°C)	Donor	Acceptor	Activator	Solvent	Yield (%)	α ratio (%)	β ratio (%)	Product
269	-30	Glc3α	iPrOH	TMSOTf	DCM	29.5	31.3	68.7	3α, 3β
270	-10	Glc3α	iPrOH	TMSOTf	DCM	60.1	44.7	55.3	3α, 3β
271	10	Glc3α	iPrOH	TMSOTf	DCM	79.1	53.2	46.8	3α, 3β
272	20	Glc3α	iPrOH	TMSOTf	DCM	90.4	57.9	42.1	3α, 3β
273	30	Glc3α	iPrOH	TMSOTf	DCM	98.6	61.5	38.5	3α, 3β
274	-30	Gal3α	iPrOH	TMSOTf	DCM	33.9	34.3	65.7	7α, 7β
275	-10	Gal3α	iPrOH	TMSOTf	DCM	49.2	41.5	58.5	7α, 7β
276	10	Gal3α	iPrOH	TMSOTf	DCM	79.2	47.3	52.7	7α, 7β
277	20	Gal3α	iPrOH	TMSOTf	DCM	93.1	50.1	49.9	7α, 7β
278	30	Gal3α	iPrOH	TMSOTf	DCM	98.1	52.7	47.3	7α, 7β
279	20	Man3α	iPrOH	TMSOTf	DCM	37.5	63.9	36.1	12α, 12β
280	30	Man3α	iPrOH	TMSOTf	DCM	81.1	62.1	37.9	12α, 12β

Table S5: Validation data collected from the automated flow platform to predict phosphate leaving group with glucose, galactose and mannose.

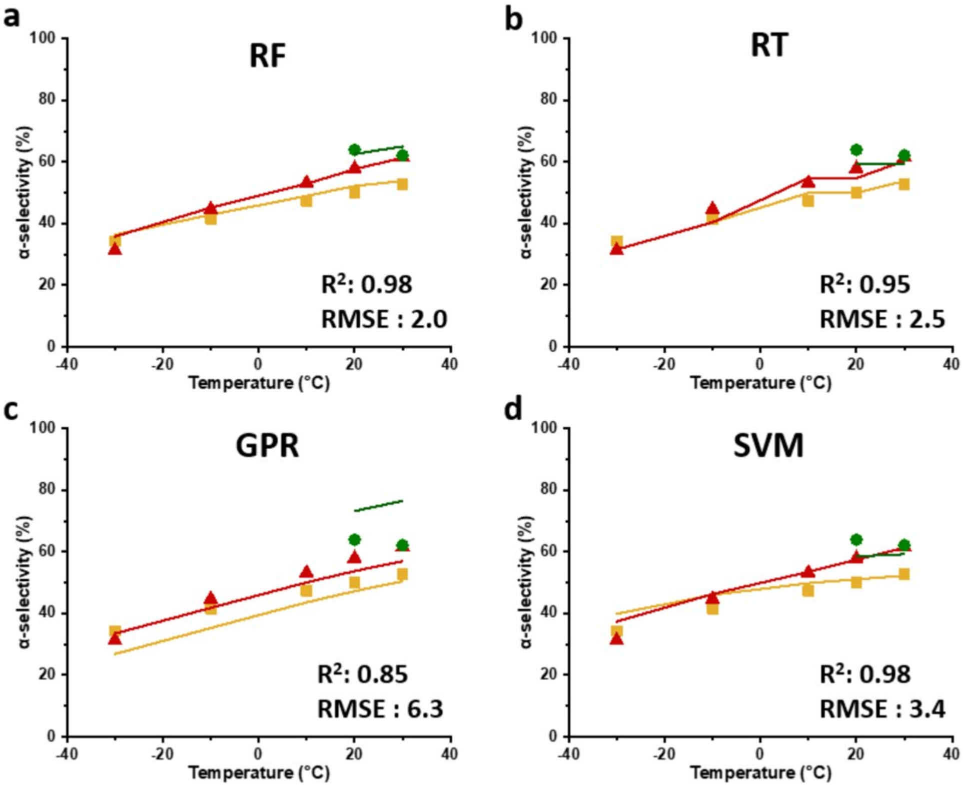

Temp. (°C)	Experimental	RF	RT	GPR	SVM
-30	31.3	35.8	31.6	33.5	37.4
-10	44.7	45.2	40.4	41.8	46.3
10	53.2	52.9	54.7	50.1	53.5
20	57.9	57.6	54.7	53.8	57.5
30	61.5	61.4	60.3	57.0	61.3
-30	34.3	36.4	31.6	26.9	39.9
-10	41.5	42.9	40.4	35.3	46.0
10	47.3	49.0	49.9	43.6	49.8
20	50.1	52.2	49.9	47.3	51.1
30	52.7	53.9	53.8	50.5	52.3
20	63.9	62.6	59.4	73.2	58.5
30	62.1	65.0	59.4	76.5	59.2

Figure S6: **a**, Prediction with Random Forest (RF). **b**, Prediction with Regression Tree (RT). **c**, Prediction with Gaussian Process Regression (GPR). **d**, Prediction with Support Vector Machine (SVR). Glucose (□, red); Galactose (○, yellow); Mannose (✿, green); experimental (data points); predicted (solid colored line).

Prediction of Electrophile- **Fucose**

The experimental results for the out-of-sample glycosylations involving a fucose electrophile are given in Table S6. The accuracy of the random forest algorithm is depicted in Figure S7 and compared to three other algorithms: regression tree, Gaussian process regression, and support vector machine. The HPLC calibration curve for the product is given in Figure S8.

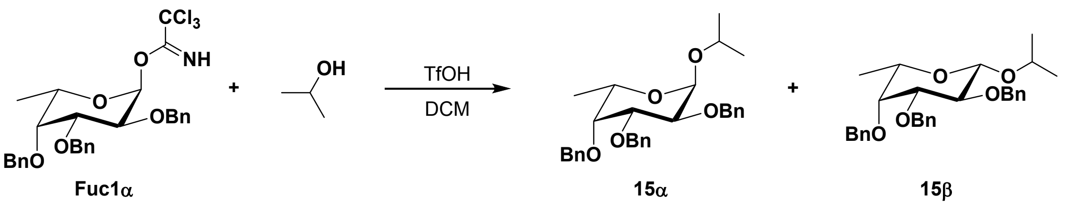

Entry	Temp. (°C)	Donor	Acceptor	Activator	Solvent	Yield (%)	α ratio (%)	β ratio (%)	Product
281	-50	Fuc1α	iPrOH	TfOH	DCM	71.7	19.9	80.1	15α, 15β
282	-30	Fuc1α	iPrOH	TfOH	DCM	69.4	30	70	15α, 15β
283	-10	Fuc1α	iPrOH	TfOH	DCM	71.8	33.6	66.4	15α, 15β
284	10	Fuc1α	iPrOH	TfOH	DCM	56.3	39.7	60.3	15α, 15β

Table S6: Validation data collected from the automated flow platform to predict fucose imidate electrophile.

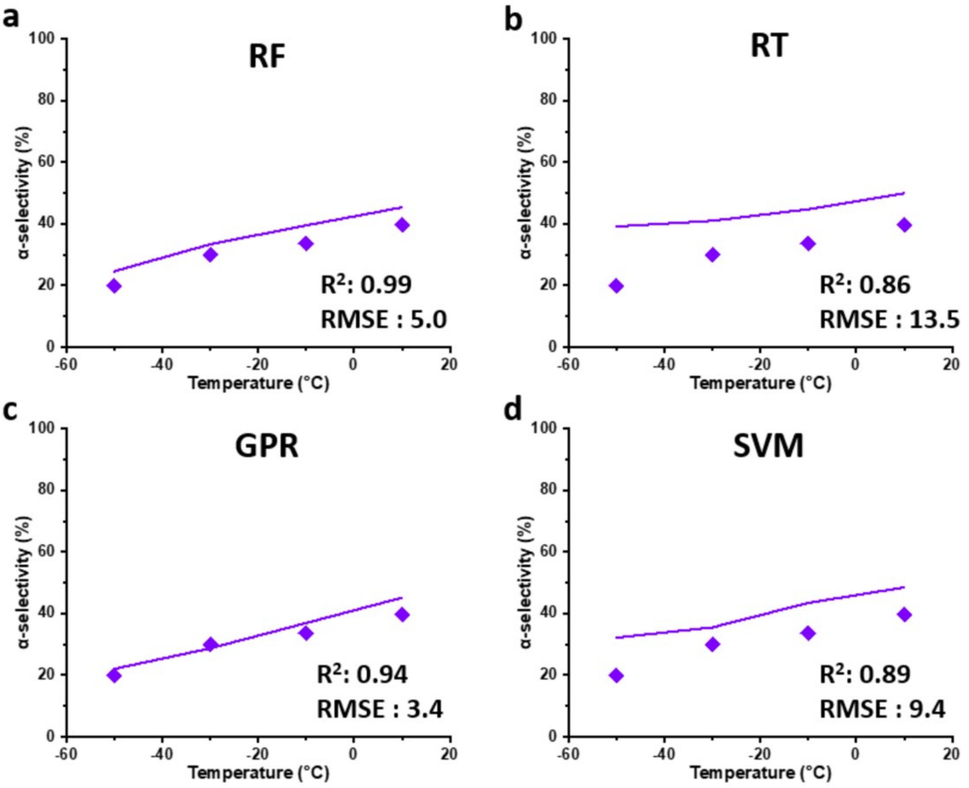

Temp. (°C)	Experimental	RF	RT	GPR	SVM
-50	19.9	24.6	39.2	22.1	32.2
-30	30	33.4	41.0	28.7	35.5
-10	33.6	39.5	44.8	37.0	43.5
10	39.7	45.4	49.9	45.2	48.5

Figure S7: **a**, Prediction with Random Forest (RF). **b**, Prediction with Regression Tree (RT). **c**, Prediction with Gaussian Process Regression (GPR). **d**, Prediction with Support Vector Machine (SVR). experimental (data points); predicted (solid colored line).

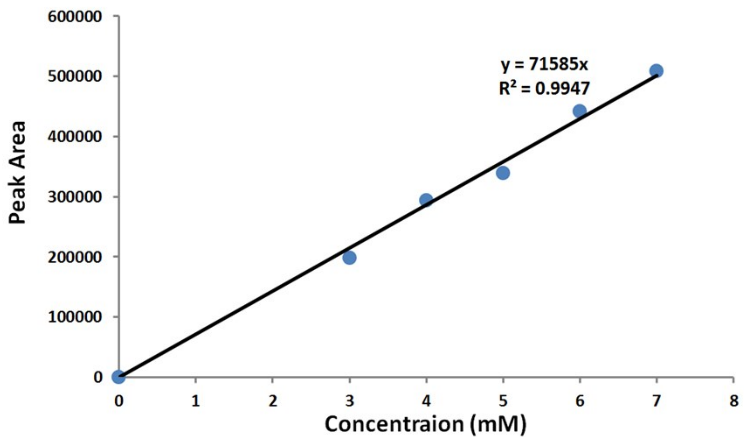

Figure S8: HPLC calibration curve of **15αβ**.

Prediction of Nucleophile- **Mannose**

The experimental results for the out-of-sample glycosylations involving a C6 mannose glycosyl nucleophiles are given in Table S7. The accuracy of the random forest algorithm is depicted in Figure S9 and compared to three other algorithms: regression tree, Gaussian process regression, and support vector machine.

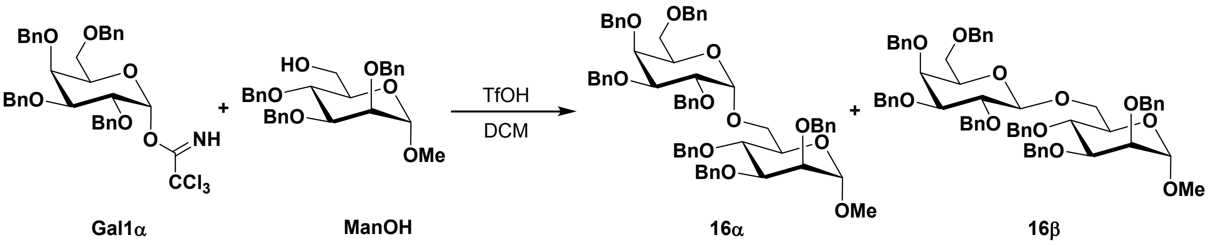

Entry	Temp. (°C)	Donor	Acceptor	Activator	Solvent	Yield (%)	α ratio (%)	β ratio (%)	Product
285	-50	Gal1α	ManOH	TfOH	DCM	95.4	48.9	51.1	16α, 16β
286	-30	Gal1α	ManOH	TfOH	DCM	92.0	56.5	43.5	16α, 16β
287	-10	Gal1α	ManOH	TfOH	DCM	84.1	61.5	38.5	16α, 16β
288	10	Gal1α	ManOH	TfOH	DCM	82.9	65.7	34.3	16α, 16β
289	30	Gal1α	ManOH	TfOH	DCM	76.2	69.7	30.3	16α, 16β

Table S7: Validation data collected from the automated flow platform to predict mannose nucleophile.

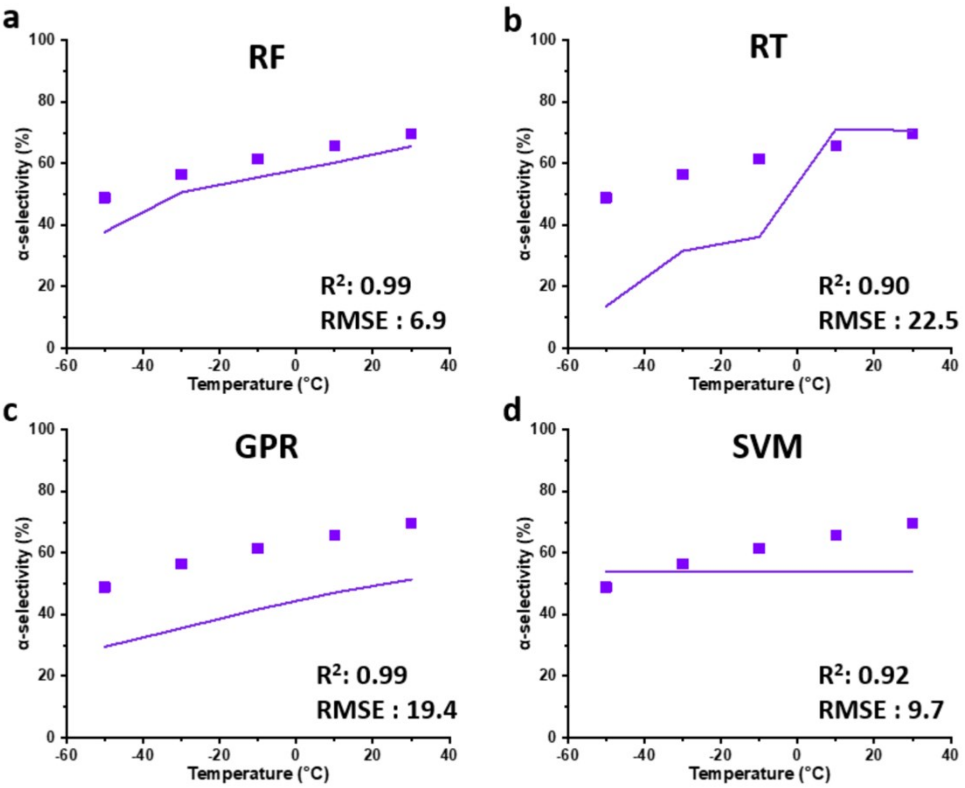

Temp. (°C)	Experimental	RF	RT	GPR	SVM
-50	48.9	37.8	13.6	29.6	54.0
-30	56.5	50.6	31.6	35.5	54.0
-10	61.5	55.5	36.2	41.7	54.0
10	65.7	60.3	71.1	47.1	54.0
30	69.7	65.5	70.7	51.4	54.0

Figure S9: **a**, Prediction with Random Forest (RF). **b**, Prediction with Regression Tree (RT). **c**, Prediction with Gaussian Process Regression (GPR). **d**, Prediction with Support Vector Machine (SVR). experimental (data points); predicted (solid colored line).

Prediction of Nucleophile- **Glucose**

The experimental results for the out-of-sample glycosylations involving a C6 glucose glycosyl nucleophiles are given in Table S8. The accuracy of the random forest algorithm is depicted in Figure S10 and compared to three other algorithms: regression tree, Gaussian process regression, and support vector machine. The HPLC calibration curve for the product is given in Figure S11.

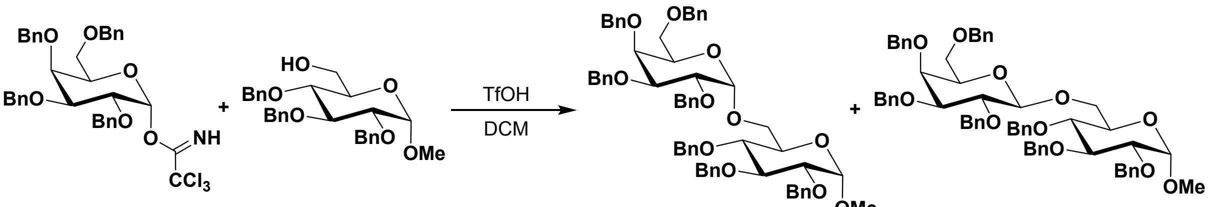

Gal1α GluOH 17α 17β

Entry	Temp. (°C)	Donor	Acceptor	Activator	Solvent	Yield (%)	α ratio (%)	β ratio (%)	Product
290	-50	Gal1α	GlcOH	TfOH	DCM	98.1	42.4	57.6	17α, 17β
291	-30	Gal1α	GlcOH	TfOH	DCM	93.6	51.9	48.1	17α, 17β
292	-10	Gal1α	GlcOH	TfOH	DCM	94.1	62.6	37.4	17α, 17β
293	10	Gal1α	GlcOH	TfOH	DCM	93.2	67.1	32.9	17α, 17β
294	20	Gal1α	GlcOH	TfOH	DCM	88.1	69.2	30.8	17α, 17β
295	30	Gal1α	GlcOH	TfOH	DCM	93.2	71.9	28.1	17α, 17β

Table S8: Validation data collected from the automated flow platform to predict glucose nucleophile.

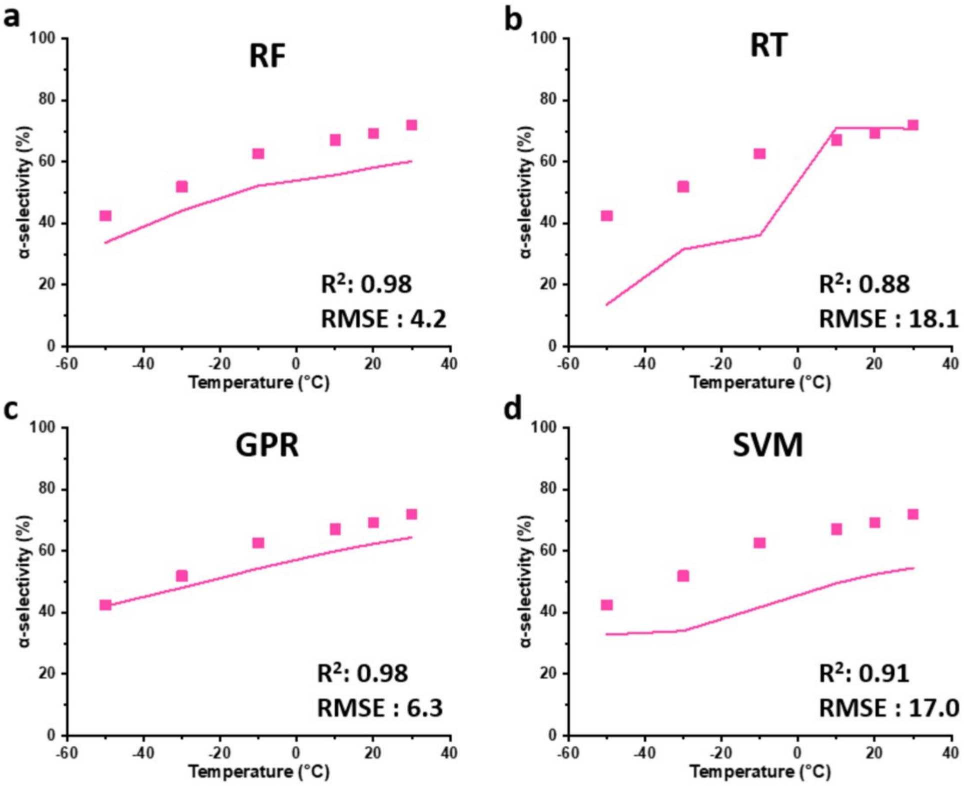

Temp. (°C)	Experimental	RF	RT	GPR	SVM
-50	42.4	38.0	13.6	42.1	32.9
-30	51.9	51.0	31.6	48.1	34.1
-10	62.6	56.9	36.2	54.4	41.7
10	67.1	61.9	71.1	60.0	49.6
20	69.2	65.8	71.1	62.4	52.5
30	71.9	68.4	70.7	64.4	54.5

Figure S10: **a**, Prediction with Random Forest (RF). **b**, Prediction with Regression Tree (RT). **c**, Prediction with Gaussian Process Regression (GPR). **d**, Prediction with Support Vector Machine (SVR). experimental (data points); predicted (solid colored line).

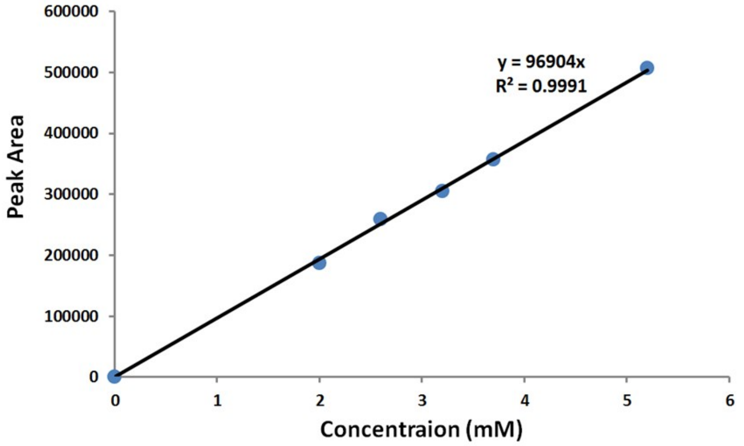

Figure S11: HPLC calibration curve of **17αβ**.

Prediction of Activator- **4,4,5,5,6,6-hexafluoro-1,3,2-dithiazinane 1,1,3,3-tetraoxide with Galactose Electrophile**

The experimental results for the out-of-sample glycosylations involving super acid 4,4,5,5,6,6-hexafluoro-1,3,2-dithiazinane 1,1,3,3-tetraoxide as activator coupling a galactose imidate electrophile and isopropanol as nucleophile are given in Table S9. The accuracy of the random forest algorithm is depicted in Figure S12 and compared to three other algorithms: regression tree, Gaussian process regression, and support vector machine.

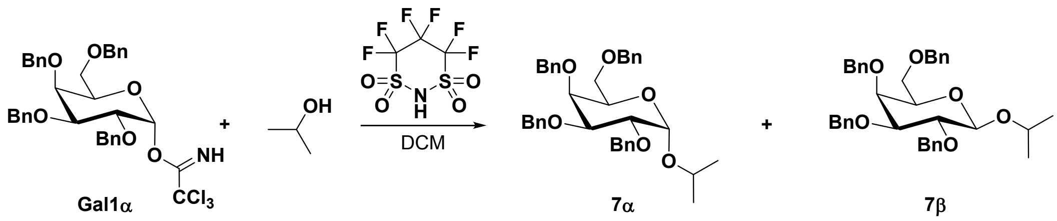

Entry	Temp. (°C)	Donor	Acceptor	Activator	Solvent	Yield (%)	α ratio (%)	β ratio (%)	Product
296	-50	Gal1α	iPrOH	$C_3F_6S_2O_4NH$	DCM	33.0	18.5	81.5	7α, 7β
297	-30	Gal1α	iPrOH	$C_3F_6S_2O_4NH$	DCM	37.4	26.2	73.8	7α, 7β
298	-10	Gal1α	iPrOH	$C_3F_6S_2O_4NH$	DCM	36.0	32.6	67.4	7α, 7β
299	10	Gal1α	iPrOH	$C_3F_6S_2O_4NH$	DCM	45.0	41.0	59.0	7α, 7β
300	30	Gal1α	iPrOH	$C_3F_6S_2O_4NH$	DCM	59.6	47.1	52.9	7α, 7β

Table S9: Validation data collected from the automated flow platform to predict $C_3F_6S_2O_4NH$ with Gal1α.

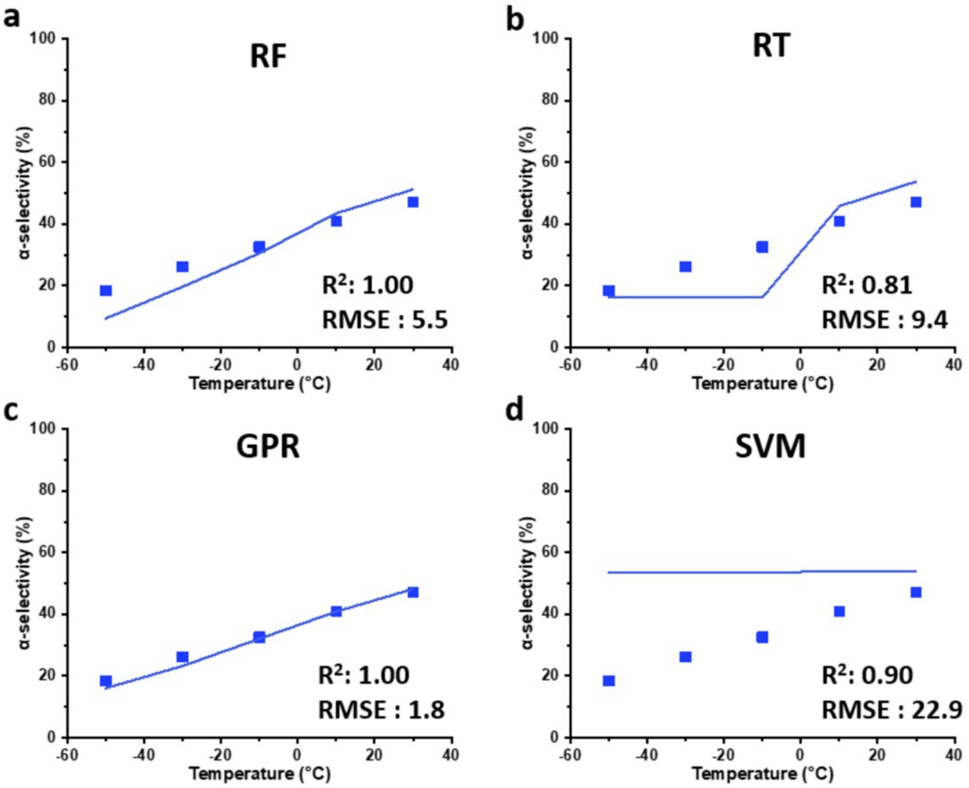

Temp. (°C)	Experimental	RF	RT	GPR	SVM
-50	18.5	9.5	16.2	16.1	53.6
-30	26.2	19.7	16.2	23.3	53.5
-10	32.6	30.6	16.2	32.1	53.7
10	41	43.5	45.8	40.9	53.8
30	47.1	51.3	53.8	48.2	54.0

Figure S12: **a**, Prediction with Random Forest (RF). **b**, Prediction with Regression Tree (RT). **c**, Prediction with Gaussian Process Regression (GPR). **d**, Prediction with Support Vector Machine (SVR). experimental (data points); predicted (solid colored line).

Prediction of Activator- *4,4,5,5,6,6-hexafluoro-1,3,2-dithiazinane 1,1,3,3-tetraoxide with Mannose Electrophile*

The experimental results for the out-of-sample glycosylations involving super acid 4,4,5,5,6,6-hexafluoro-1,3,2-dithiazinane 1,1,3,3-tetraoxide as activator coupling a mannose imidate electrophile and *tert*-butanol as nucleophile are given in Table S10. The accuracy of the random forest algorithm is depicted in Figure S13 and compared to three other algorithms: regression tree, Gaussian process regression, and support vector machine.

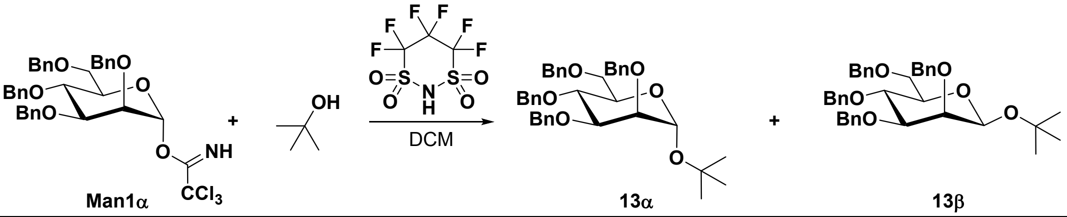

Entry	Temp. (°C)	Donor	Acceptor	Activator	Solvent	Yield (%)	α ratio (%)	β ratio (%)	Product
301	-50	Man1α	tBuOH	$C_3F_6S_2O_4NH$	DCM	71.9	36.7	63.3	13α, 13β
302	-30	Man1α	tBuOH	$C_3F_6S_2O_4NH$	DCM	69.6	42.8	57.2	13α, 13β
303	-10	Man1α	tBuOH	$C_3F_6S_2O_4NH$	DCM	86.3	48.2	51.8	13α, 13β
304	10	Man1α	tBuOH	$C_3F_6S_2O_4NH$	DCM	87.5	64.4	35.6	13α, 13β
305	30	Man1α	tBuOH	$C_3F_6S_2O_4NH$	DCM	91.7	98	2	13α, 13β

Table S10: Validation data collected from the automated flow platform to predict $C_3F_6S_2O_4NH$ with Man1α.

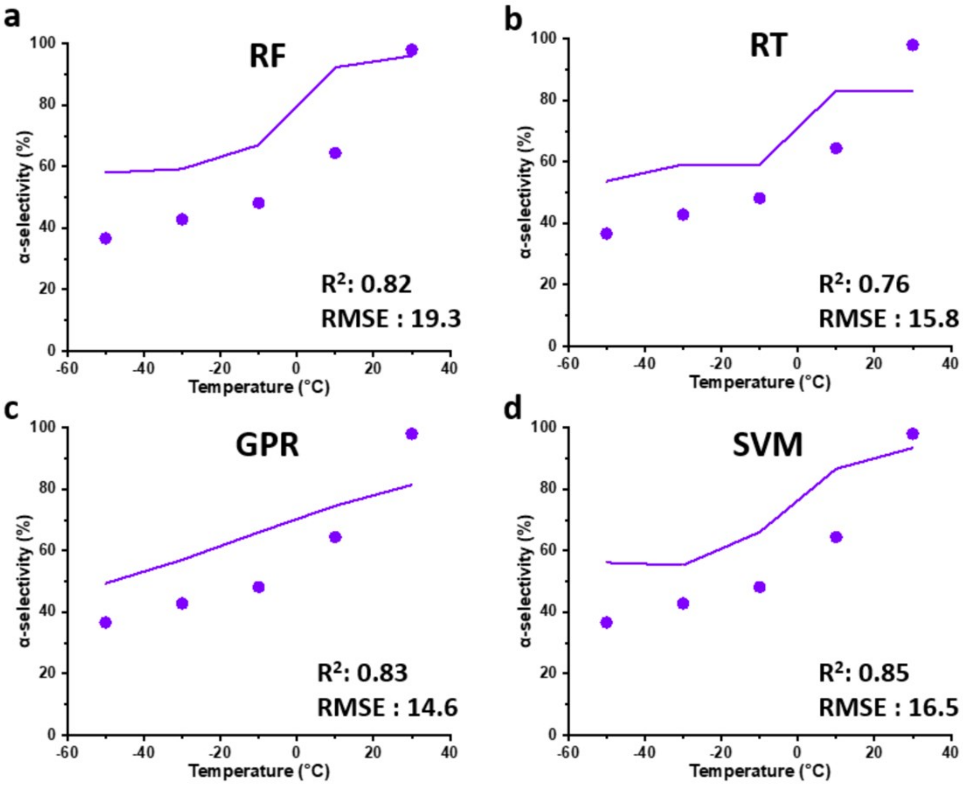

Temp. (°C)	Experimental	RF	RT	GPR	SVM
-50	36.7	58.0	53.7	49.4	56.1
-30	42.8	59.2	59.1	57.0	55.3
-10	48.2	67.0	59.1	66.0	66.0
10	64.4	92.2	83.1	74.6	86.6
30	98	96.0	83.1	81.4	93.4

Figure S13: **a**, Prediction with Random Forest (RF). **b**, Prediction with Regression Tree (RT). **c**, Prediction with Gaussian Process Regression (GPR). **d**, Prediction with Support Vector Machine (SVR). experimental (data points); predicted (solid colored line).

Prediction of Solvent- **α,α,α-trifluorotoluene**

The experimental results for the out-of-sample glycosylations involving α,α,α-trifluorotoluene as solvent coupling a glucose imidate electrophile and isopropanol as nucleophile are given in

Table S11. The accuracy of the random forest algorithm is depicted in Figure S14 and compared to three other algorithms: regression tree, Gaussian process regression, and support vector machine.

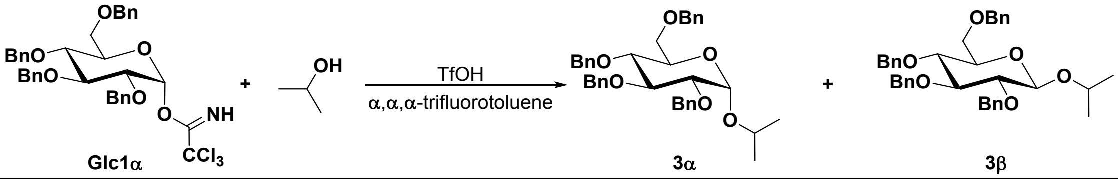

Entry	Temp. (°C)	Donor	Acceptor	Activator	Solvent	Yield (%)	α ratio (%)	β ratio (%)	Product
306	-20	Glc1α	iPrOH	TfOH	3F-Toluene	85.5	31.7	68.3	3α, 3β
307	10	Glc1α	iPrOH	TfOH	3F-Toluene	96.8	51.8	48.2	3α, 3β
308	30	Glc1α	iPrOH	TfOH	3F-Toluene	97.5	60.3	39.7	3α, 3β
309	50	Glc1α	iPrOH	TfOH	3F-Toluene	85.5	61.9	38.1	3α, 3β
310	70	Glc1α	iPrOH	TfOH	3F-Toluene	82.2	64.4	35.6	3α, 3β
311	90	Glc1α	iPrOH	TfOH	3F-Toluene	60.2	63.3	36.7	3α, 3β

Table S11: Validation data collected from the automated flow platform to predict α,α,α-trifluorotoluene.

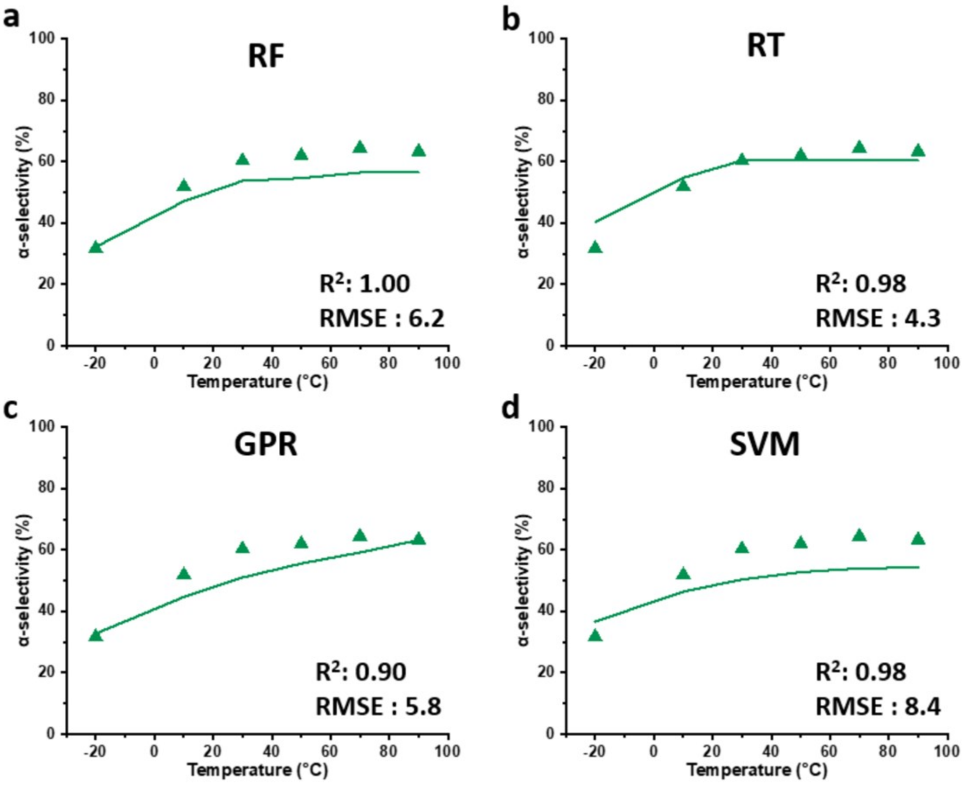

Temp. (°C)	Experimental	RF	RT	GPR	SVM
-20	31.7	32.3	40.4	32.8	36.7
10	51.8	47.1	54.7	44.7	46.4
30	60.3	53.7	60.3	51.0	50.4
50	61.9	54.6	60.3	55.6	52.8
70	64.4	56.5	60.3	59.2	54.0
90	63.3	56.5	60.3	63.2	54.3

Figure S14: **a**, Prediction with Random Forest (RF). **b**, Prediction with Regression Tree (RT). **c**, Prediction with Gaussian Process Regression (GPR). **d**, Prediction with Support Vector Machine (SVR). experimental (data points); predicted (solid colored line).

Prediction of Solvent-**1,4-dioxane**

The experimental results for the out-of-sample glycosylations involving 1,4-dioxane as solvent coupling a galactose imidate electrophile and isopropanol as nucleophile are given in Table S12. The accuracy of the random forest algorithm is depicted in Figure S15 and compared to three other algorithms: regression tree, Gaussian process regression, and support vector machine.

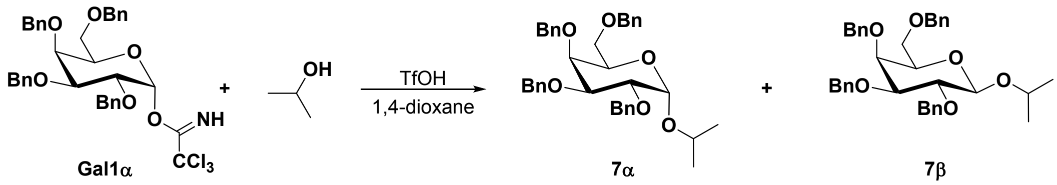

Entry	Temp. (°C)	Donor	Acceptor	Activator	Solvent	Yield (%)	α ratio (%)	β ratio (%)	Product
312	20	Gal1α	iPrOH	TfOH	1,4-Dioxane	99.2	67.9	32.1	7α, 7β
313	40	Gal1α	iPrOH	TfOH	1,4-Dioxane	96.0	71.3	28.7	7α, 7β
314	60	Gal1α	iPrOH	TfOH	1,4-Dioxane	89.6	73.1	26.9	7α, 7β
315	80	Gal1α	iPrOH	TfOH	1,4-Dioxane	82.3	73.9	26.1	7α, 7β
316	100	Gal1α	iPrOH	TfOH	1,4-Dioxane	80.3	74.2	25.8	7α, 7β

Table S12: Validation data collected from the automated flow platform to predict 1,4-dioxane.

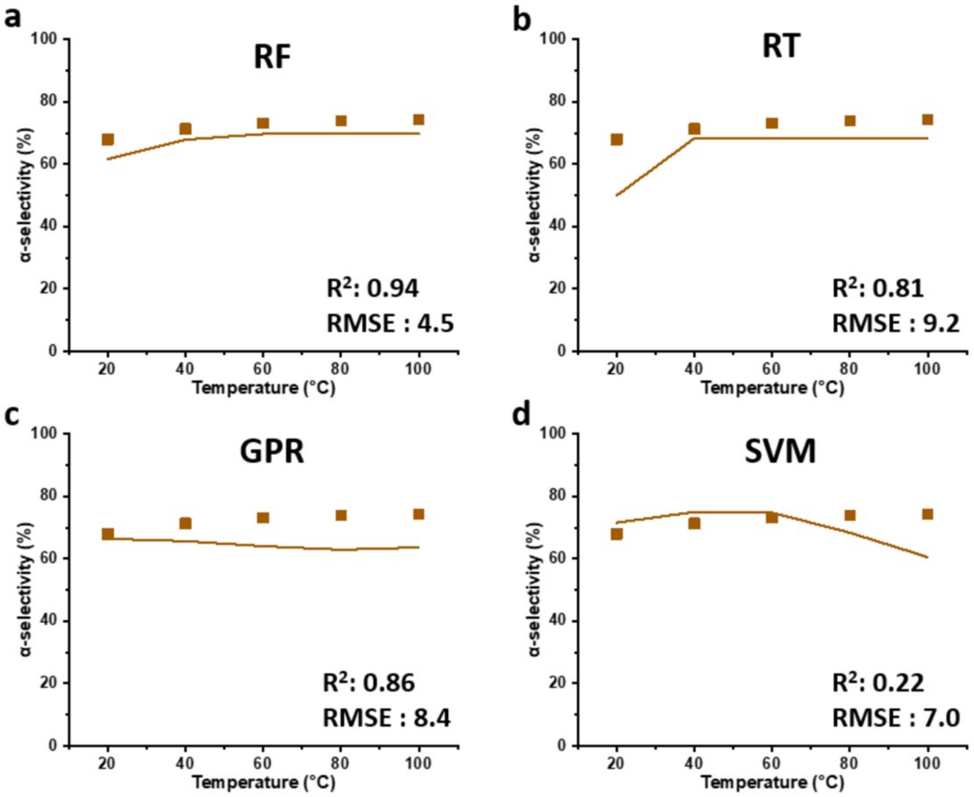

Temp. (°C)	Experimental	RF	RT	GPR	SVM
20	67.9	61.6	49.9	66.4	71.6
40	71.3	67.9	68.3	65.7	75.0
60	73.1	69.7	68.3	64.0	74.7
80	73.9	69.7	68.3	62.9	68.3
100	74.2	69.7	68.3	63.6	60.5

Figure S15: **a**, Prediction with Random Forest (RF). **b**, Prediction with Regression Tree (RT). **c**, Prediction with Gaussian Process Regression (GPR). **d**, Prediction with Support Vector Machine (SVR). experimental (data points); predicted (solid colored line).

Prediction of leaving group stereochemistry

β-glucose electrophile with ethanol in toluene

The experimental results for the out-of-sample glycosylations involving a glucose imidate electrophile with the leaving group as the beta anomer with ethanol as nucleophile are given in Table S13. The accuracy of the random forest algorithm is depicted in Figure S16 and compared to three other algorithms: regression tree, Gaussian process regression, and support vector machine.

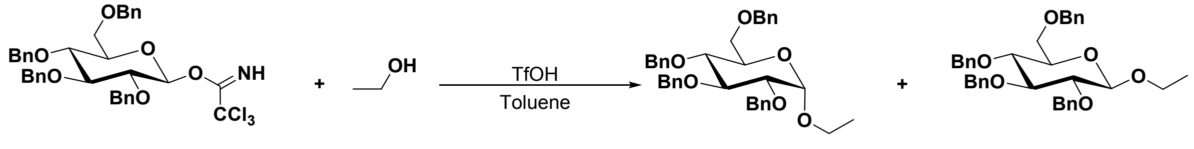

Entry	Temp. (°C)	Donor	Acceptor	Activator	Solvent	Yield (%)	α ratio (%)	β ratio (%)	Product
317	-50	Glc1β	EtOH	TfOH	Toluene	83.2	31.4	68.6	2α, 2β
318	-30	Glc1β	EtOH	TfOH	Toluene	86.6	37.1	62.9	2α, 2β
319	-10	Glc1β	EtOH	TfOH	Toluene	85.5	46.7	53.3	2α, 2β
320	10	Glc1β	EtOH	TfOH	Toluene	82.7	54.7	45.3	2α, 2β
321	30	Glc1β	EtOH	TfOH	Toluene	78.2	60.8	39.2	2α, 2β
322	50	Glc1β	EtOH	TfOH	Toluene	76.0	60.6	39.4	2α, 2β
323	70	Glc1β	EtOH	TfOH	Toluene	73.8	63.2	36.8	2α, 2β

Table S13: Validation data collected from the automated flow platform to predict β-glucose electrophile with ethanol in toluene.

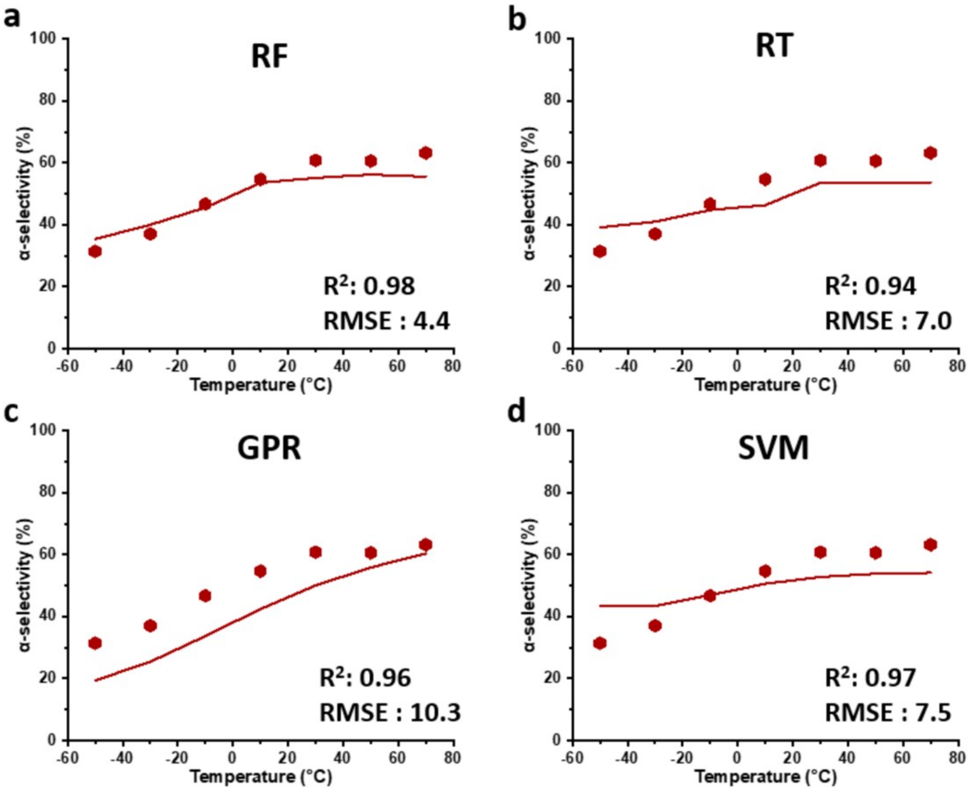

Temp. (°C)	Experimental	RF	RT	GPR	SVM
-50	31.4	35.4	39.2	19.4	43.5
-30	37.1	40.0	41.0	25.4	43.4
-10	46.7	45.6	44.8	33.7	47.0
10	54.7	53.6	46.4	42.5	50.6
30	60.8	55.2	53.6	50.1	52.8
50	60.6	56.2	53.6	55.9	53.9
70	63.2	55.5	53.6	60.4	54.2

Figure S16: **a**, Prediction with Random Forest (RF). **b**, Prediction with Regression Tree (RT). **c**, Prediction with Gaussian Process Regression (GPR). **d**, Prediction with Support Vector Machine (SVR). experimental (data points); predicted (solid colored line).

β-glucose electrophile with *tert*-butanol in toluene

The experimental results for the out-of-sample glycosylations involving a glucose imidate electrophile with the leaving group as the beta anomer with tert-butanol as nucleophile are given in Table S14. The accuracy of the random forest algorithm is depicted in Figure S17 and compared to three other algorithms: regression tree, Gaussian process regression, and support vector machine.

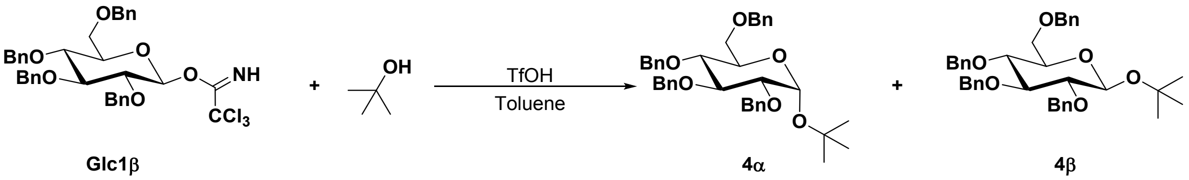

Entry	Temp. (°C)	Donor	Acceptor	Activator	Solvent	Yield (%)	α ratio (%)	β ratio (%)	Product
324	-50	Glc1β	tBuOH	TfOH	Toluene	68.6	79.6	20.4	4α, 4β
325	-30	Glc1β	tBuOH	TfOH	Toluene	89.1	78.6	21.4	4α, 4β
326	-10	Glc1β	tBuOH	TfOH	Toluene	99.1	76.6	23.4	4α, 4β
327	10	Glc1β	tBuOH	TfOH	Toluene	95.0	74.1	25.9	4α, 4β
328	30	Glc1β	tBuOH	TfOH	Toluene	90.1	71.2	28.8	4α, 4β
329	50	Glc1β	tBuOH	TfOH	Toluene	84.6	68.5	31.5	4α, 4β
330	70	Glc1β	tBuOH	TfOH	Toluene	74.3	65.1	34.9	4α, 4β

Table S14: Validation data collected from the automated flow platform to predict β-glucose electrophile with *tert*-butanol in toluene.

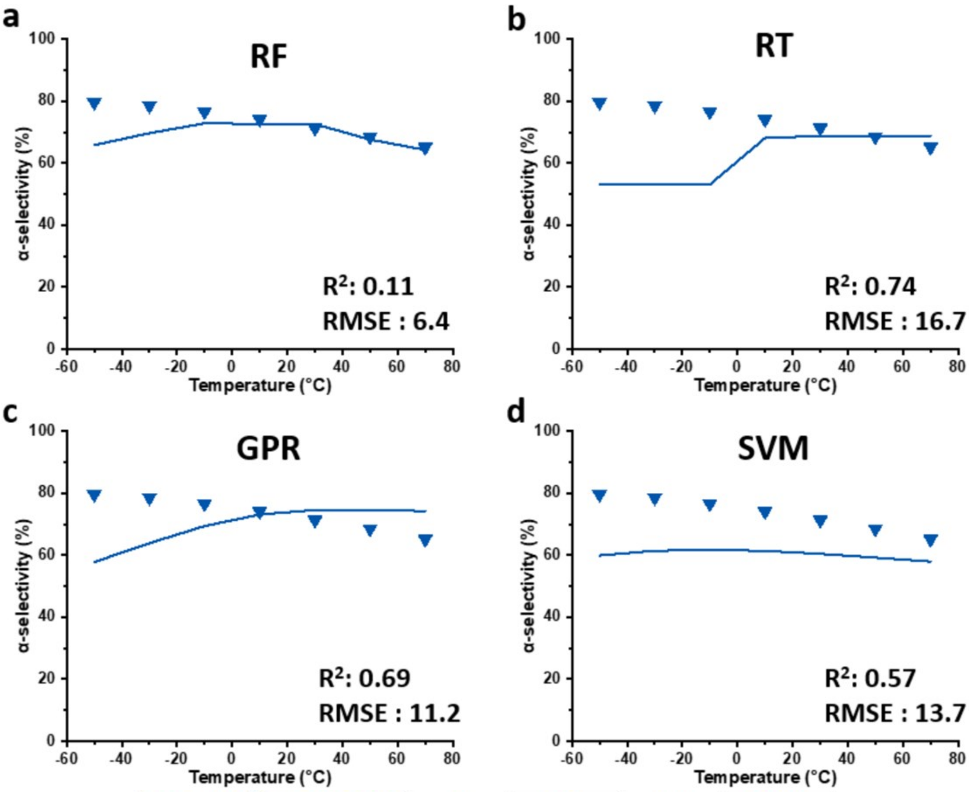

Temp. (°C)	Experimental	RF	RT	GPR	SVM
-50	79.6	65.8	53.1	57.8	59.9
-30	78.6	69.7	53.1	63.9	61.4
-10	76.6	72.8	53.1	69.4	61.7
10	74.1	72.6	68.1	73.1	61.4
30	71.2	72.6	68.8	74.6	60.5
50	68.5	67.6	68.8	74.5	59.2
70	65.1	64.3	68.8	74.3	57.9

Figure S17: **a**, Prediction with Random Forest (RF). **b**, Prediction with Regression Tree (RT). **c**, Prediction with Gaussian Process Regression (GPR). **d**, Prediction with Support Vector Machine (SVR). experimental (data points); predicted (solid colored line).

β-galactose electrophile with isopropanol in DCM

The experimental results for the out-of-sample glycosylations involving a galactose imidate electrophile with the leaving group as the beta anomer with isopropanol as nucleophile in DCM are given in Table S15. The accuracy of the random forest algorithm is depicted in Figure S18 and compared to three other algorithms: regression tree, Gaussian process regression, and support vector machine.

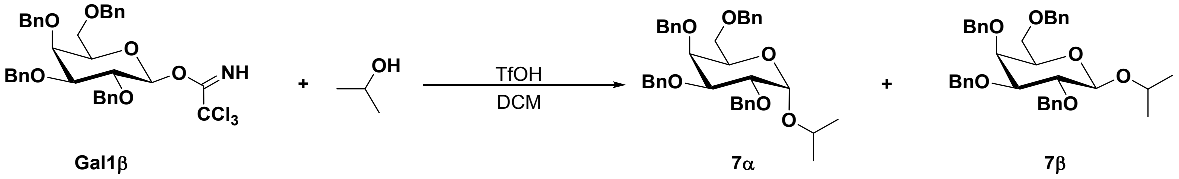

Entry	Temp. (°C)	Donor	Acceptor	Activator	Solvent	Yield (%)	α ratio (%)	β ratio (%)	Product
331	-50	Gal1β	iPrOH	TfOH	DCM	99.4	23.4	76.6	7α, 7β
332	-30	Gal1β	iPrOH	TfOH	DCM	99.9	32.4	67.6	7α, 7β
333	-10	Gal1β	iPrOH	TfOH	DCM	93.8	39.7	60.3	7α, 7β
334	10	Gal1β	iPrOH	TfOH	DCM	81.6	47.2	52.8	7α, 7β
335	30	Gal1β	iPrOH	TfOH	DCM	75.6	55.0	45.0	7α, 7β

Table S15: Validation data collected from the automated flow platform to predict β-galactose electrophile with isopropanol in DCM.

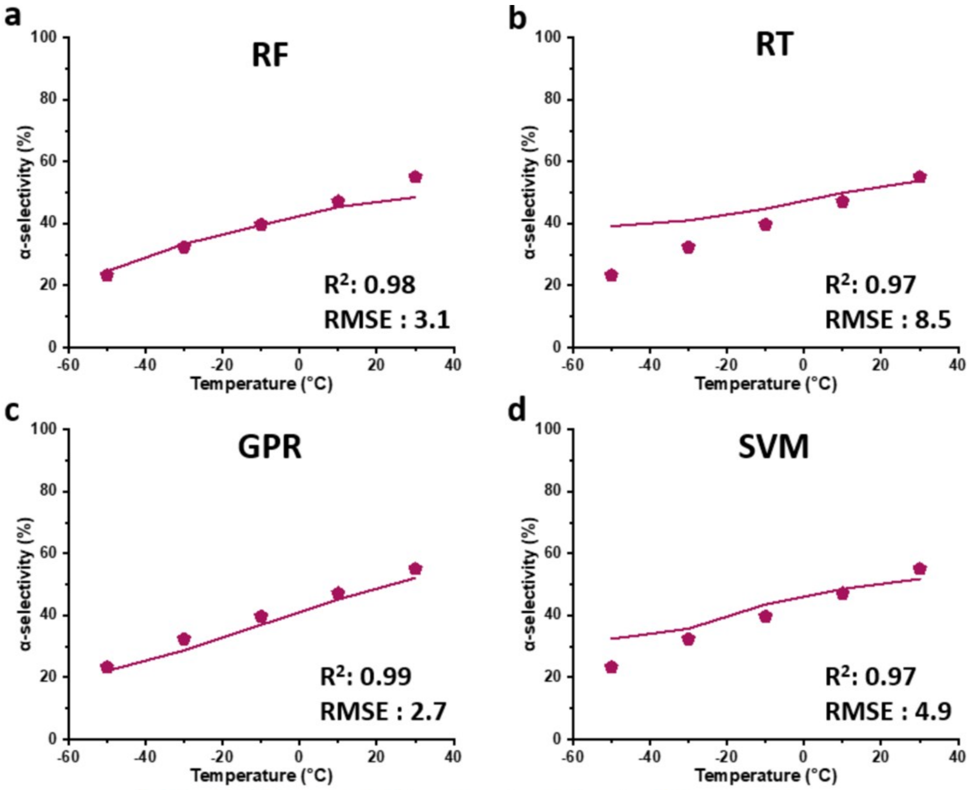

Temp. (°C)	Experimental	RF	RT	GPR	SVM
-50	23.4	24.6	39.2	22.1	32.5
-30	32.4	33.4	41.0	28.8	35.7
-10	39.7	39.5	44.8	37.0	43.6
10	47.2	45.4	49.9	45.2	48.6
30	55.0	48.5	53.8	52.1	51.8

Figure S18: **a**, Prediction with Random Forest (RF). **b**, Prediction with Regression Tree (RT). **c**, Prediction with Gaussian Process Regression (GPR). **d**, Prediction with Support Vector Machine (SVR). experimental (data points); predicted (solid colored line).

β-galactose electrophile with isopropanol in toluene

The experimental results for the out-of-sample glycosylations involving a galactose imidate electrophile with the leaving group as the beta anomer with isopropanol as nucleophile in toluene are given in Table S16. The accuracy of the random forest algorithm is depicted in Figure S19 and compared to three other algorithms: regression tree, Gaussian process regression, and support vector machine.

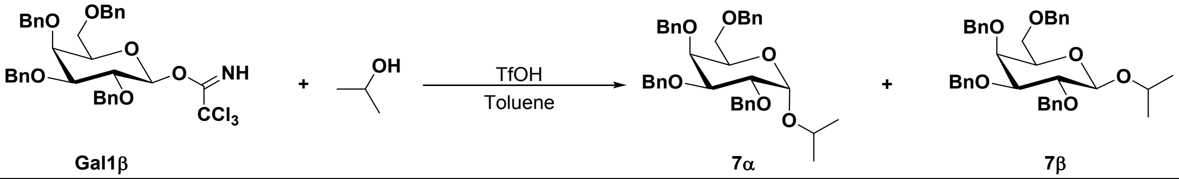

Entry	Temp. (°C)	Donor	Acceptor	Activator	Solvent	Yield (%)	α ratio (%)	β ratio (%)	Product
336	-50	Gal1β	iPrOH	TfOH	Toluene	80.6	33.0	67.0	7α, 7β
337	-30	Gal1β	iPrOH	TfOH	Toluene	96.4	34.3	65.7	7α, 7β
338	-10	Gal1β	iPrOH	TfOH	Toluene	96.8	39.9	60.1	7α, 7β
339	10	Gal1β	iPrOH	TfOH	Toluene	93.2	47.3	52.7	7α, 7β
340	30	Gal1β	iPrOH	TfOH	Toluene	94.1	56.3	43.7	7α, 7β
341	50	Gal1β	iPrOH	TfOH	Toluene	90.8	62.7	37.3	7α, 7β
342	70	Gal1β	iPrOH	TfOH	Toluene	87.3	68.6	31.4	7α, 7β

Table S16: Validation data collected from the automated flow platform to predict β-galactose electrophile with isopropanol in toluene.

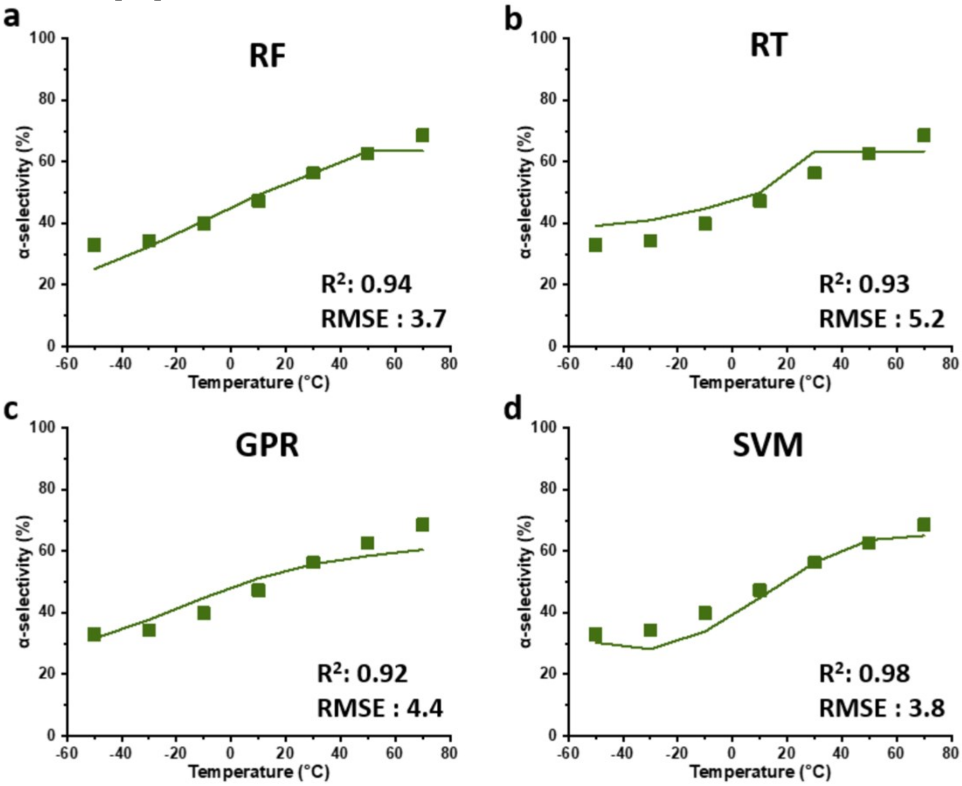

Temp. (°C)	Experimental	RF	RT	GPR	SVM
-50	33.0	25.2	39.2	31.6	30.3
-30	34.3	32.4	41.0	37.8	28.2
-10	39.9	40.8	44.8	44.8	34.0
10	47.3	49.3	49.9	51.2	44.9
30	56.3	56.2	63.3	55.7	56.3
50	62.7	63.4	63.3	58.4	63.6
70	68.6	63.5	63.3	60.5	65.0

Figure S19: **a**, Prediction with Random Forest (RF). **b**, Prediction with Regression Tree (RT). **c**, Prediction with Gaussian Process Regression (GPR). **d**, Prediction with Support Vector Machine (SVR). experimental (data points); predicted (solid colored line).

General experimental details for preparing building blocks

Commercial grade solvents and reagents were used unless stated otherwise. Anhydrous solvents were obtained from a dry solvent system (Waters, Milford, USA). Unless otherwise noted, all other reagents and solvents were purchased from commercial suppliers and used without further purification. All reactions were carried out under an argon atmosphere. Analytical thin layer chromatography (TLC) was performed on Macherey-Nagel Pre-coated TLC-sheets, ALUGRAM Xtra SIL G/UV_{254} sheets and visualized with 254 nm light, 2,5-dinitrophenylhydrazine (DNPH) staining solutions followed by heating. Purification of the reaction products was carried out by flash chromatography using Macherey-Nagel Silica 60 M (0.04-0.063 mm) silica gel. Proton (^{1}H) NMR spectra were recorded using Agilent 400 (400 MHz) or Agilent 600 (600 MHz) in $CDCl_3$ and are reported in ppm relative to the residual solvent peaks ($CDCl_3$ at 7.26 ppm) Peaks are reported as: s = singlet, d = doublet, t = triplet, q = quartet, quint = quintet, m = multiplet. Carbon (^{13}C) NMR spectra were recorded with ^{1}H-decoupling on Agilent 400 (101 MHz) or Agilent 600 (151 MHz) in $CDCl_3$ and reported in ppm relative to the residual solvent peak ($CDCl_3$ at 77.16 ppm).

Procedure for drying solvents

Toluene, tert-butylbenzene, anisole, α,α,α-trifluorotoluene, dichloromethane, chloroform, acetonitrile, methyl tert-butyl ether, and 1,4-dioxane were dried using 3 Å molecular sieves. Molecular sieves were activated by heating under microware radiation of 500 W for nine minutes and subsequent cooling to ambient temperature under high vacuum. This procedure was repeated five times. The activated molecular sieves were added to the solvents and the solvents were kept under argon atmosphere for two days. The water content of the solvents was determined using Karl Fischer titration.

Calculation of descriptor

Structures were optimized and DFT calculations performed using Spartan '18, Version 1.4.0. The following compounds were calculated at the B3LYP 6-31G* level of theory: electrophile- Glc1α, Glc1β, Glc2β, Glc3α, Gal1α, Gal1β, Gal2β, Gal3α, Man1α, Man2α, Man3α, Fuc1α. The following compounds were calculated at the B3LYP 6-311G* level of theory: nucleophile- MeOH, EtOH, iPrOH, tBuOH, 2F-EtOH, 3F-EtOH, GlcOH, ManOH, conjugate base of acid catalyst- $FSO3^-$, MsO^-, TfO^-, $Tf2N^-$, $C_3F_6S_2O_4N^-$, solvent- toluene, tert-butylbenzene, anisole, α,α,α-trifluorotoluene, dichloromethane, chloroform, acetonitrile, methyl tert-butyl ether, and 1,4-dioxane.

Analysis Section

The reactions were monitored using HPLC. The HPLC system used was a Kanuer Platin Blue system, equipped with a UV detector (254 nm). The column used was Macherey-Nagel Nucleosil 100-5 OH diol column with particle size of 5 µm, I.D. of 4.6 mm and length of 250 mm. The column was housed inside a column oven, and was maintained at 20 °C for all analysis. The mobile phase was a gradient mixture of HPLC grade ethyl acetate and hexane at a constant flowrate of 1 mL/min.

HPLC Method A

Time [min]	Flow [ml/min]	EtOAc [%]	Hexane [%]
0	1	2	98
14	1	25	75
16	1	70	30
18	1	70	30
19	1	2	98
22	1	2	98

HPLC Method B

Time [min]	Flow [ml/min]	EtOAc [%]	Hexane [%]
0	1	2	98
5	1	30	70
10	1	30	70
16	1	70	30
18	1	70	30
19	1	30	70
20	1	10	90

Preparation and NMR data are available in ref 1.

Electrophile – Glc1α, Glc1β, Glc2β, Glc3α, Gal1α, Gal2β, Gal3α, Man1α, Man2α, Man3α

Product – 1α, 1β, 2α, 2β, 3α, 3β, 4α, 4β, 5α, 5β, 6α, 6β, 7α, 7β, 8α, 8β, 9α, 9β, 10α, 10β, 11α, 11β, 12α, 12β, 13α, 13β, 14α

Preparation of 2,3,4-tri-O-benzyl-α-L-fucopyranosyl trichloroacetimidate (**Fuc1α**)[2]

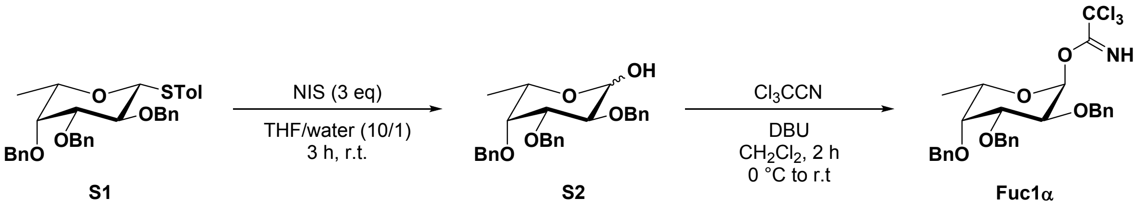

To a solution **S1**[3] (279 mg, 0.52 mmol) in THF (20 mL) and water (2 mL) were added N-iodosuccinimide (232 mg 1.03 mmol), and then stirred for 1 h at room temperature. The reaction mixture was quenched with sat. aq. $NaHCO_3$ solution (10 mL) and DCM (10 mL). The organic layer was extracted with DCM (2 × 10 mL) and washed with brine (10 mL). The organic layer was dried over anhydrous Na_2SO_4, filtered and evaporated under reduced pressure for column chromatography purification (Elution: n-hexane/EtOAc = 6/1 to 2/1) and obtained as an inseparable α/β mixture **S3** (Rf: 0.1 in *n*-Hexane/ EtOAc = 3/1). To compound **S2** in dry DCM (15 mL) were added CCl_3CN (0.2 mL, 1.99 mmol) and DBU (0.05 mL, 0.33 mmol) at 0 °C. The dark solution was stirred at room temperature for 2 h, and then the reaction mixture was concentrated. The residue was purified by silica gel column chromatography (Elution: Toluene/EtOAc = 20/1 containing 1% Et_3N) to give **Fuc1α** (153 mg, 0.26 mmol) as a white solid (Rf: 0.21 in Toluene/EtOAc = 3/1) with 51% yield; ^1H NMR (400 MHz, Chloroform-*d*) δ 8.50 (s, 1H), 7.53 – 7.11 (m, 15H), 6.52 (d, J = 3.5 Hz, 1H), 5.01 (d, J = 11.5 Hz, 1H), 4.95 – 4.61 (m, 5H), 4.24 (dd, J = 10.2, 3.4 Hz, 1H), 4.09 (d, J = 6.6 Hz, 1H), 4.06 – 3.96 (m, 1H), 3.71 (s, 1H), 1.16 (d, J = 6.5 Hz, 3H).

This data is in accordance with those previously published.[4]

Preparation of 2,3,4,6-tetra-O-benzyl-β-D-galactopyranosyl trichloroacetimidate (**Glc1β**)

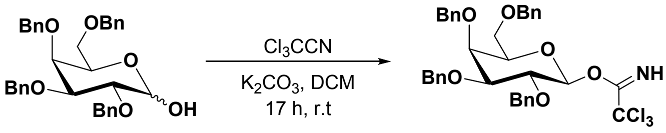

S3 **Gal1β**

To compound **S3**[1] in dry DCM (10 mL) were added CCl_3CN (0.1 mL, 1 mmol) and K_2CO_3 (138 mg, 1 mmol) at room temperature. The solution was stirred for 17 h at room temperature, and then the reaction mixture was concentrated. The residue was purified by silica gel column chromatography (Elution: n-hexane/EtOAc = 10/1 containing 1% Et_3N) to give **Gal1β** (67.1 mg, 0.11 mmol) as white solid (Rf: 0.49 in *n*-Hexane/ EtOAc = 3/1). with 57% yield; ^{1}H NMR (600 MHz, Chloroform-*d*) δ 8.62 (s, 1H), 7.36 – 7.26 (m, 20H), 5.75 (d, H-1β, *J* = 8.0 Hz, 1H), 4.95 (d, *J* = 11.5 Hz, 1H), 4.91 (d, *J* = 10.8 Hz, 1H), 4.81 (d, *J* = 10.8 Hz, 1H), 4.73 (d, *J* = 2.0 Hz, 2H), 4.64 (d, *J* = 11.5 Hz, 1H), 4.47 (d, *J* = 11.7 Hz, 1H), 4.43 (d, *J* = 11.8 Hz, 1H), 4.09 (dd, *J* = 9.7, 8.0 Hz, 1H), 3.99 (d, *J* = 3.1 Hz, 1H), 3.75 (t, *J* = 6.5 Hz, 1H), 3.68 – 3.59 (m, 3H). This data is in accordance with those previously published.[5]

Methyl 2,3,4-tri-O-benzyl-α-D-mannopyranoside (**ManOH**)

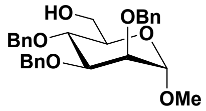

ManOH

ManOH was synthesized as described in ref 7. ^{1}H NMR (400 MHz, Chloroform-*d*) δ 7.35 – 7.25 (m, 15H), 4.93 (d, *J* = 10.9 Hz, 1H), 4.77 (d, *J* = 12.3 Hz, 1H), 4.71 – 4.68 (m, 2H), 4.66 (d, *J* = 6.0 Hz, 1H), 4.62 (s, 2H), 3.95 (t, *J* = 9.4 Hz, 1H), 3.89 (dd, *J* = 9.4, 2.9 Hz, 1H), 3.84 (dd, *J* = 11.7, 2.9 Hz, 1H), 3.80 – 3.72 (m, 2H), 3.61 (ddd, *J* = 9.4, 4.7, 3.0 Hz, 1H), 3.29 (s, 3H), 1.79 (bs, 1H). This data is in accordance with those previously published.[6]

Methyl 2,3,4-tri-O-benzyl-α-D-glucopyranoside (**GlcOH**)

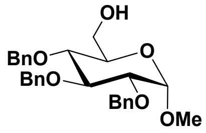

GlcOH

GlcOH was synthesized as described in ref 7. ^{1}H NMR (400 MHz, Chloroform-*d*) δ 7.40 – 7.25 (m, 15H), 4.99 (d, *J* = 10.9 Hz, 1H), 4.92 – 4.77 (m, 3H), 4.65 (dd, *J* = 11.6, 9.5 Hz, 2H), 4.57 (d, *J* = 3.6 Hz, 1H), 4.01 (t, *J* = 9.3 Hz, 1H), 3.77 (d, *J* = 11.4 Hz, 1H), 3.73 – 3.62 (m, 2H), 3.56 – 3.47 (m, 2H), 3.37 (s, 3H), 1.60 (s, 1H). This data is in accordance with those previously published.[6]

Isopropyl 2,3,4-tri-O-benzyl-α-L-fucopyranoside (**15αβ**)

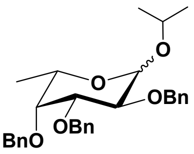

15αβ

^{1}H NMR (600 MHz, Chloroform-*d*) δ 7.52 – 7.11 (m, 57H), 5.01 – 4.94 (m, 7H), 4.90 (d, H-1α, $J_{1,2}$ = 3.8 Hz, 1H), 4.88 (d, *J* = 11.7 Hz, 1H), 4.80 (dd, *J* = 11.9, 2.8 Hz, 4H), 4.77 – 4.63 (m, 13H), 4.38 (d, H-1β, $J_{1,2}$ = 7.7 Hz, 3H), 4.03 – 3.91 (m, 6H), 3.87 (hept, *J* = 6.2 Hz, 1H), 3.78 (dd, *J* = 9.7, 7.7 Hz, 3H), 3.67 (dd, *J* = 2.9, 1.2 Hz, 1H), 3.54 (dd, *J* = 3.1, 1.1 Hz, 3H), 3.50 (dd, *J* = 9.7, 3.0 Hz, 3H), 3.43 (q, *J* = 6.5, 1.1 Hz, 3H), 1.27 (d, Me-β, *J* = 6.2 Hz, 9H), 1.21 (d, Me-β, *J* = 6.1, 9H), 1.21 (d, Me-α, *J* = 6.2, 3H), 1.18 (d, Me-α, *J* = 6.1 Hz, 3H), 1.16 (d, Me-β, *J* = 6.4 Hz, 9H), 1.09 (d, Me-α, *J* = 6.6 Hz, 3H). This data is in accordance with those previously published.[7] The HPLC trace is provided in Figure S20.

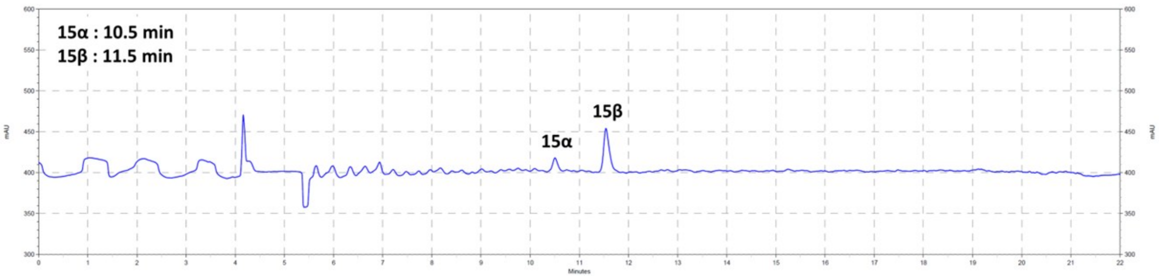

Figure S20: a HPLC spectrum of **15α**, **15β** (Method A)

Methyl 2,3,4,6-tetra-O-benzyl-α/β-D-galactopyranosyl-(1→6)-2,3,4-tri-O-benzyl-α-D-mannopyranoside (**16αβ**)

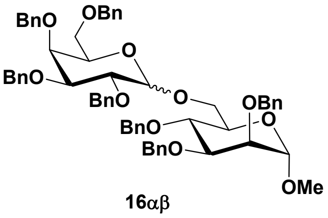

16αβ

α-isomer: ^{1}H NMR (600 MHz, Chloroform-*d*) δ 7.50 – 7.08 (m, 35H), 5.11 (d, *J* = 3.5 Hz, 1H), 4.96 (d, *J* = 11.5 Hz, 1H), 4.87 (d, *J* = 11.0 Hz, 1H), 4.80 (d, *J* = 11.9 Hz, 1H), 4.74 – 4.65 (m, 6H), 4.63 – 4.57 (m, 4H), 4.46 (d, *J* = 11.8 Hz, 1H), 4.40 (d, *J* = 11.8 Hz, 1H), 4.09 – 4.03 (m, 2H), 3.99 – 3.95 (m, 2H), 3.94 (d, *J* = 9.6 Hz, 1H), 3.89 (dd, *J* = 9.3, 3.1 Hz, 1H), 3.85 (d, *J* = 4.0 Hz, 2H), 3.80 – 3.76 (m, 2H), 3.59 (dd, *J* = 9.3, 7.4 Hz, 1H), 3.54 (dd, *J* = 9.3, 5.7 Hz, 1H), 3.22 (s, 3H);

β-isomer: ^{1}H NMR (600 MHz, Chloroform-*d*) δ 7.29 (m, 35H), 5.00 (d, *J* = 10.8 Hz, 1H), 4.94 (d, *J* = 11.6 Hz, 1H), 4.81 – 4.74 (m, 2H), 4.74 – 4.52 (m, 8H), 4.49 (d, *J* = 11.2 Hz, 1H), 4.41 (dt, *J* = 22.2, 10.2 Hz, 3H), 4.23 (d, *J* = 10.6 Hz, 1H), 3.93 – 3.75 (m, 6H), 3.70 (dd, *J* = 10.7, 6.3 Hz, 1H), 3.59 (dt, *J* = 24.2, 8.6 Hz,i 2H), 3.54 – 3.46 (m, 2H), 3.21 (s, 3H). This data is in accordance with those previously published.[8] HPLC trace of these anomers is provided in Figure S21.

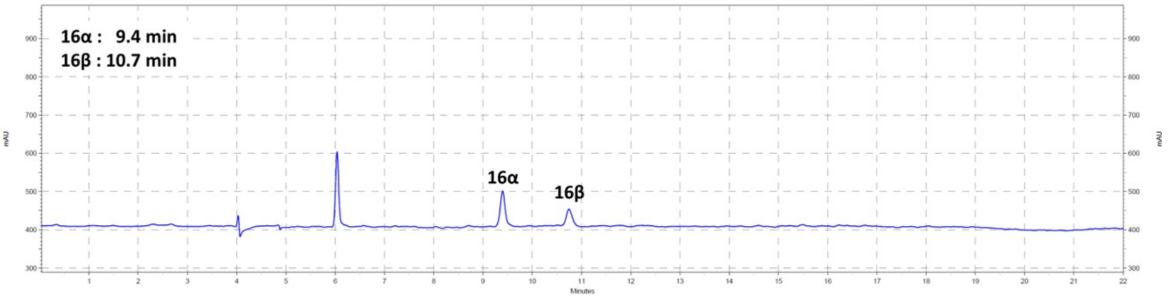

Figure S21: a HPLC spectrum of **16α**, **16β** (Method B)

Methyl 2,3,4,6-tetra-O-benzyl-α/β-D-galactopyranosyl-(1→6)-2,3,4-tri-O-benzyl-α-D-glucopyranoside (**17αβ**)

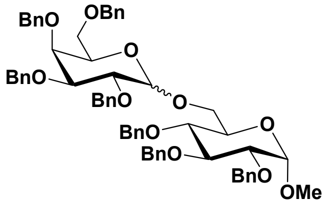

17αβ

α-isomer: ^{1}H NMR (400 MHz, Chloroform-*d*) δ 7.41 – 7.11 (m, 35H), 4.99 (s, 1H), 4.94 (t, *J* = 9.3 Hz, 2H), 4.84 (d, *J* = 10.9 Hz, 1H), 4.82 – 4.76 (m, 2H), 4.76 – 4.65 (m, 4H), 4.61 – 4.55 (m, 2H), 4.55 – 4.49 (m, 2H), 4.43 (d, *J* = 11.8 Hz, 1H), 4.36 (d, *J* = 11.9 Hz, 1H), 4.03 (d, *J* = 9.8 Hz, 1H), 3.99 – 3.87 (m, 4H), 3.83 – 3.74 (m, 2H), 3.72 (d, *J* = 12.3 Hz, 1H), 3.58 (t, *J* = 9.4 Hz, 1H), 3.54 – 3.46 (m, 2H), 3.41 (d, *J* = 8.8 Hz, 1H), 3.29 (s, 3H);

β-isomer: ^{1}H NMR (400 MHz, Chloroform-*d*) δ 7.41 – 7.09 (m, 35H), 5.01 – 4.87 (m, 3H), 4.86 – 4.33 (m, 12H), 4.30 (d, *J* = 7.7 Hz, 1H), 4.13 (d, *J* = 10.8 Hz, 1H), 3.97 (t, *J* = 9.2 Hz, 1H), 3.92 – 3.73 (m, 3H), 3.65 – 3.42 (m, 7H), 3.29 (s, 3H). This data is in accordance with those previously published.[9,10] The HPLC trace of these two anomers is provided in Figure S22.

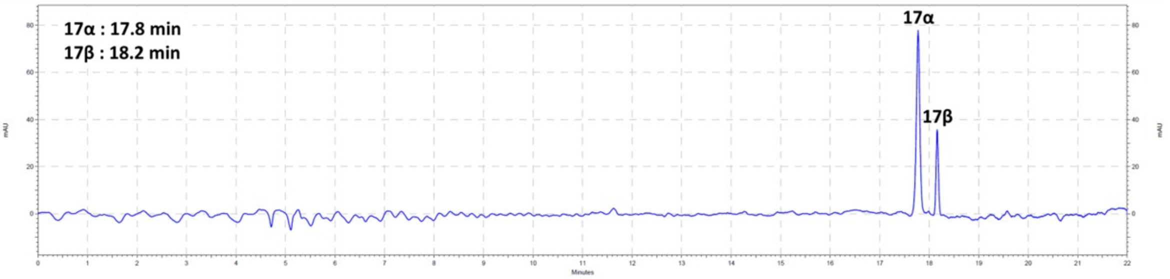

Figure S22: a HPLC spectrum of **17α**, **17β** (Method A)

Machine Learning software development

Overview

Many software development platforms exist for developing machine learning software based on machine learning algorithms. The popular choices includes Python based TensorFlow, R studio, or MATLAB.

In this study, statistical and machine learning toolbox in MATLAB (R2018a) was used for the development machine learning code. The core machine learning algorithm is based on random forest algorithm. Random forest algorithm generates several weak models (learners) in the form of binary decision trees. The nodes of each of these decision trees are generated by random shuffling of features (descriptors) in the training set. The final model outcome is generated by creating an "ensemble" by a combined weighted sum of these generated decision trees, representing a collective decision of all the individual trees, dictating the final output prediction of the model. Modelling the data with these ensemble learners generates good prediction and reduces overfitting.

Random forest algorithm was used in MATLAB by invoking the "`fitrensemble`" function. The general syntax for the function used is

```
"Mdl =
fitrensemble(X,Y,'Method','Learners','OptimizeHyperparameters'
,      'HyperparameterOptimizationOptions')
```

`Mdl` is the model output as a "Regression Ensemble" which is a complex data structure consisting of the trained model along with compiled information on every parameters including function weights, fit info, and hyperparameter optimizations results. This also contain the trained model that will be later used in the prediction section. `X` is the input variable which contains experimental data along with the input descriptors. `Y` is the response variable containing the experimental observations. '`Leaners`' is the specification for the type of decision tree or model.

The machine learning code developed in this study can be divided into 5 main sections:

1) The Data input and preconditioning section
2) Model Input section
3) Machine learning and Data processing section
4) Prediction section
5) Data output section

1. Data input and preconditioning section:

Data input and preconditioning section consists of codes to import the training set data having both descriptor input data and response data into different arrays using '`xlsread`' function of MATLAB. Arrays were also created for other functions like storing the model output data in the preconditioning section. Validation and experimental data were also stored into separate arrays to be used for prediction and validation purposes.

2. Model Input Section:

This section of the code mainly deals with the generation of the learner function which is based on regression tree. Model tree can be generated using the "templatetree" function. The template tree function could be invoked as shown below:

```
t = templateTree('NumPredictorsToSample',
'PredictorSelection','Prune','Surrogate',);
```

The templateTree function generates decision trees and in this case regression trees based on a number of nested functions including "NumPredictorToSample" which selects the appropriate number of predictors to the random sampling of data. Selection of appropriate splitting condition of decision trees are very important and to find this split condition, algorithm based on Interaction Curvature algorithm is used. Further length and split conditions of the decision trees were regulated by pruning and surrogate split algorithms.

3. Machine Learning and Data Processing section:

The optimal template tree as described in the previous section is used in the random forest algorithm as learners. The trees were randomly selected and grown using algorithm based on "Bagging" and "LSBoost" type algorithm.

Bagging Algorithm:

Bagging algorithm is an ensemble learning technology called Bootstrap Aggregation. With this technology, random replica models or decision trees are grown on the all the samples in the training set. With this technique many replica models could be generated from the same training set. For generating the splits in the decision tree, the predictor is randomly selected. This random selection of predictors leads to what is called a Random Forest.[11]

LSBoost:

This algorithm is used here for regression based ensemble learning. The least square boosting is done to fit the regression trees with the observed data. At every iteration, the algorithm works by fitting a new learner with the observed difference between prediction from the model and observed data.[12] This fitting is done by minimization of the mean-square-error (MSE).

Tuning of Hyperparameters

The learning performance of machine learning algorithms can be enhanced quite significantly by choosing proper hyper-parameters. However, this optimization is still empirical in nature and often depends on the dataset being optimized. As an alternative, automated hyperparameter tuning is becoming increasingly important.[13] Here we have used the algorithm. "Expected-improvement-plus" in MATLAB for automated tuning of hyper parameters.

4. Prediction Section:

Upon completion of the training, the model output is stored in the variable 'Mdl' as discussed previously. The prediction algorithm 'predict' can now be used to along with the trained model stored in 'Mdl' to predict new experimental results based on the trained model. Once the prediction is completed, the predictor importance is calculated by summation of the variation

of Mean square errors (MSE) which generates from split of each predictor and dividing this quantity by the total number of branch nodes. As the tree here is grown with surrogate splits, the summation of the MSE is done at each branch nodes along with surrogate splits. Separate arrays are created for storing the prediction results and the predictor importance.

5. Data output Section:

The arrays generated for storing the model output in the form of prediction data and predictor importance along with R^2 are exported and converted into table data types in this section. These tables are then written to Microsoft Excel datasheets using the 'xlswrite' function of MATLAB.

Software for screening of different Machine Learning Algorithms as a benchmark study

Along with regression based Random Forest (RF) algorithm, which was primarily used as the core algorithm in this present study, separate software was written for screening of other regression based machine learning algorithms. These are Gaussian Process Regression (GPR), Regression Tree (RT) and regression based Support Vector Machine (SVM) following a similar software design methodology as described above. For implementation of GPR, SVM and Regression Tree in MATLAB, 'fitrgp', 'fitrsvm' and 'fitrtree' functions were used respectively using similar methodology as described for the implementation of random forest in MATLAB. This additional study was done in order to compare the prediction performance of different algorithm compared to Random Forest. Each of the four ML algorithms was trained using the training set and the models were compared with the validation set.

XYZ Coordinates

The xyz coordinates for all optimized structures are provided below. Levels of theory for each are given in previous sections.

Electrophile

Glc1α

N	0.797797	-3.866059	2.237765
C	0.823887	-2.617078	2.423338
C	0.650994	-2.027420	3.836995
Cl	2.114927	-1.055263	4.250686
Cl	-0.800808	-0.941707	3.824682
Cl	0.419698	-3.293013	5.063734
O	0.984897	-1.599334	1.540935
C	1.306992	-1.925777	0.179156
C	1.762229	-0.633913	-0.505343
O	2.844804	-0.090498	0.216032
C	3.910689	0.416971	-0.584008
C	4.893168	1.164952	0.288847
C	5.949816	1.857404	-0.318041
C	6.880987	2.549863	0.453895
C	6.764487	2.562993	1.846602
C	5.712775	1.878489	2.455136
C	4.780343	1.181498	1.682219
C	0.595871	0.357434	-0.625772
O	1.056814	1.428506	-1.430102
C	0.466031	2.714025	-1.181367
C	1.392489	3.776930	-1.714100
C	2.613119	4.027329	-1.072305
C	3.487691	4.993597	-1.565176
C	3.147794	5.727730	-2.705438
C	1.932896	5.488362	-3.348663
C	1.061749	4.514457	-2.854766
C	-0.625170	-0.338960	-1.261208
O	-1.757409	0.522563	-1.358826
C	-2.475699	0.813880	-0.156963
C	-3.357065	2.022678	-0.379460
C	-3.697267	2.837107	0.706808
C	-4.548666	3.929359	0.537626
C	-5.064662	4.223733	-0.725668
C	-4.725431	3.418101	-1.814251
C	-3.879441	2.321135	-1.643347
C	-0.962265	-1.680702	-0.575502
C	-2.022998	-2.468828	-1.351457
O	-2.853128	-3.278097	-0.544412

C	-2.223891	-4.440235	-0.009113
C	-3.264106	-5.340839	0.612507
C	-2.980052	-6.016255	1.804342
C	-3.913713	-6.891524	2.362472
C	-5.146457	-7.089306	1.739234
C	-5.439668	-6.409365	0.554559
C	-4.502762	-5.543181	-0.007591
O	0.225797	-2.507411	-0.490705
H	0.878464	-4.113107	1.250217
H	2.111546	-2.668049	0.176329
H	2.074330	-0.924606	-1.518934
H	3.513004	1.082235	-1.359038
H	4.420414	-0.420120	-1.092418
H	6.041686	1.856439	-1.402578
H	7.695671	3.080924	-0.031813
H	7.487608	3.105089	2.450232
H	5.613883	1.884121	3.537795
H	3.959587	0.651637	2.152720
H	0.357561	0.719077	0.384155
H	0.328196	2.840007	-0.096239
H	-0.519168	2.776611	-1.656035
H	2.879030	3.459258	-0.184021
H	4.432124	5.172465	-1.057970
H	3.827095	6.484878	-3.088509
H	1.662898	6.055501	-4.235943
H	0.116365	4.324906	-3.358328
H	-0.360686	-0.543321	-2.306358
H	-1.791545	1.007213	0.679929
H	-3.093668	-0.052380	0.124162
H	-3.291496	2.617090	1.692381
H	-4.800436	4.554347	1.390411
H	-5.723463	5.077378	-0.861537
H	-5.121124	3.642760	-2.801493
H	-3.610278	1.697365	-2.489529
H	-1.334854	-1.503680	0.439914
H	-1.516997	-3.062768	-2.130107
H	-2.692581	-1.755044	-1.838894
H	-1.475965	-4.168869	0.747020
H	-1.689452	-4.966144	-0.820161
H	-2.025565	-5.851668	2.300378
H	-3.680640	-7.409147	3.289350
H	-5.877829	-7.764188	2.176359
H	-6.401030	-6.554011	0.068046
H	-4.730767	-5.006530	-0.923476

Glc1β

N	0.671274	-4.452114	1.121546
C	1.761251	-3.922271	0.778582
C	3.099255	-4.648156	1.010921
Cl	4.021134	-3.728197	2.273197
Cl	2.852383	-6.320257	1.568852
Cl	4.054081	-4.668494	-0.522032
O	2.008917	-2.708089	0.202578
C	0.996591	-1.724283	0.286816
C	1.537876	-0.428185	-0.315953
O	2.542198	0.128716	0.503964
C	3.876764	0.051631	-0.025220
C	4.785315	0.866141	0.857362
C	4.811751	2.262893	0.747957
C	5.646076	3.019979	1.569750
C	6.461653	2.386832	2.511509
C	6.436154	0.996717	2.630617
C	5.598685	0.241755	1.807925
C	0.416342	0.616795	-0.457485
O	0.930909	1.637562	-1.293531
C	0.657352	2.973618	-0.893032
C	1.471676	3.937293	-1.727456
C	1.549984	5.280031	-1.335898
C	2.268771	6.200365	-2.096581
C	2.925140	5.787333	-3.258378
C	2.853687	4.451427	-3.651414
C	2.129303	3.530174	-2.892143
C	-0.887322	0.032940	-1.039743
O	-1.953673	0.979369	-1.052151
C	-2.549381	1.330035	0.194730
C	-3.381435	2.582561	0.020749
C	-3.713396	3.347132	1.145967
C	-4.517278	4.479995	1.022493
C	-4.993868	4.865606	-0.231954
C	-4.662789	4.110161	-1.357233
C	-3.863137	2.972017	-1.233401
C	-1.242691	-1.294136	-0.342212
C	-2.428645	-2.027755	-0.941570
O	-2.748100	-3.085568	-0.060578
C	-3.746188	-3.967627	-0.568629
C	-3.946079	-5.106759	0.400553
C	-2.854821	-5.885282	0.810159
C	-3.036679	-6.945740	1.695992
C	-4.313739	-7.249465	2.175351
C	-5.405615	-6.481625	1.769895
C	-5.219252	-5.411769	0.891302
O	-0.117365	-2.173091	-0.455886
H	-0.134314	-3.875564	0.868598
H	0.720576	-1.586388	1.345356
H	1.916637	-0.666952	-1.318704
H	3.876088	0.451011	-1.048928
H	4.206577	-0.994604	-0.061705
H	4.178011	2.753049	0.011782
H	5.665487	4.102061	1.471019
H	7.117257	2.976171	3.147910
H	7.070368	0.499875	3.360255
H	5.577897	-0.841680	1.898633
H	0.229428	1.014590	0.551100
H	0.910234	3.101950	0.170390
H	-0.415671	3.189612	-1.005875
H	1.044145	5.606428	-0.428878
H	2.321328	7.238371	-1.779342
H	3.489766	6.502150	-3.850511
H	3.363501	4.121529	-4.553274
H	2.071721	2.490571	-3.194852
H	-0.704945	-0.176417	-2.099524
H	-1.789938	1.500216	0.971188
H	-3.188276	0.505748	0.551322
H	-3.338760	3.056333	2.125762
H	-4.762788	5.065748	1.904283
H	-5.615387	5.750997	-0.331403
H	-5.026488	4.406429	-2.337710
H	-3.599780	2.386599	-2.107561
H	-1.453352	-1.121328	0.725217
H	-2.154729	-2.407677	-1.939240
H	-3.274563	-1.333834	-1.063339
H	-3.425073	-4.345516	-1.554016

H	-4.692445	-3.425008	-0.721457
H	-1.858208	-5.655376	0.444253
H	-2.179531	-7.534803	2.011121
H	-4.455136	-8.078625	2.863656
H	-6.401557	-6.708359	2.141701
H	-6.071154	-4.807526	0.586487

Glc2β

C	-6.716277	-1.204385	1.468287
C	-5.255131	-0.854908	1.187057
S	-4.568764	-2.044924	-0.039960
C	-2.795581	-1.614615	-0.030699
C	-2.472787	-0.116792	-0.221270
O	-3.113566	0.502264	-1.328357
C	-2.984104	-0.118090	-2.607816
C	-3.426294	0.857132	-3.675981
C	-4.422366	1.806243	-3.418441
C	-4.842458	2.678283	-4.423162
C	-4.279029	2.607392	-5.698820
C	-3.288375	1.660338	-5.963620
C	-2.862665	0.794263	-4.955360
C	-0.941699	0.084659	-0.226149
O	-0.592687	1.459213	-0.150297
C	-0.467590	2.150805	-1.389074
C	0.168291	3.502169	-1.155127
C	0.897613	4.110630	-2.183851
C	1.450691	5.379556	-2.011307
C	1.287151	6.053700	-0.800525
C	0.564871	5.452071	0.231369
C	0.005346	4.186129	0.055377
C	-0.260740	-0.595418	0.975114
O	1.137552	-0.624467	0.729511
C	1.925762	0.215844	1.589321
C	3.297797	0.371513	0.984824
C	4.384436	-0.373895	1.453290
C	5.645929	-0.229295	0.871450
C	5.829934	0.661598	-0.186466
C	4.748812	1.409308	-0.661150
C	3.491315	1.263912	-0.078150
C	-0.780232	-2.026277	1.185384
C	-0.410211	-2.648178	2.543856
O	0.964797	-2.667756	2.860897
C	1.747574	-3.553437	2.070648
C	3.059229	-3.841949	2.766551
C	4.176079	-4.216526	2.009733
C	5.380657	-4.542999	2.633272
C	5.485788	-4.485578	4.024314
C	4.379696	-4.101142	4.784000
C	3.171619	-3.785621	4.160117
O	-2.212052	-2.041516	1.194010
H	-7.137787	-0.492081	2.185751
H	-7.318502	-1.157587	0.555156
H	-6.812456	-2.210204	1.888936
H	-4.655968	-0.921707	2.099950
H	-5.174911	0.158047	0.781645
H	-2.378210	-2.203203	-0.863043
H	-2.860050	0.423628	0.647989
H	-3.609473	-1.022364	-2.639932
H	-1.946693	-0.425366	-2.800749
H	-4.852445	1.863656	-2.424321
H	-5.611321	3.415891	-4.208038
H	-4.605259	3.288769	-6.479687
H	-2.838962	1.601350	-6.952052
H	-2.082819	0.064597	-5.164042
H	-0.504308	-0.359946	-1.134087
H	-1.455362	2.270150	-1.852050
H	0.153207	1.565226	-2.084209
H	1.033174	3.586785	-3.128164
H	2.015428	5.836424	-2.819726
H	1.722183	7.039015	-0.660553
H	0.435832	5.969750	1.178592
H	-0.553113	3.716145	0.857509
H	-0.490997	-0.005625	1.873708
H	1.984032	-0.239813	2.585168
H	1.440680	1.195325	1.670512
H	4.244235	-1.070909	2.275186
H	6.482475	-0.813027	1.246572
H	6.812259	0.777320	-0.637592
H	4.888037	2.110012	-1.480429
H	2.649141	1.850405	-0.437611
H	-0.410537	-2.655449	0.358850
H	-0.835134	-3.663878	2.576638
H	-0.892693	-2.056325	3.328968
H	1.935943	-3.114469	1.081515
H	1.189843	-4.494931	1.918905
H	4.102537	-4.247818	0.924718
H	6.238607	-4.833031	2.032385
H	6.424860	-4.733189	4.512198
H	4.456048	-4.048294	5.867128
H	2.310152	-3.482532	4.745504

Glc3α

O	3.685392	0.618087	-0.961597
P	2.801164	-0.562625	-0.811993
O	3.090601	-1.782985	-1.823177
C	3.193204	-1.490206	-3.239817
C	3.503871	-2.787784	-3.969886
C	3.647263	-2.583044	-5.485055
C	3.969214	-3.882808	-6.228861
O	2.771955	-1.321439	0.588807
C	3.958416	-1.391039	1.422457
C	3.514665	-1.465019	2.874241
C	4.706777	-1.531822	3.837292
C	4.275178	-1.558899	5.307161
O	1.229776	-0.284245	-1.062355
C	0.682599	1.050979	-1.135717
C	0.101476	1.457216	0.221863
O	1.138272	1.438364	1.185064
C	1.158527	2.553484	2.080285
C	2.453296	2.548111	2.855579
C	2.444804	2.701873	4.245444
C	3.642116	2.751291	4.963479
C	4.861211	2.642254	4.294548
C	4.876942	2.478147	2.905819
C	3.682022	2.431590	2.188245
C	-1.054007	0.533342	0.618681
O	-1.665896	1.094905	1.775080
C	-2.001125	0.165676	2.803241
C	-2.591149	0.915770	3.974594
C	-2.282778	0.536522	5.284059
C	-2.859040	1.194902	6.371730
C	-3.745145	2.250712	6.159044

C	-4.054490	2.641525	4.853122
C	-3.484524	1.976471	3.767901
C	-2.070337	0.432541	-0.532958
O	-3.122390	-0.498663	-0.265676
C	-2.802268	-1.883573	-0.262855
C	-3.846827	-2.658284	0.514839
C	-3.643478	-4.024874	0.751695
C	-4.590204	-4.774000	1.447905
C	-5.755507	-4.163953	1.920452
C	-5.962506	-2.804824	1.688300
C	-5.015206	-2.054803	0.986725
C	-1.400203	0.171569	-1.899966
C	-2.356979	0.364378	-3.066350
O	-2.914789	1.664842	-2.993367
C	-3.716631	2.012048	-4.109774
C	-2.922328	2.320606	-5.367747
C	-1.623304	2.836324	-5.278829
C	-0.914987	3.167056	-6.434743
C	-1.497581	2.993681	-7.691555
C	-2.791277	2.477185	-7.787154
C	-3.495381	2.137658	-6.630582
O	-0.298136	1.071462	-2.121755
H	3.983379	-0.746469	-3.391708
H	2.241585	-1.060806	-3.581543
H	2.705886	-3.512479	-3.761341
H	4.430063	-3.210719	-3.557149
H	4.436644	-1.844800	-5.679886
H	2.718962	-2.151307	-5.886725
H	4.912029	-4.316493	-5.874703
H	3.183146	-4.631866	-6.077224
H	4.063771	-3.711663	-7.306690
H	4.574139	-0.504607	1.244102
H	4.524946	-2.281880	1.126237
H	2.871065	-2.344112	3.009281
H	2.904018	-0.580323	3.088114
H	5.356508	-0.663303	3.665978
H	5.312164	-2.422109	3.615671
H	3.730907	-0.644602	5.569563
H	3.616450	-2.412494	5.513140
H	5.141548	-1.637427	5.974239
H	1.470592	1.738648	-1.446452
H	-0.293229	2.476463	0.101805
H	0.294808	2.514679	2.754211
H	1.077885	3.484168	1.493943
H	1.494923	2.784820	4.769694
H	3.620036	2.875110	6.043303
H	5.794386	2.684542	4.849879
H	5.823761	2.392280	2.378217
H	3.697482	2.283448	1.111955
H	-0.622160	-0.445806	0.858734
H	-1.103149	-0.384436	3.121611
H	-2.724969	-0.569463	2.421870
H	-1.583341	-0.279263	5.454037
H	-2.604466	0.889027	7.384664
H	-4.188796	2.768879	7.004825
H	-4.740860	3.466190	4.680619
H	-3.718492	2.279923	2.751546
H	-2.583535	1.395564	-0.593410
H	-1.812709	-2.062918	0.179330
H	-2.760430	-2.266786	-1.296128
H	-2.736161	-4.505686	0.390611
H	-4.416876	-5.832233	1.625709
H	-6.493008	-4.745323	2.467351
H	-6.864763	-2.320505	2.053738
H	-5.173282	-0.997668	0.804173
H	-1.017986	-0.855909	-1.940248
H	-3.152046	-0.396258	-3.013327
H	-1.802748	0.225013	-4.005172
H	-4.269906	2.903828	-3.789431
H	-4.459139	1.223485	-4.316153
H	-1.168698	2.956704	-4.299486
H	0.094985	3.560886	-6.353003
H	-0.945458	3.254458	-8.590967
H	-3.249541	2.329186	-8.761827
H	-4.500589	1.726729	-6.711460

Gal1α

N	-1.290125	-1.382621	4.670030
C	-0.789584	-0.239439	4.489237
C	-0.567927	0.719863	5.674156
Cl	1.203024	1.052536	5.832645
Cl	-1.433532	2.275464	5.333906
Cl	-1.175141	0.038964	7.200671
O	-0.369799	0.367550	3.344992
C	-0.165832	-0.466095	2.190113
C	0.493679	0.404151	1.110453
O	1.670088	0.997718	1.625630
C	2.840990	0.778917	0.840967
C	4.016157	1.475454	1.486317
C	5.087243	1.906877	0.694291
C	6.202227	2.511527	1.276597
C	6.252984	2.699773	2.658849
C	5.184383	2.278495	3.452551
C	4.072823	1.666451	2.871839
C	-0.499185	1.460180	0.605607
O	0.080500	2.224967	-0.442590
C	0.576031	3.512136	-0.037680
C	1.286259	4.141431	-1.209192
C	2.657433	4.411118	-1.150453
C	3.314082	4.990666	-2.238835
C	2.605146	5.297419	-3.400001
C	1.235247	5.026869	-3.468878
C	0.580942	4.456143	-2.378923
C	-1.768812	0.743310	0.103585
O	-1.508809	-0.121307	-0.989638
C	-1.517574	0.466850	-2.302385
C	-1.531611	-0.661994	-3.301383
C	-2.733906	-1.108903	-3.861917
C	-2.751849	-2.196812	-4.735990
C	-1.561383	-2.852522	-5.057071
C	-0.355307	-2.412539	-4.505475
C	-0.342324	-1.322108	-3.635811
C	-2.339831	-0.105862	1.252484
C	-3.624484	-0.917531	0.886517
O	-3.484652	-2.314718	0.918433
C	-2.867772	-2.895761	-0.223384
C	-2.821687	-4.400193	-0.077086
C	-2.341717	-5.168754	-1.146515
C	-2.256740	-6.555996	-1.041599
C	-2.652710	-7.196011	0.136100
C	-3.134416	-6.436314	1.201157
C	-3.217832	-5.045384	1.097272

O	-1.358187	-1.038825	1.736179
H	-1.434296	-1.881027	3.788896
H	0.479474	-1.301945	2.479089
H	0.722887	-0.264960	0.272669
H	2.689246	1.155101	-0.179617
H	3.034308	-0.304904	0.766017
H	5.047017	1.771551	-0.384685
H	7.026559	2.841982	0.649654
H	7.117629	3.176073	3.113599
H	5.214453	2.427727	4.528692
H	3.235539	1.349020	3.484567
H	-0.775749	2.114497	1.443727
H	1.254460	3.400450	0.814537
H	-0.273664	4.136671	0.282784
H	3.214440	4.163503	-0.250431
H	4.378853	5.198466	-2.178332
H	3.114812	5.746717	-4.248312
H	0.677464	5.265716	-4.370965
H	-0.485047	4.246182	-2.432519
H	-2.520028	1.498956	-0.181387
H	-0.638998	1.105790	-2.429421
H	-2.415203	1.094344	-2.414455
H	-3.662792	-0.602000	-3.609339
H	-3.692631	-2.532368	-5.164839
H	-1.572380	-3.699543	-5.737930
H	0.574246	-2.916283	-4.756721
H	0.596728	-0.980109	-3.206047
H	-2.591895	0.586650	2.067363
H	-3.989530	-0.581836	-0.096587
H	-4.396897	-0.691257	1.627983
H	-1.855517	-2.498396	-0.354076
H	-3.432362	-2.620951	-1.129558
H	-2.033543	-4.674660	-2.066762
H	-1.880281	-7.138258	-1.878575
H	-2.586779	-8.277755	0.220111
H	-3.445864	-6.925715	2.120862
H	-3.591170	-4.452573	1.924238

Gal1β

N	0.240212	-3.656398	2.456076
C	1.266674	-3.093909	1.990583
C	2.669910	-3.691766	2.205480
Cl	3.670431	-2.500792	3.133271
Cl	2.604418	-5.225465	3.104934
Cl	3.436944	-3.978183	0.593491
O	1.386971	-1.943281	1.265085
C	0.294702	-1.042318	1.279821
C	0.720068	0.238222	0.549605
O	1.721130	0.920675	1.283517
C	3.046792	0.799426	0.759948
C	3.989431	1.616924	1.609927
C	5.063977	2.291586	1.018791
C	5.965674	3.017140	1.799729
C	5.794208	3.086004	3.182853
C	4.719971	2.421279	3.778945
C	3.825613	1.688352	2.999135
C	-0.492478	1.172190	0.391270
O	-0.152109	2.281828	-0.425022
C	0.203010	3.480927	0.285125
C	0.657895	4.509308	-0.718894
C	1.998112	4.906408	-0.775109
C	2.420330	5.849527	-1.714814
C	1.505538	6.397066	-2.614635
C	0.165228	6.003783	-2.567945
C	-0.254431	5.069271	-1.623312
C	-1.684361	0.422463	-0.242694
O	-1.396111	0.036532	-1.576182
C	-1.855679	0.940546	-2.595503
C	-1.071787	0.678942	-3.855337
C	-1.627240	-0.029961	-4.923816
C	-0.880981	-0.274407	-6.078907
C	0.432357	0.186962	-6.169725
C	0.996376	0.894133	-5.104066
C	0.248749	1.138809	-3.953954
C	-1.960238	-0.844280	0.577320
C	-3.061884	-1.724279	0.012201
O	-3.347016	-2.715991	0.978910
C	-4.219917	-3.733613	0.499840
C	-4.357708	-4.807321	1.551052
C	-3.217505	-5.436495	2.069973
C	-3.339305	-6.431555	3.038397
C	-4.603102	-6.817751	3.493598
C	-5.742533	-6.200959	2.977683
C	-5.617371	-5.196534	2.014823
O	-0.785531	-1.660530	0.625032
H	-0.618986	-3.177562	2.174347
H	0.034595	-0.820252	2.329612
H	1.070620	-0.041085	-0.450186
H	3.068671	1.157989	-0.279162
H	3.344870	-0.257459	0.755626
H	5.198487	2.246876	-0.060293
H	6.795901	3.534733	1.325840
H	6.490773	3.655876	3.792440
H	4.577904	2.473648	4.855452
H	2.987412	1.175248	3.459440
H	-0.796827	1.518795	1.391689
H	0.995954	3.266486	1.006954
H	-0.678913	3.843494	0.838113
H	2.713556	4.475293	-0.078356
H	3.463433	6.152809	-1.744345
H	1.832737	7.129017	-3.348212
H	-0.551587	6.429627	-3.265461
H	-1.299055	4.769304	-1.584010
H	-2.572128	1.071855	-0.206127
H	-1.702130	1.971158	-2.263362
H	-2.931694	0.781905	-2.767244
H	-2.650622	-0.392169	-4.852750
H	-1.324792	-0.824813	-6.904318
H	1.015317	-0.001252	-7.067731
H	2.018287	1.257975	-5.173157
H	0.678202	1.691749	-3.122335
H	-2.241893	-0.548891	1.602099
H	-2.716385	-2.168892	-0.931802
H	-3.954632	-1.112254	-0.201441
H	-3.806537	-4.153817	-0.433010
H	-5.206382	-3.307728	0.255446
H	-2.231972	-5.139542	1.722060
H	-2.446244	-6.904747	3.437264
H	-4.696637	-7.593492	4.248240
H	-6.728991	-6.493328	3.328453
H	-6.507743	-4.709066	1.623727

Gal2β

C	-5.704275	-1.636518	0.803668
C	-4.200284	-1.394355	0.667215
S	-3.545007	-2.423567	-0.712821
C	-1.767648	-1.986296	-0.688163
C	-1.454692	-0.553544	-1.160065
O	-1.775698	-0.466700	-2.537024
C	-2.417550	0.749339	-2.927949
C	-2.648358	0.734813	-4.419030
C	-2.400582	1.882840	-5.178729
C	-2.652700	1.895358	-6.552189
C	-3.146060	0.752786	-7.181553
C	-3.387738	-0.401189	-6.430984
C	-3.144171	-0.408851	-5.057779
C	0.039979	-0.213531	-0.976543
O	0.138886	1.193478	-1.126010
C	1.459748	1.697551	-1.343517
C	1.371459	3.199671	-1.411800
C	1.317112	3.865661	-2.641053
C	1.195374	5.255324	-2.691249
C	1.122478	5.990652	-1.507594
C	1.171950	5.333308	-0.274917
C	1.296775	3.944771	-0.229032
C	0.691754	-0.715367	0.341050
O	0.624042	0.205222	1.418270
C	-0.644339	0.676985	1.863734
C	-0.460351	1.416669	3.172272
C	0.667600	1.214736	3.974956
C	0.789499	1.876212	5.198558
C	-0.213192	2.741581	5.636411
C	-1.340549	2.948916	4.838562
C	-1.458901	2.293677	3.613515
C	0.176514	-2.129395	0.663987
C	0.597744	-2.627697	2.050645
O	2.006862	-2.715050	2.167103
C	2.566512	-3.944229	1.727976
C	4.040968	-3.979531	2.064061
C	4.544638	-3.286743	3.170969
C	5.899353	-3.365633	3.493022
C	6.763935	-4.144287	2.722541
C	6.267025	-4.838132	1.617556
C	4.914224	-4.750083	1.287889
O	-1.250943	-2.204865	0.617202
H	-6.105424	-1.037249	1.629275
H	-6.238239	-1.355353	-0.110156
H	-5.920079	-2.689015	1.013736
H	-3.674625	-1.671113	1.584556
H	-4.005515	-0.336205	0.463522
H	-1.314843	-2.691303	-1.403808
H	-2.055358	0.154127	-0.577060
H	-1.801642	1.609909	-2.643727
H	-3.379488	0.831417	-2.393515
H	-2.003952	2.771578	-4.692159
H	-2.453431	2.795118	-7.130086
H	-3.337755	0.758021	-8.251887
H	-3.768218	-1.295982	-6.915518
H	-3.326260	-1.304821	-4.471419
H	0.562310	-0.718982	-1.806826
H	1.866085	1.283595	-2.279773
H	2.117033	1.394568	-0.518588
H	1.374100	3.290971	-3.563416
H	1.158648	5.761854	-3.652638
H	1.029125	7.073485	-1.543493
H	1.117497	5.902369	0.648650
H	1.332787	3.429038	0.727914
H	1.770523	-0.810945	0.179879
H	-1.338695	-0.162601	2.006351
H	-1.078655	1.348772	1.111875
H	1.449131	0.546510	3.629059
H	1.674286	1.714033	5.810449
H	-0.115918	3.254709	6.589233
H	-2.124515	3.626248	5.166056
H	-2.336274	2.466534	2.992113
H	0.588109	-2.811088	-0.101271
H	0.115503	-3.597085	2.240409
H	0.265781	-1.919344	2.812902
H	2.428552	-4.086237	0.643234
H	2.043053	-4.779787	2.225427
H	3.868273	-2.679567	3.763310
H	6.279723	-2.817502	4.351732
H	7.818409	-4.206816	2.977071
H	6.933446	-5.440781	1.005526
H	4.535121	-5.285070	0.419900

Gal3α

O	3.370989	0.037613	-0.893012
P	2.536444	-1.168174	-0.672434
O	2.552939	-2.276864	-1.833744
C	2.194521	-1.898975	-3.189475
C	2.428065	-3.105830	-4.084736
C	2.045183	-2.832832	-5.544597
C	2.302894	-4.034691	-6.457395
O	2.835681	-2.061460	0.613398
C	4.199950	-2.281875	1.055733
C	4.158533	-2.672831	2.523895
C	5.561401	-2.886390	3.105203
C	5.535281	-3.237977	4.596019
O	0.957364	-0.867829	-0.490219
C	0.448211	0.471954	-0.407146
C	-0.075914	0.728299	1.011579
O	0.983626	0.436105	1.898355
C	1.068782	1.233452	3.079245
C	2.473211	1.152031	3.631521
C	2.681170	1.020763	5.008357
C	3.974406	0.994015	5.535479
C	5.076044	1.092451	4.684626
C	4.876244	1.215315	3.306265
C	3.584622	1.245476	2.780586
C	-1.329897	-0.108599	1.310247
O	-1.883765	0.410469	2.507607
C	-2.863366	-0.424963	3.132315
C	-3.370356	0.280834	4.363597
C	-2.994186	-0.138484	5.642756
C	-3.457220	0.533855	6.775610
C	-4.296086	1.639349	6.635309
C	-4.673611	2.068146	5.359773
C	-4.214124	1.391833	4.231217
C	-2.366824	-0.147692	0.144956
O	-3.346188	0.883333	0.183573
C	-2.925088	2.244264	0.198729
C	-4.128604	3.147212	0.021975
C	-5.428816	2.645212	-0.083075

C	-6.510939	3.517448	-0.230165
C	-6.305547	4.896177	-0.273968
C	-5.007415	5.403537	-0.170400
C	-3.928611	4.534124	-0.024203
C	-1.651818	-0.298164	-1.209453
C	-2.581601	-0.118367	-2.406575
O	-1.937354	-0.433999	-3.629957
C	-1.398211	0.687148	-4.325057
C	-0.899850	0.251372	-5.683686
C	0.196871	0.897596	-6.265448
C	0.637227	0.544334	-7.542072
C	-0.008952	-0.471574	-8.248553
C	-1.098447	-1.127323	-7.670631
C	-1.543692	-0.766405	-6.398596
O	-0.565992	0.639646	-1.354835
H	2.812922	-1.046789	-3.495603
H	1.141431	-1.591352	-3.201426
H	1.843860	-3.950133	-3.694485
H	3.485624	-3.394829	-4.015565
H	2.606160	-1.965598	-5.917519
H	0.986025	-2.554385	-5.601087
H	3.364056	-4.310481	-6.465549
H	1.735790	-4.912479	-6.125684
H	2.005830	-3.815213	-7.489490
H	4.776106	-1.363240	0.904960
H	4.636416	-3.076470	0.438679
H	3.560880	-3.587342	2.633104
H	3.639461	-1.880954	3.075893
H	6.154206	-1.972833	2.960570
H	6.077415	-3.681549	2.548299
H	5.070971	-2.432494	5.178130
H	4.964494	-4.156806	4.779521
H	6.548968	-3.392414	4.985074
H	1.242963	1.175516	-0.659264
H	-0.328976	1.795208	1.070727
H	0.334333	0.901463	3.822267
H	0.823185	2.279458	2.828346
H	1.824615	0.936626	5.673329
H	4.119045	0.890994	6.607732
H	6.084244	1.071160	5.090476
H	5.730044	1.290368	2.637011
H	3.436502	1.318122	1.706573
H	-0.981990	-1.142097	1.467011
H	-2.412179	-1.394915	3.393287
H	-3.693444	-0.609364	2.436657
H	-2.335165	-0.997723	5.753529
H	-3.160245	0.195281	7.764683
H	-4.657253	2.164217	7.515262
H	-5.328829	2.928478	5.244629
H	-4.506763	1.723194	3.237909
H	-2.973142	-1.052250	0.273178
H	-2.197191	2.435117	-0.600781
H	-2.440893	2.468481	1.158817
H	-5.583518	1.572001	-0.054335
H	-7.517270	3.114425	-0.312117
H	-7.148704	5.572226	-0.387984
H	-4.836617	6.476920	-0.202833
H	-2.919670	4.935136	0.056540
H	-1.232253	-1.309991	-1.259252
H	-2.993905	0.897965	-2.426638
H	-3.420867	-0.815133	-2.295202
H	-0.585231	1.146316	-3.750771
H	-2.187589	1.450224	-4.443120
H	0.712744	1.682748	-5.715646
H	1.490703	1.056781	-7.979177
H	0.335908	-0.751973	-9.240084
H	-1.605114	-1.921643	-8.212871
H	-2.385275	-1.280040	-5.944205

Man1α

N	1.151120	-3.401222	3.256559
C	1.235435	-2.151795	3.416428
C	1.114833	-1.526777	4.820796
Cl	2.608717	-0.568122	5.168103
Cl	-0.315501	-0.413492	4.824017
Cl	0.899080	-2.758078	6.082293
O	1.423173	-1.157589	2.511696
C	1.686500	-1.519578	1.139054
C	2.150737	-0.235642	0.443194
O	2.596169	-0.645775	-0.837236
C	3.735390	0.055988	-1.341266
C	4.055656	-0.442029	-2.730998
C	3.718640	-1.736777	-3.141198
C	4.058316	-2.180765	-4.419697
C	4.745100	-1.341161	-5.298470
C	5.084480	-0.048680	-4.894616
C	4.735414	0.398398	-3.620371
C	0.987671	0.767333	0.334798
O	1.457277	1.857066	-0.441485
C	0.905828	3.138674	-0.130331
C	1.522698	4.169829	-1.045019
C	1.900919	5.421999	-0.550343
C	2.423345	6.396172	-1.403646
C	2.584883	6.121415	-2.761698
C	2.216912	4.869756	-3.262176
C	1.684750	3.901908	-2.411225
C	-0.220533	0.075590	-0.322977
O	-1.351100	0.936470	-0.452114
C	-2.104710	1.225068	0.726346
C	-2.975507	2.438737	0.484390
C	-3.443527	3.175620	1.579236
C	-4.282043	4.273791	1.390517
C	-4.657293	4.653063	0.099928
C	-4.190437	3.925459	-0.995398
C	-3.356961	2.821360	-0.806214
C	-0.582276	-1.245734	0.394995
C	-1.653964	-2.036667	-0.364381
O	-2.538357	-2.755582	0.472765
C	-1.969291	-3.905585	1.092837
C	-3.051846	-4.704504	1.778485
C	-2.782668	-5.343134	2.994012
C	-3.756956	-6.127923	3.613694
C	-5.015948	-6.270593	3.028902
C	-5.294113	-5.625906	1.821265
C	-4.316834	-4.850963	1.197199
O	0.580740	-2.098338	0.524086
H	1.200000	-3.671208	2.272673
H	2.479415	-2.272840	1.109714
H	2.969571	0.209735	1.023285
H	4.590893	-0.119908	-0.665764
H	3.538338	1.132281	-1.356248
H	3.175307	-2.384375	-2.460597

H	3.785209	-3.186157	-4.730337
H	5.008477	-1.689789	-6.293594
H	5.611426	0.615564	-5.574544
H	4.987229	1.411636	-3.314241
H	0.730547	1.103246	1.349688
H	1.111767	3.393003	0.921483
H	-0.182602	3.115465	-0.264743
H	1.787381	5.637429	0.509996
H	2.711895	7.365178	-1.004308
H	2.996892	6.876220	-3.426464
H	2.340786	4.647927	-4.319176
H	1.400688	2.927630	-2.797725
H	0.070823	-0.150765	-1.353564
H	-1.446164	1.413435	1.586022
H	-2.733485	0.360406	0.987820
H	-3.147994	2.889737	2.587118
H	-4.634100	4.837796	2.250040
H	-5.304959	5.512726	-0.050465
H	-4.474136	4.217310	-2.003403
H	-2.988291	2.258585	-1.656971
H	-0.961431	-1.030000	1.400990
H	-1.151251	-2.701032	-1.085226
H	-2.280182	-1.331843	-0.917577
H	-1.204837	-3.619807	1.827695
H	-1.464049	-4.516834	0.324163
H	-1.807122	-5.220901	3.460585
H	-3.534549	-6.617763	4.558373
H	-5.778138	-6.874954	3.513894
H	-6.275138	-5.726916	1.363859
H	-4.532133	-4.341495	0.262812

Man2α

C	-3.205153	-4.860745	-3.295568
C	-3.379859	-3.820021	-2.190885
S	-2.624626	-2.219702	-2.700509
C	-2.873959	-1.280971	-1.113691
C	-2.455595	0.192701	-1.300507
O	-3.247202	1.073509	-0.516516
C	-3.113130	1.042651	0.906122
C	-4.108525	2.008774	1.506445
C	-4.774062	1.687504	2.693782
C	-5.655632	2.594852	3.284206
C	-5.888581	3.833033	2.685060
C	-5.232806	4.158806	1.495570
C	-4.346645	3.254340	0.911831
C	-0.918726	0.361939	-1.139459
O	-0.542729	1.707340	-0.893518
C	-0.767014	2.608034	-1.977326
C	-0.181582	3.952628	-1.619406
C	0.679737	4.617550	-2.497545
C	1.199843	5.872280	-2.170051
C	0.869458	6.469987	-0.954269
C	0.012925	5.809732	-0.068844
C	-0.511030	4.561787	-0.400786
C	-0.259136	-0.478676	-0.031488
O	1.132547	-0.556658	-0.313421
C	1.975478	0.199493	0.571745
C	3.334344	0.343942	-0.064968
C	4.420601	-0.425935	0.362574
C	5.670036	-0.289224	-0.247568
C	5.841647	0.617321	-1.293824
C	4.760237	1.389552	-1.728413
C	3.515641	1.252509	-1.116633
C	-0.827029	-1.904679	0.010125
C	-0.494153	-2.677730	1.299230
O	0.872040	-2.744435	1.642530
C	1.665208	-3.563157	0.792952
C	2.973615	-3.896648	1.474943
C	4.076054	-4.280408	0.700703
C	5.279104	-4.641512	1.306991
C	5.398346	-4.610388	2.698556
C	4.307047	-4.217602	3.475255
C	3.099500	-3.867136	2.868357
O	-2.262049	-1.888262	0.004987
H	-3.662617	-5.808969	-2.990827
H	-3.684162	-4.538327	-4.226461
H	-2.146150	-5.045893	-3.504429
H	-2.896171	-4.141087	-1.263863
H	-4.442345	-3.652817	-1.980199
H	-3.944309	-1.304068	-0.892657
H	-2.724281	0.483860	-2.321103
H	-2.092999	1.342401	1.181578
H	-3.284021	0.031435	1.295680
H	-4.603604	0.719236	3.159607
H	-6.166432	2.329762	4.206240
H	-6.579364	4.538473	3.139367
H	-5.412484	5.119796	1.020626
H	-3.845317	3.502525	-0.018296
H	-0.464793	0.014797	-2.082424
H	-1.847146	2.699462	-2.162790
H	-0.300177	2.218052	-2.895259
H	0.946414	4.152173	-3.443979
H	1.868552	6.376754	-2.862443
H	1.277296	7.443614	-0.695794
H	-0.249162	6.269437	0.880375
H	-1.174743	4.047720	0.288288
H	-0.426195	0.001192	0.940801
H	2.047894	-0.321258	1.535496
H	1.530553	1.188850	0.733355
H	4.289597	-1.137126	1.174184
H	6.506137	-0.892976	0.095558
H	6.813959	0.725902	-1.768012
H	4.890381	2.101248	-2.539993
H	2.672258	1.855132	-1.444929
H	-0.464511	-2.442444	-0.875398
H	-0.926179	-3.688007	1.210190
H	-0.989820	-2.173978	2.136113
H	1.857846	-3.050333	-0.158597
H	1.114177	-4.493440	0.565802
H	3.992859	-4.290886	-0.384494
H	6.125335	-4.937905	0.692300
H	6.336530	-4.885209	3.173127
H	4.393576	-4.184973	4.558822
H	2.249436	-3.558498	3.467671

Man3α

O	4.229743	0.144155	-0.464573
P	3.699425	-1.124110	-1.014955
O	4.310050	-1.624067	-2.415859
C	5.688854	-1.715848	-2.668101
C	6.161354	-2.938294	-3.138586
C	7.511978	-3.058722	-3.466879

C	8.371731	-1.967826	-3.324429
C	7.875787	-0.750535	-2.853797
C	6.526012	-0.611050	-2.526210
O	3.906432	-2.458217	-0.120260
C	3.379318	-2.590750	1.167835
C	3.597155	-1.629080	2.154906
C	3.073682	-1.848328	3.431375
C	2.362747	-3.015136	3.720575
C	2.170650	-3.973408	2.721662
C	2.673896	-3.762831	1.437019
O	2.129481	-1.158847	-1.312000
C	1.371568	0.064332	-1.638143
C	0.967379	0.757397	-0.332629
O	0.297713	1.948802	-0.720809
C	0.589135	3.113932	0.068461
C	-0.564617	4.074955	-0.063980
C	-0.472405	5.214041	-0.868998
C	-1.560518	6.081065	-0.999073
C	-2.753139	5.809264	-0.327372
C	-2.854558	4.669677	0.476556
C	-1.766934	3.807952	0.606801
C	0.030659	-0.151203	0.477316
O	-0.357131	0.580407	1.632383
C	-0.771359	-0.193997	2.761608
C	-0.713791	0.670991	3.997720
C	-1.785563	0.703215	4.894968
C	-1.717875	1.474445	6.057747
C	-0.578826	2.233182	6.327094
C	0.493399	2.214525	5.430621
C	0.427250	1.435052	4.277465
C	-1.167310	-0.528151	-0.409369
O	-2.104166	-1.398299	0.234372
C	-1.715453	-2.749746	0.495588
C	-2.658081	-3.331626	1.523642
C	-2.167329	-4.106924	2.579295
C	-3.040289	-4.677389	3.508152
C	-4.414800	-4.470103	3.392941
C	-4.911963	-3.690097	2.345409
C	-4.039966	-3.126702	1.415281
C	-0.713370	-1.097554	-1.779150
C	-1.860664	-1.252855	-2.803342
O	-2.088463	-0.145502	-3.643323
C	-2.597879	1.031989	-3.026343
C	-3.164971	1.950271	-4.085476
C	-3.102464	3.336958	-3.908862
C	-3.664405	4.197836	-4.852666
C	-4.286220	3.680296	-5.989979
C	-4.342358	2.297363	-6.176398
C	-3.788693	1.437178	-5.228227
O	0.294287	-0.293405	-2.412580
H	5.472507	-3.770206	-3.243061
H	7.890248	-4.009169	-3.832980
H	9.422879	-2.065840	-3.579839
H	8.539363	0.102550	-2.742504
H	6.126470	0.325831	-2.155530
H	4.163916	-0.734759	1.921013
H	3.231495	-1.102423	4.205391
H	1.968075	-3.179557	4.718924
H	1.632286	-4.891492	2.940447
H	2.536830	-4.492065	0.645100
H	1.993381	0.721342	-2.248052
H	1.868985	0.983477	0.246869
H	1.523292	3.572509	-0.287627
H	0.720207	2.819967	1.114607
H	0.454963	5.423487	-1.397587
H	-1.475614	6.966366	-1.624129
H	-3.600637	6.482518	-0.427866
H	-3.780749	4.455995	1.003672
H	-1.835923	2.918600	1.228119
H	0.592756	-1.047112	0.777099
H	-0.091452	-1.055535	2.873015
H	-1.782365	-0.587223	2.609962
H	-2.679859	0.121466	4.681921
H	-2.558382	1.487690	6.746915
H	-0.526881	2.839889	7.227235
H	1.381202	2.809264	5.631539
H	1.256775	1.423655	3.576212
H	-1.734039	0.391105	-0.559217
H	-0.681630	-2.813921	0.858702
H	-1.768266	-3.340554	-0.432003
H	-1.094802	-4.259023	2.679418
H	-2.645004	-5.275839	4.325082
H	-5.095424	-4.910227	4.116787
H	-5.981483	-3.521189	2.252650
H	-4.423258	-2.511252	0.606707
H	-0.287973	-2.095090	-1.606370
H	-2.771012	-1.532018	-2.247678
H	-1.611080	-2.068934	-3.488120
H	-1.807819	1.553523	-2.470442
H	-3.389727	0.757904	-2.305504
H	-2.606929	3.745652	-3.031069
H	-3.606307	5.272905	-4.702828
H	-4.718061	4.349395	-6.729957
H	-4.817197	1.887241	-7.064420
H	-3.817878	0.362419	-5.375028

Fuc1α

C	-3.932972	-2.355837	-0.355763
C	-2.426409	-2.232676	-0.513800
C	-1.955226	-0.811231	-0.882622
O	-2.678614	0.243337	-0.266256
C	-2.472078	0.512369	1.128800
C	-3.468087	1.562502	1.547142
C	-4.622032	1.218482	2.257750
C	-5.549730	2.194863	2.626507
C	-5.329933	3.529433	2.283278
C	-4.179244	3.882621	1.573481
C	-3.255165	2.905052	1.208439
C	-0.404692	-0.694847	-0.761371
O	0.038138	0.651533	-0.761159
C	-0.157306	1.349052	-1.995131
C	0.546646	2.679776	-1.908828
C	-0.181249	3.873616	-1.892147
C	0.473265	5.104345	-1.797686
C	1.865277	5.149839	-1.712816
C	2.600648	3.961779	-1.727757
C	1.945027	2.735829	-1.828538
C	0.164328	-1.361128	0.500546
O	1.576579	-1.512254	0.444383
C	2.324885	-0.420208	0.999205
C	3.796471	-0.629600	0.738400
C	4.739734	-0.121503	1.639782

C	6.105931	-0.250957	1.386526
C	6.544614	-0.902585	0.233043
C	5.609457	-1.419396	-0.665383
C	4.243288	-1.279292	-0.418948
C	-0.443826	-2.757012	0.697796
O	-0.038151	-3.643104	-0.372827
C	0.930371	-4.573453	-0.152698
C	0.583692	-5.861146	-0.922720
Cl	1.862780	-7.090149	-0.780424
Cl	-0.963807	-6.524178	-0.259454
Cl	0.345811	-5.451167	-2.671495
N	1.976325	-4.493509	0.544948
O	-1.829923	-2.702946	0.723351
H	-4.305983	-1.697220	0.432509
H	-4.429062	-2.076201	-1.291012
H	-4.197006	-3.389457	-0.113401
H	-2.090879	-2.905420	-1.312915
H	-2.210029	-0.683567	-1.942194
H	-1.452403	0.887936	1.273981
H	-2.602916	-0.398747	1.724966
H	-4.792881	0.178449	2.527795
H	-6.440817	1.912915	3.182619
H	-6.047900	4.292540	2.571451
H	-4.001865	4.921723	1.308926
H	-2.356595	3.179279	0.660125
H	0.029238	-1.232982	-1.620132
H	-1.228044	1.497164	-2.179682
H	0.254237	0.741735	-2.817824
H	-1.266984	3.838528	-1.954256
H	-0.104540	6.024203	-1.787518
H	2.377234	6.105647	-1.636704
H	3.685782	3.991685	-1.663716
H	2.520837	1.813453	-1.838937
H	-0.105478	-0.777188	1.388697
H	2.136358	-0.353198	2.081476
H	1.976836	0.513395	0.543187
H	4.403484	0.376537	2.546451
H	6.825789	0.147592	2.096581
H	7.607487	-1.012497	0.040142
H	5.943146	-1.936169	-1.561587
H	3.516640	-1.691197	-1.111355
H	-0.115255	-3.188827	1.647694
H	2.086744	-3.546362	0.919081

Nucleophile

MeOH

C	-0.123460	0.000000	0.337185
O	-0.192115	0.000000	-1.080034
H	-1.150831	0.000000	0.702318
H	0.380344	-0.892068	0.734004
H	0.380344	0.892068	0.734004
H	0.705719	0.000000	-1.427477

EtOH

C	-0.375357	0.000000	-0.947460
C	0.431171	0.000000	0.336500
O	-0.486171	0.000000	1.427114
H	0.282413	0.000000	-1.821036
H	-1.015279	-0.884228	-0.995047
H	-1.015279	0.884228	-0.995047
H	1.081535	0.886982	0.372524
H	1.081535	-0.886982	0.372524
H	0.015432	0.000000	2.249929

iPrOH

C	0.140269	1.210556	-0.419612
C	-0.353549	-0.056033	0.278619
C	0.141715	-1.327199	-0.393854
O	0.111849	-0.111789	1.631087
H	-0.220652	2.109123	0.093000
H	1.233343	1.240333	-0.421319
H	-0.211140	1.260817	-1.454716
H	-1.455253	-0.058436	0.272799
H	1.234927	-1.351022	-0.399691
H	-0.213050	-2.205179	0.149759
H	-0.213517	-1.390052	-1.425898
H	-0.194942	0.678880	2.089827

tBuOH

C	-0.604577	1.263356	0.389498
C	0.000103	0.000000	-0.236800
C	1.522181	0.000000	-0.109663
C	-0.604576	-1.263356	0.389498
O	-0.247615	0.000000	-1.654556
H	-1.692406	1.280271	0.258643
H	-0.402605	1.320641	1.463094
H	-0.194788	2.156206	-0.088378
H	1.942472	-0.883441	-0.595806
H	1.942472	0.883438	-0.595811
H	1.831480	0.000003	0.938769
H	-0.194792	-2.156205	-0.088384
H	-0.402597	-1.320645	1.463092
H	-1.692406	-1.280268	0.258650
H	-1.202348	-0.000001	-1.791848

2F-EtOH

O	-1.391303	0.035174	0.788780
C	0.016033	0.072237	0.724627
C	0.527564	0.105730	-0.702846
F	0.001785	-0.976422	-1.379559
F	0.110060	1.225760	-1.355415
H	-1.696133	-0.697872	0.239942
H	0.476832	-0.791923	1.226510
H	0.338269	0.978443	1.241002
H	1.616893	0.048873	-0.783042

3F-EtOH

O	-1.413620	-0.081686	0.798024
C	-0.009797	-0.093973	0.712859
C	0.475392	-0.091233	-0.727796
F	1.820442	-0.012633	-0.780680
F	0.099707	-1.185048	-1.408305
F	-0.023976	0.983983	-1.390609
H	-1.741103	0.727972	0.388082
H	0.337100	-1.010035	1.191399
H	0.455854	0.762654	1.217025

ManOH

O	1.492357	1.835701	3.143047
C	0.593373	2.868216	2.751149
C	0.277889	2.732761	1.273129

H	-0.580895	3.381213	1.056443
O	1.420493	3.196044	0.537701
C	1.282847	3.146222	-0.862565
H	2.263224	3.451671	-1.244117
O	0.269453	3.998065	-1.345290
C	0.556825	5.382008	-1.194745
C	0.949036	1.734730	-1.363148
H	0.762425	1.791480	-2.441741
O	2.079731	0.920847	-1.094327
C	2.412058	-0.005249	-2.117257
C	3.594671	-0.843758	-1.695997
C	4.349568	-0.541777	-0.561435
C	5.443918	-1.334603	-0.215043
C	5.795532	-2.431828	-0.996102
C	5.042652	-2.738767	-2.129349
C	3.949062	-1.950960	-2.473209
C	-0.095247	1.295496	0.873528
H	0.713492	0.624480	1.166605
O	-1.306536	0.960501	1.544625
C	-1.251392	-0.234382	2.313267
C	-2.545724	-0.432406	3.066892
C	-2.623019	-1.436940	4.037695
C	-3.807893	-1.668304	4.729431
C	-4.935826	-0.893108	4.463305
C	-4.863768	0.112956	3.504012
C	-3.676829	0.342132	2.808517
C	-0.295606	1.202549	-0.635551
H	-1.155571	1.824255	-0.908711
O	-0.543552	-0.156832	-0.986279
C	-1.658936	-0.356830	-1.837230
C	-1.779477	-1.814917	-2.217102
C	-0.913278	-2.783566	-1.708868
C	-1.055554	-4.121182	-2.079780
C	-2.062415	-4.505134	-2.960115
C	-2.932652	-3.541435	-3.470342
C	-2.789595	-2.207704	-3.101946
H	1.770258	1.998446	4.049990
H	1.034749	3.859914	2.910485
H	-0.345964	2.806595	3.313330
H	-0.284686	5.924867	-1.623568
H	1.473449	5.651294	-1.735235
H	0.672848	5.658759	-0.142672
H	2.656618	0.542539	-3.041782
H	1.555476	-0.651437	-2.334980
H	4.069292	0.306503	0.050373
H	6.020226	-1.092799	0.672947
H	6.645850	-3.048027	-0.722317
H	5.302506	-3.597498	-2.740490
H	3.359184	-2.204205	-3.351210
H	-0.410248	-0.181241	3.016879
H	-1.074505	-1.090088	1.649178
H	-1.748249	-2.045595	4.253014
H	-3.851094	-2.453048	5.478369
H	-5.861240	-1.072214	5.001572
H	-5.735790	0.724605	3.291789
H	-3.619385	1.125389	2.062877
H	-1.559107	0.251713	-2.748916
H	-2.575480	-0.025993	-1.327212
H	-0.128883	-2.486970	-1.024259
H	-0.373416	-4.863085	-1.676014
H	-2.171717	-5.546282	-3.247472
H	-3.723052	-3.829761	-4.157215
H	-3.472484	-1.464030	-3.506555

GlcOH

C	-1.507409	1.651570	-3.686122
O	-0.847635	1.099582	-2.549435
C	-1.498969	1.374761	-1.342768
C	-0.624336	0.876284	-0.178540
O	0.700110	1.380453	-0.205571
C	0.927381	2.624625	0.445667
C	0.744134	3.843780	-0.438234
C	0.344242	5.060654	0.119953
C	0.235491	6.204664	-0.668767
C	0.519403	6.141023	-2.031305
C	0.913415	4.928847	-2.596426
C	1.028650	3.787936	-1.805800
C	-0.543897	-0.657140	-0.176233
O	0.046277	-1.150811	1.020917
C	1.470832	-1.211485	1.076466
C	2.048762	-0.323948	2.158727
C	1.270989	0.115717	3.230648
C	1.830702	0.902052	4.236787
C	3.178528	1.250447	4.189086
C	3.963273	0.810168	3.124485
C	3.400046	0.031371	2.116551
C	-1.941788	-1.286943	-0.266376
O	-1.887982	-2.684938	-0.518356
C	-1.689933	-3.552727	0.595837
C	-0.383487	-4.313874	0.526206
C	0.244545	-4.562978	-0.695501
C	1.417110	-5.314802	-0.746919
C	1.973353	-5.831698	0.421434
C	1.350271	-5.589064	1.644578
C	0.180803	-4.834072	1.694155
C	-2.750094	-0.658168	-1.410446
C	-4.187811	-1.129296	-1.454291
O	-4.734985	-0.737359	-2.708833
O	-2.775322	0.765860	-1.259559
H	-1.576138	2.741783	-3.597007
H	-0.897237	1.397782	-4.552055
H	-2.510244	1.233953	-3.804444
H	-1.702289	2.447687	-1.253465
H	-1.120602	1.189592	0.749621
H	1.962140	2.573181	0.796982
H	0.292734	2.706274	1.337046
H	0.113213	5.115685	1.181001
H	-0.077802	7.142575	-0.220776
H	0.429487	7.028269	-2.650230
H	1.133568	4.871760	-3.658297
H	1.314922	2.839321	-2.244004
H	0.040292	-0.979154	-1.045030
H	1.899714	-0.941922	0.109001
H	1.735481	-2.255966	1.278587
H	0.222374	-0.156023	3.262341
H	1.211657	1.240050	5.062251
H	3.614928	1.859847	4.974348
H	5.014147	1.078446	3.076270
H	4.016122	-0.299956	1.284491
H	-2.462617	-1.099258	0.683892
H	-2.522439	-4.269028	0.586795
H	-1.745385	-2.993431	1.532495

H	-0.188187	-4.156296	-1.602417
H	1.895521	-5.498757	-1.704175
H	2.886708	-6.416782	0.380639
H	1.779778	-5.980245	2.561776
H	-0.293411	-4.641743	2.653175
H	-2.275756	-0.925378	-2.360062
H	-4.735843	-0.670900	-0.619424
H	-4.201505	-2.218445	-1.337718
H	-5.668000	-0.973409	-2.723622

Acid Catalyst (Conjugate Base)

TfO^-

S	0.270379	0.882206	0.414833
O	1.696679	0.975628	0.055299
O	-0.011469	0.657497	1.843955
O	-0.620780	1.838176	-0.266618
C	-0.232525	-0.758053	-0.356693
F	-0.061167	-0.759506	-1.696659
F	0.490875	-1.788616	0.133131
F	-1.531991	-1.047333	-0.127248

FSO_3^-

S	0.000031	0.000000	-0.156998
F	0.000161	0.000000	1.535338
O	-1.434075	0.000000	-0.459598
O	0.716941	1.242035	-0.459372
O	0.716941	-1.242035	-0.459372

MsO^-

O	-1.441269	-0.000000	-1.271187
S	0.000144	0.000000	-0.909726
O	0.720644	1.248188	-1.271986
C	0.000095	0.000000	0.917589
O	0.720644	-1.248188	-1.271986
H	1.032367	0.000000	1.269581
H	-0.516313	0.893960	1.268858
H	-0.516313	-0.893960	1.268858

Tf_2N^-

S	0.825785	1.172321	0.099844
O	1.791367	1.712995	1.051931
O	1.246052	0.921706	-1.277113
N	-0.000000	-0.000000	0.837770
S	-0.825785	-1.172321	0.099843
O	-1.791367	-1.712995	1.051930
O	-1.246052	-0.921706	-1.277113
C	0.456422	-2.545965	-0.044252
F	1.506164	-2.168580	-0.780452
F	0.900597	-2.920826	1.162207
F	-0.102933	-3.619846	-0.631049
C	-0.456422	2.545966	-0.044252
F	-0.900597	2.920826	1.162207
F	0.102933	3.619846	-0.631049
F	-1.506164	2.168580	-0.780452

$C_3F_6S_2O_4N^-$

O	1.670824	2.476758	0.912300
S	1.256195	1.408058	0.011251
N	1.700913	0.000000	0.672816
S	1.256195	-1.408058	0.011251
C	-0.627469	-1.309374	0.188958
F	-1.194968	-2.352831	-0.461704
F	-0.946009	-1.409690	1.498884
C	-1.246122	0.000000	-0.365166
F	-1.151840	0.000000	-1.710899
F	-2.577352	0.000000	-0.047949
C	-0.627469	1.309374	0.188958
F	-1.194968	2.352831	-0.461704
F	-0.946009	1.409690	1.498884
O	1.478627	-1.563956	-1.424090
O	1.670824	-2.476758	0.912300
O	1.478627	1.563956	-1.424090

Solvent

DCM

Cl	-1.496181	0.000000	-0.834294
C	0.000000	0.000000	0.151397
H	0.000000	0.898069	0.758595
H	0.000000	-0.898069	0.758595
Cl	1.496181	0.000000	-0.834294

$CHCl_3$

Cl	1.00141	-0.08214	0.04810
C	2.77273	-0.07859	0.08300
H	3.13708	-0.18292	-0.94223
Cl	3.37971	1.45190	0.73720
Cl	3.37970	-1.44594	1.03208

Toluene

C	0.000000	0.006865	-2.229297
C	0.000000	-0.015533	-0.719333
C	-1.200404	-0.012617	-0.000802
C	-1.203309	-0.000020	1.392028
C	0.000000	0.007441	2.094572
C	1.203309	-0.000020	1.392028
C	1.200404	-0.012617	-0.000802
H	0.000000	1.034698	-2.608759
H	0.882894	-0.489118	-2.639973
H	-0.882894	-0.489118	-2.639973
H	-2.144384	-0.022278	-0.539169
H	-2.146761	0.000062	1.929350
H	0.000000	0.014470	3.179948
H	2.146761	0.000062	1.929350
H	2.144384	-0.022278	-0.539169

tert-butylbenzene

C	1.03252	-0.03386	-0.01238
C	2.57101	0.06688	-0.04893
C	3.11550	0.01937	1.39693
C	3.10889	-1.19200	-0.76695
C	2.96358	1.38576	-0.73824
C	2.73900	2.61951	-0.09606
C	3.07427	3.83282	-0.70426
C	3.63292	3.84322	-1.97696
C	3.85294	2.64083	-2.63949
C	3.52108	1.42675	-2.02805
H	0.70800	-0.96825	0.45996
H	0.61026	-0.00771	-1.02410
H	0.58212	0.79085	0.55167
H	2.89354	-0.94414	1.87106

H	2.67326	0.79070	2.03621
H	4.20302	0.15677	1.41386
H	2.84698	-2.10355	-0.21632
H	4.20137	-1.16603	-0.85537
H	2.68710	-1.29952	-1.77277
H	2.28849	2.65585	0.89306
H	2.89327	4.76853	-0.18214
H	3.89114	4.78488	-2.45299
H	4.28212	2.64268	-3.63826
H	3.70769	0.51820	-2.59331

Anisole

C	1.14586	-0.25066	-0.12817
O	2.51036	0.16600	-0.12800
C	3.10318	-0.03979	-1.34414
C	3.64814	1.07045	-1.98543
C	4.28725	0.91897	-3.21619
C	4.39849	-0.34745	-3.79176
C	3.87873	-1.46208	-3.13317
C	3.23489	-1.31113	-1.90371
H	0.69037	0.10010	0.80287
H	1.06070	-1.34156	-0.15110
H	0.59435	0.19702	-0.96285
H	3.56804	2.05337	-1.52957
H	4.70223	1.78600	-3.72243
H	4.90086	-0.46635	-4.74835
H	3.98401	-2.45069	-3.57284
H	2.86079	-2.18737	-1.38448

MTBE

C	-6.60414	-0.33061	0.05437
O	-5.19935	-0.47809	-0.08379
C	-4.73707	-1.47012	-1.01284
C	-5.18351	-2.87839	-0.60218
C	-5.18352	-1.15255	-2.44498
C	-3.20475	-1.39809	-0.94539
H	-6.78679	0.45666	0.79164
H	-7.06692	-1.24802	0.42589
H	-7.06696	-0.02002	-0.88540
H	-6.26487	-3.00736	-0.71760
H	-4.95143	-3.06928	0.45155
H	-4.69515	-3.64647	-1.21164
H	-6.26487	-1.27618	-2.56612
H	-4.69516	-1.81100	-3.17150
H	-4.95145	-0.11359	-2.70447
H	-2.85013	-1.59460	0.07321
H	-2.85014	-0.39456	-1.20824
H	-2.73237	-2.11922	-1.62071

ACN

C	-2.75374	-0.05943	0.04743
C	-1.22173	-0.05943	0.04743
N	-0.06173	-0.05943	0.04743
H	-3.11040	0.82272	0.53683
H	-3.11040	-0.92433	0.56669
H	-3.11040	-0.07667	-0.96123

α,α,α-trifluorotoluene

F	0.000000	-1.274939	2.657439
C	0.000000	-0.004958	2.185998
C	0.000000	0.030057	0.682890
C	1.209335	0.019966	-0.012490
C	1.206430	-0.002686	-1.404661
C	0.000000	-0.015252	-2.102057
C	-1.206430	-0.002686	-1.404661
C	-1.209335	0.019966	-0.012490
F	-1.087602	0.601128	2.710624
F	1.087602	0.601128	2.710624
H	2.146403	0.037469	0.531451
H	2.147810	-0.007540	-1.943578
H	0.000000	-0.031584	-3.186962
H	-2.147810	-0.007540	-1.943578
H	-2.146403	0.037469	0.531451

1,4-dioxane

O	-1.381564	0.000000	0.295914
C	-0.736678	-1.172467	-0.192174
C	0.736678	-1.172467	0.192174
O	1.381564	0.000000	-0.295914
C	0.736678	1.172467	0.192174
C	-0.736678	1.172467	-0.192174
H	-0.833016	-1.224953	-1.287302
H	-1.262200	-2.023135	0.248095
H	1.262200	-2.023135	-0.248095
H	0.833016	-1.224953	1.287302
H	1.262200	2.023135	-0.248095
H	0.833016	1.224953	1.287302
H	-0.833016	1.224953	-1.287302
H	-1.262200	2.023135	0.248095

References:

1 Chatterjee, S., Moon, S., Hentschel, F., Gilmore, K. & Seeberger, P. H. An Empirical Understanding of the Glycosylation Reaction. *J. Am. Chem. Soc.* **2018**, *140*, 11942-11953.

2 Cheng, J. M., Dangerfield, E. M., Timmer, M. S. & Stocker, B. L. A divergent approach to the synthesis of iGb3 sugar and lipid analogues via a lactosyl 2-azido-sphingosine intermediate. *Org. Biomol. Chem.* **2014**, *12*, 2729-2736.

3 Wang, Z., Zhou, L., El-Boubbou, K., Ye, X.-s. & Huang, X. Multi-component one-pot synthesis of the tumor-associated carbohydrate antigen Globo-H based on preactivation of thioglycosyl electrophiles. *J. Org. Chem.* **2007**, *72*, 6409-6420.

4 Wegmann, B. & Schmidt, R. R. Synthesis of the H-Disaccharide (2-O-α-L-Fucopyranosyl-D-Galactose) via the Trichloroacetimidate Method. *Carbohydr. Res.* **1988**, *184*, 254-261.

5 Durantie, E., Bucher, C. & Gilmour, R. Fluorine-Directed β-Galactosylation: Chemical Glycosylation Development by Molecular Editing. *Chem. Eur. J.* **2012**, *18*, 8208-8215.

6 Shie, C. R. *et al.* Cu (OTf) 2 as an Efficient and Dual-Purpose Catalyst in the Regioselective Reductive Ring Opening of Benzylidene Acetals. *Angew. Chem. Int. Ed.* **2005**, *44*, 1665-1668.

7 Higashi, K. & Susaki, H. A Novel Glycosidation Promoted by the Combination of Trimethylsilyl Halide and Zinc Triflate. *Chem. Pharm. Bull.* **1992**, *40*, 2019-2022.

8 Chang, G. X. & Lowary, T. L. A Glycosylation Protocol Based on Activation of Glycosyl 2-Pyridyl Sulfones with Samarium Triflate. *Org. Lett.* **2000**, *2*, 1505-1508.

9 He, H. & Zhu, X. Thioperoxide-Mediated Activation of Thioglycoside Electrophiles. *Org. Lett.* **2014**, *16*, 3102-3105.

10 Koshiba, M. *et al.* Catalytic Stereoselective Glycosidation with Glycosyl Diphenyl Phosphates: Rapid Construction of 1,2-cis-α-Glycosidic Linkages. *Chem. Asian J.* **2008**, *3*, 1664-1677.

11 Breiman, L. Random forests. *Mach. Learn.* **2001**, *45*, 5-32.

12 Friedman, J., Hastie, T., Rosset, S., Tibshirani, R. & Zhu, J. Discussion of Boosting Papers. *Ann. Stat.* **2004**, *32*, 102-107.

13 Eggensperger, K. *et al.* Towards an Empirical Foundation for Assessing Bayesian Optimization of Hyperparameters. In *NIPS workshop on Bayesian Optimization in Theory and Practice.* (2013).

NMR spectra

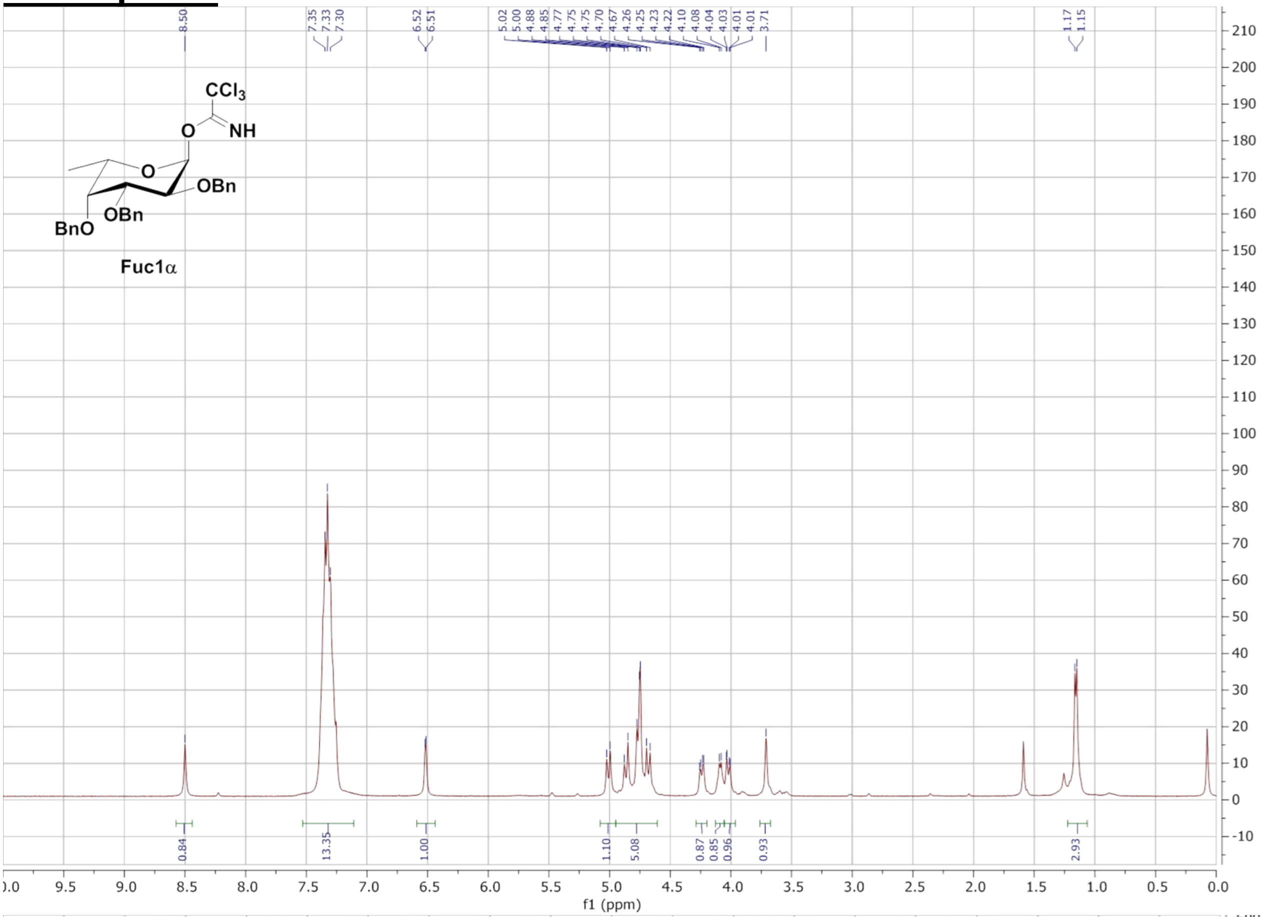

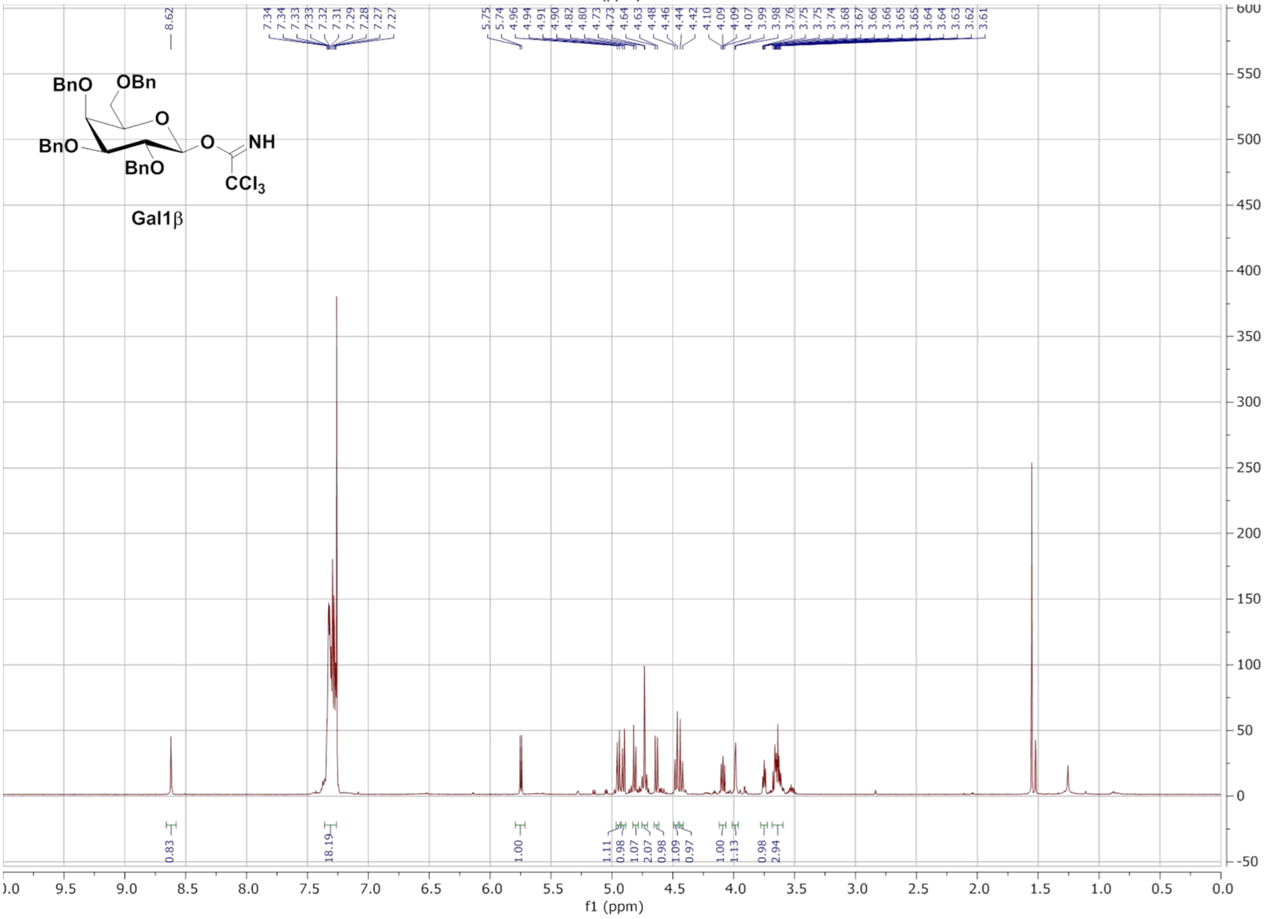

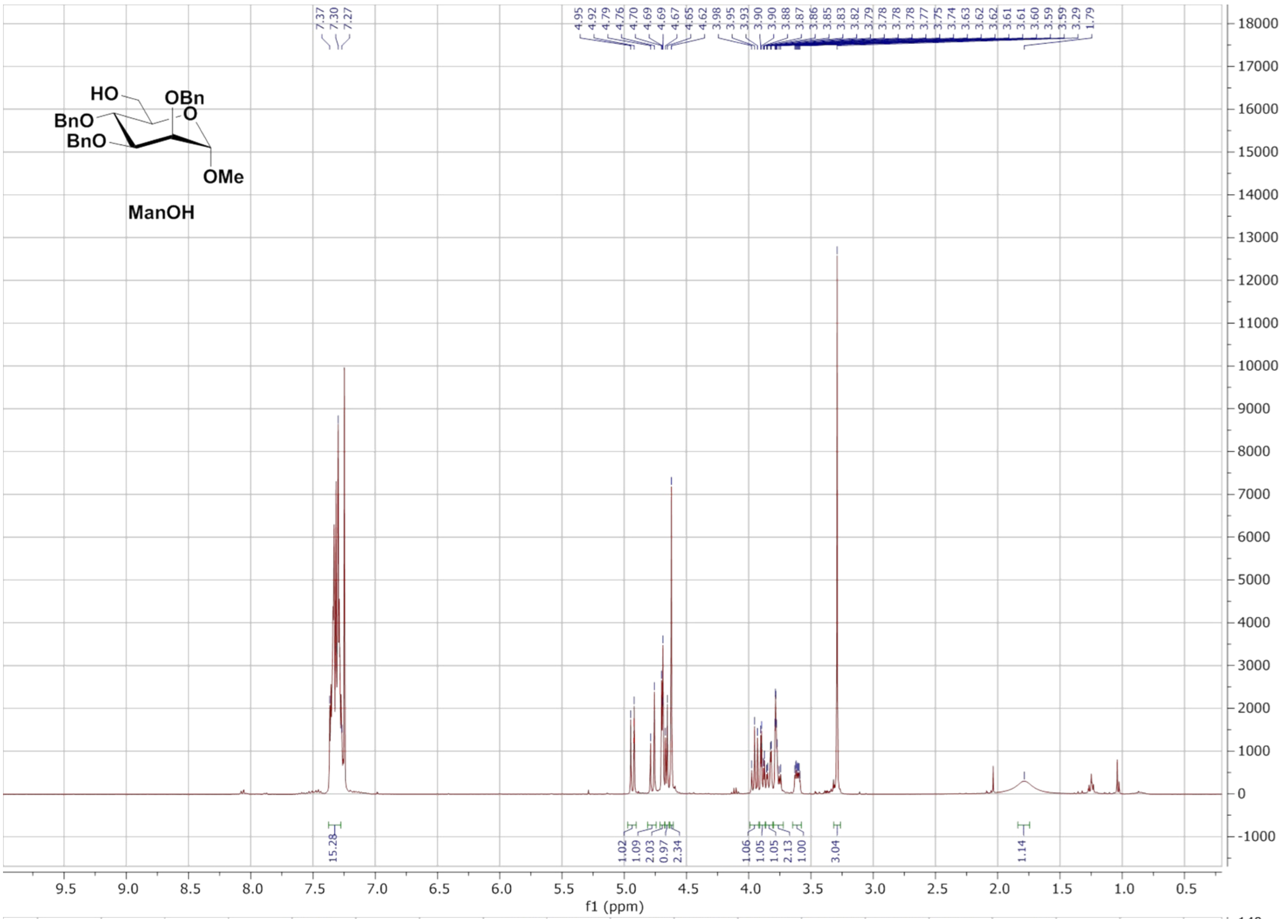
ManOH
f1 (ppm)

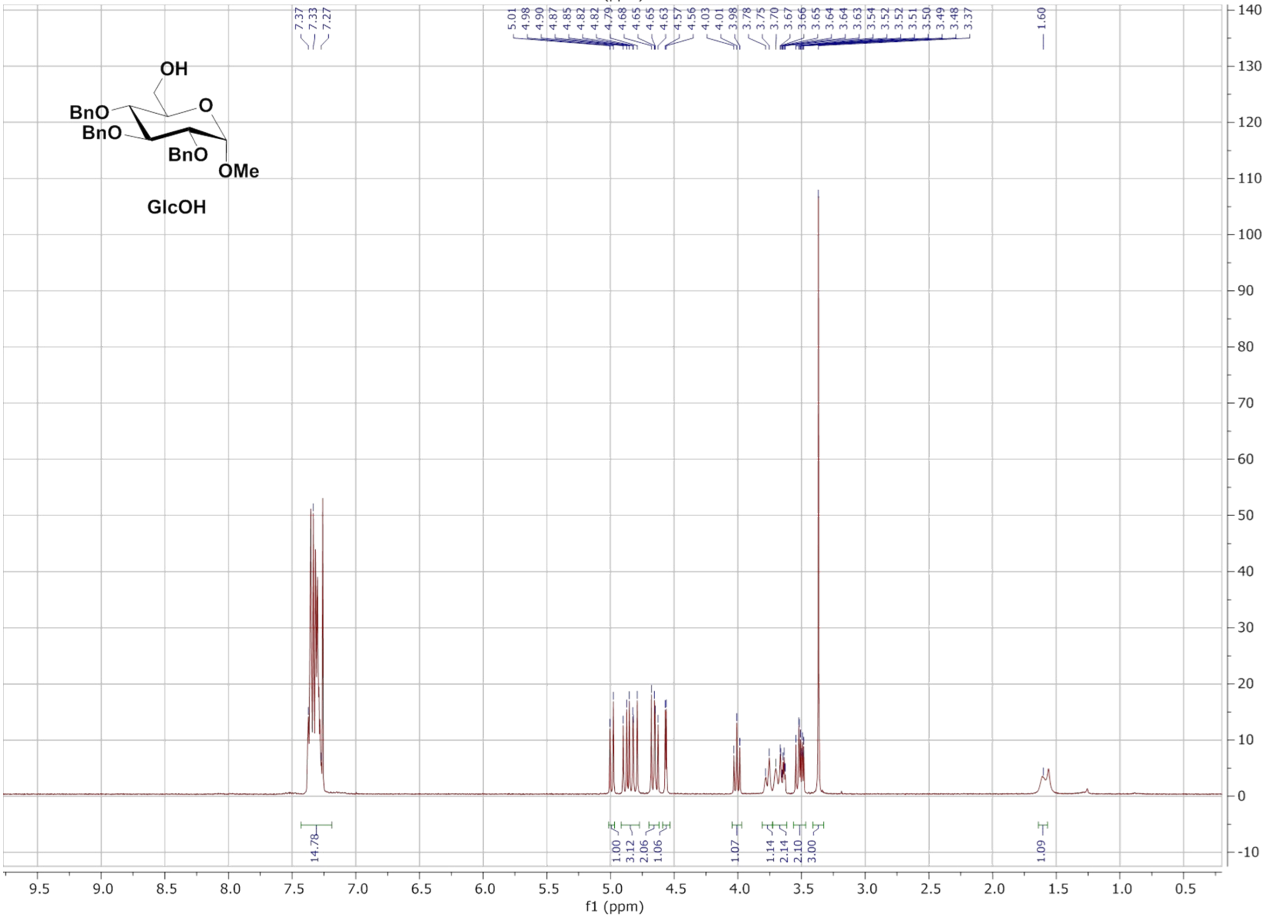
GlcOH
f1 (ppm)

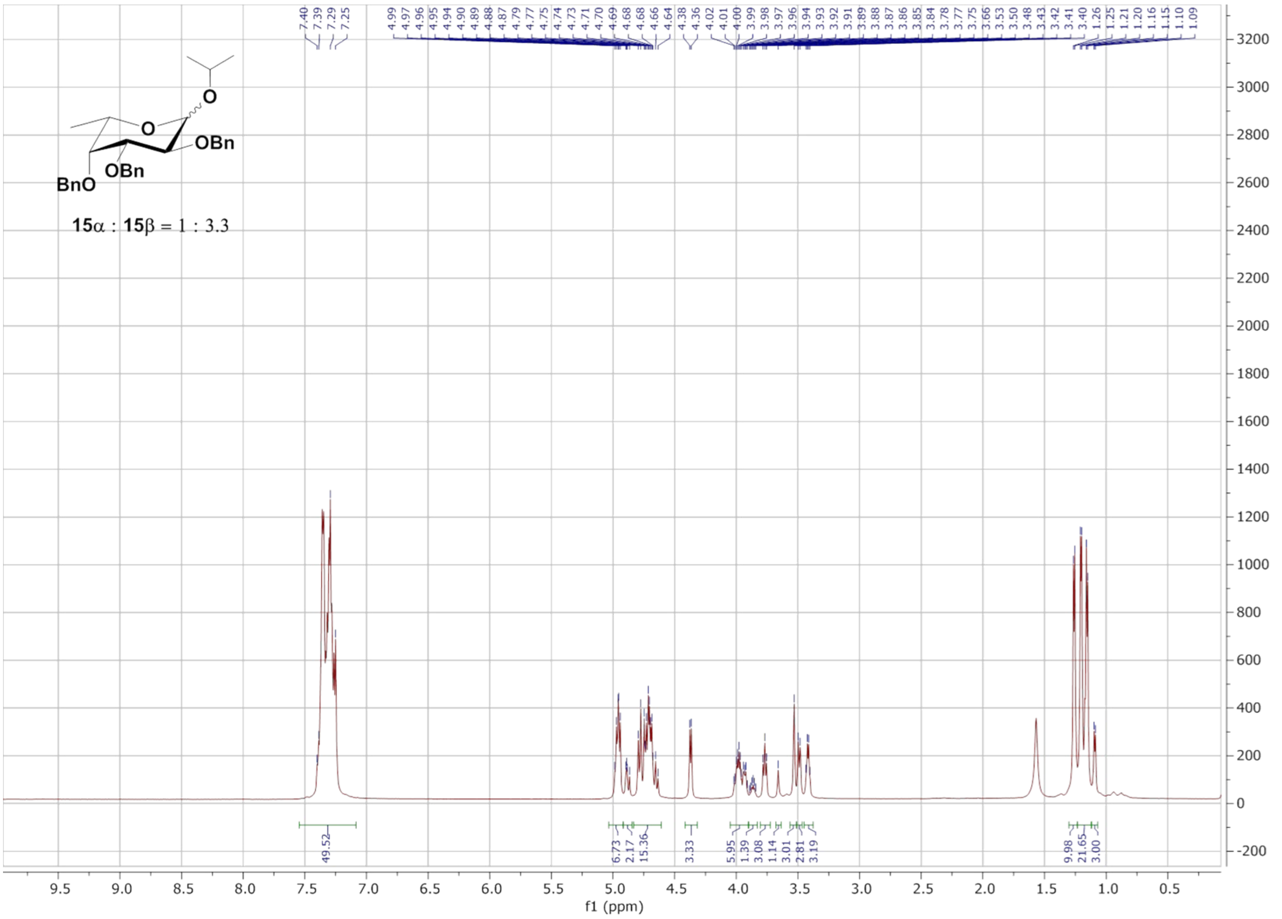
O
O
OBn
OBn
BnO
15α : 15β = 1 : 3.3
f1 (ppm)

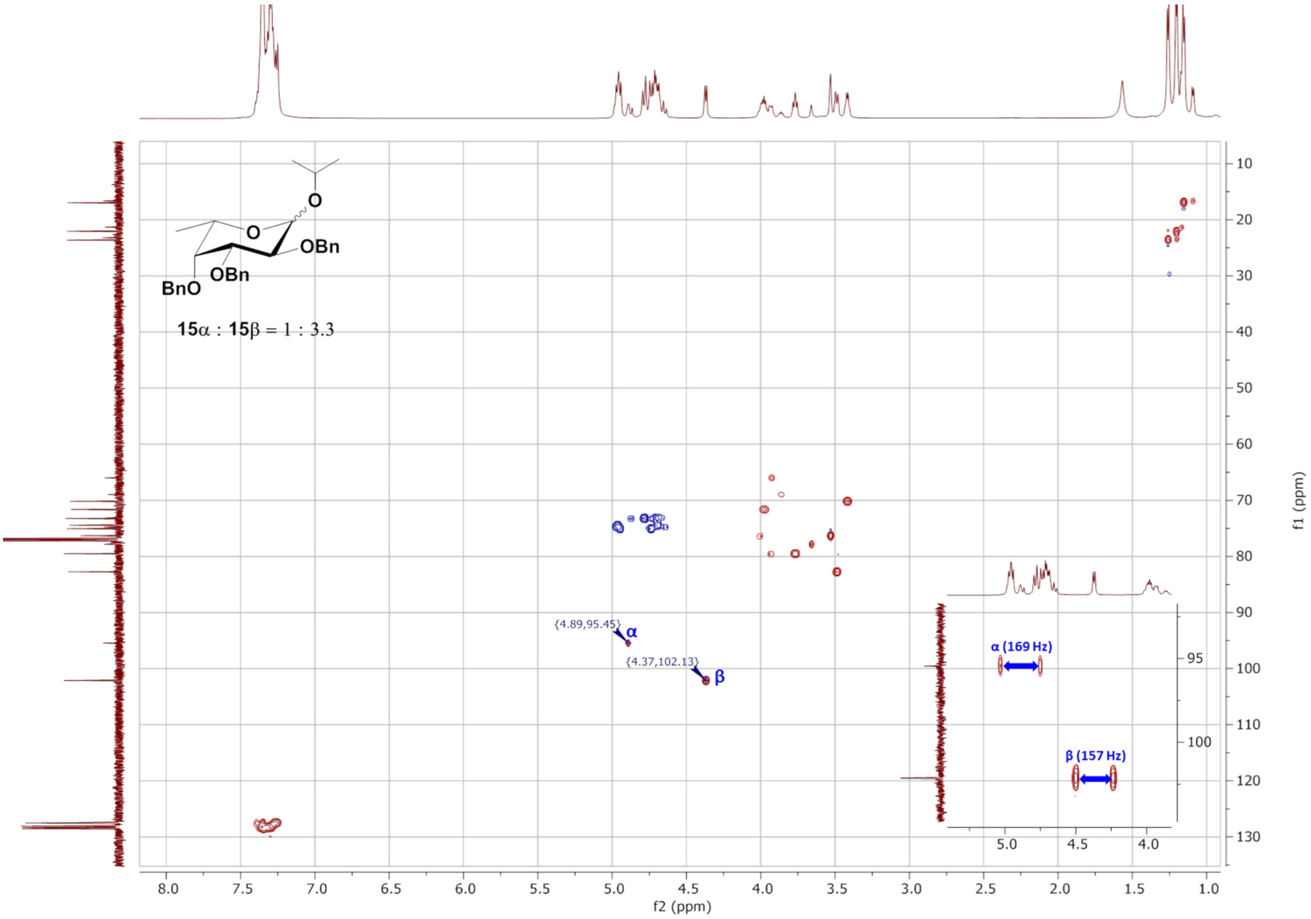
O
O
OBn
OBn
BnO
15α : 15β = 1 : 3.3
{4.89,95.45}
α
{4.37,102.13}
β
α (169 Hz)
β (157 Hz)
f2 (ppm)
f1 (ppm)

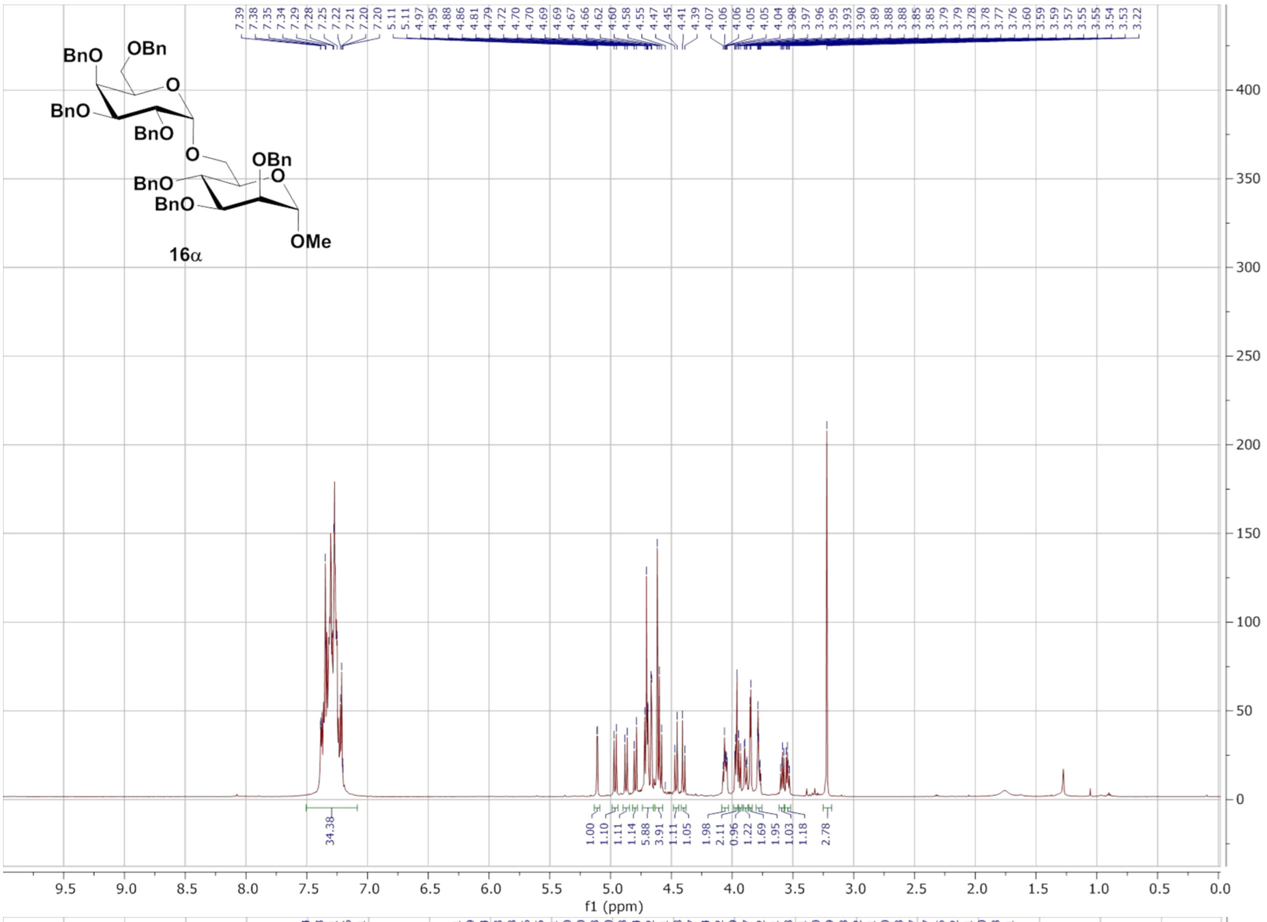
16α
f1 (ppm)

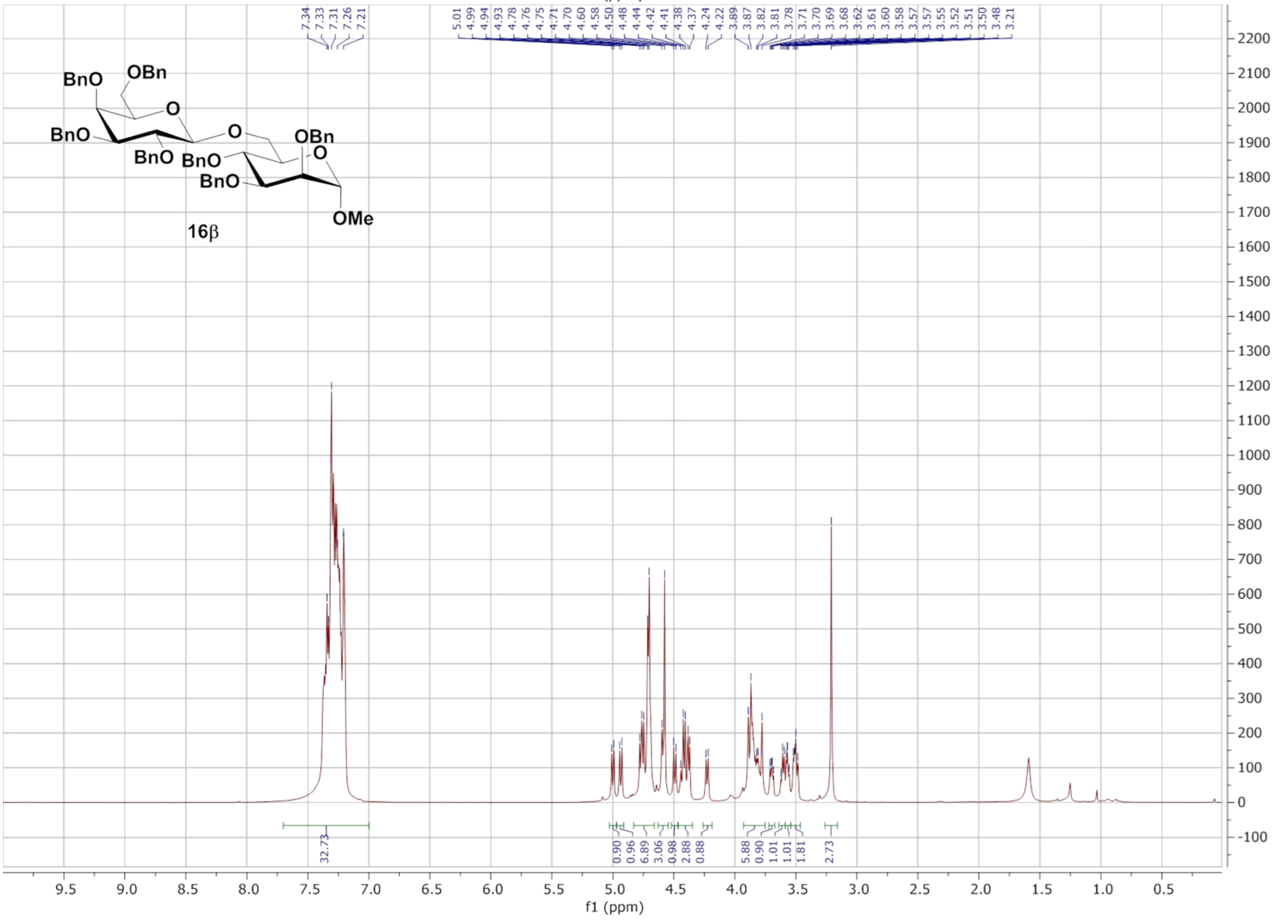
16β
f1 (ppm)

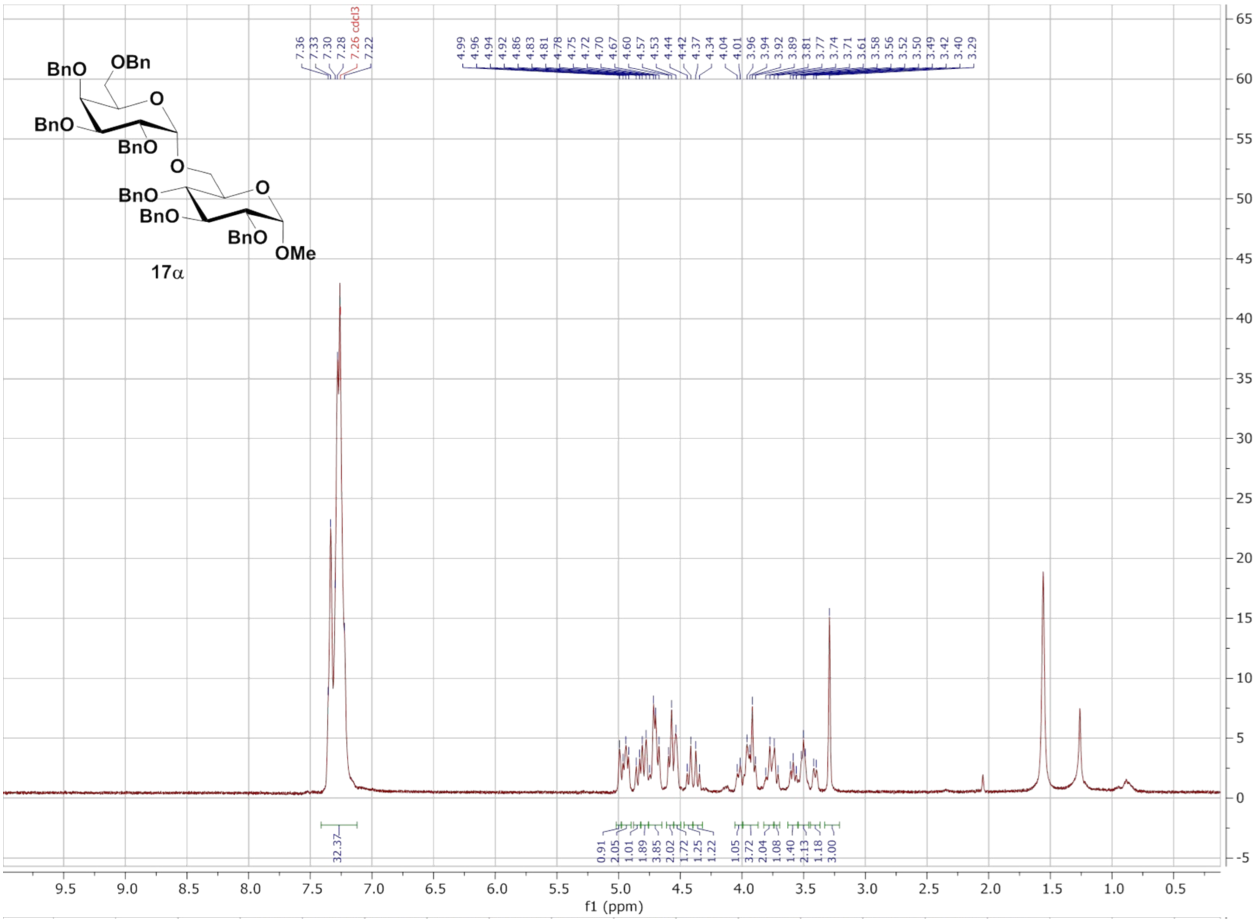
BnO
OBn
O
BnO
BnO
O
BnO
O
BnO
BnO
OMe
17α
7.26 cdcl3
32.37
f1 (ppm)

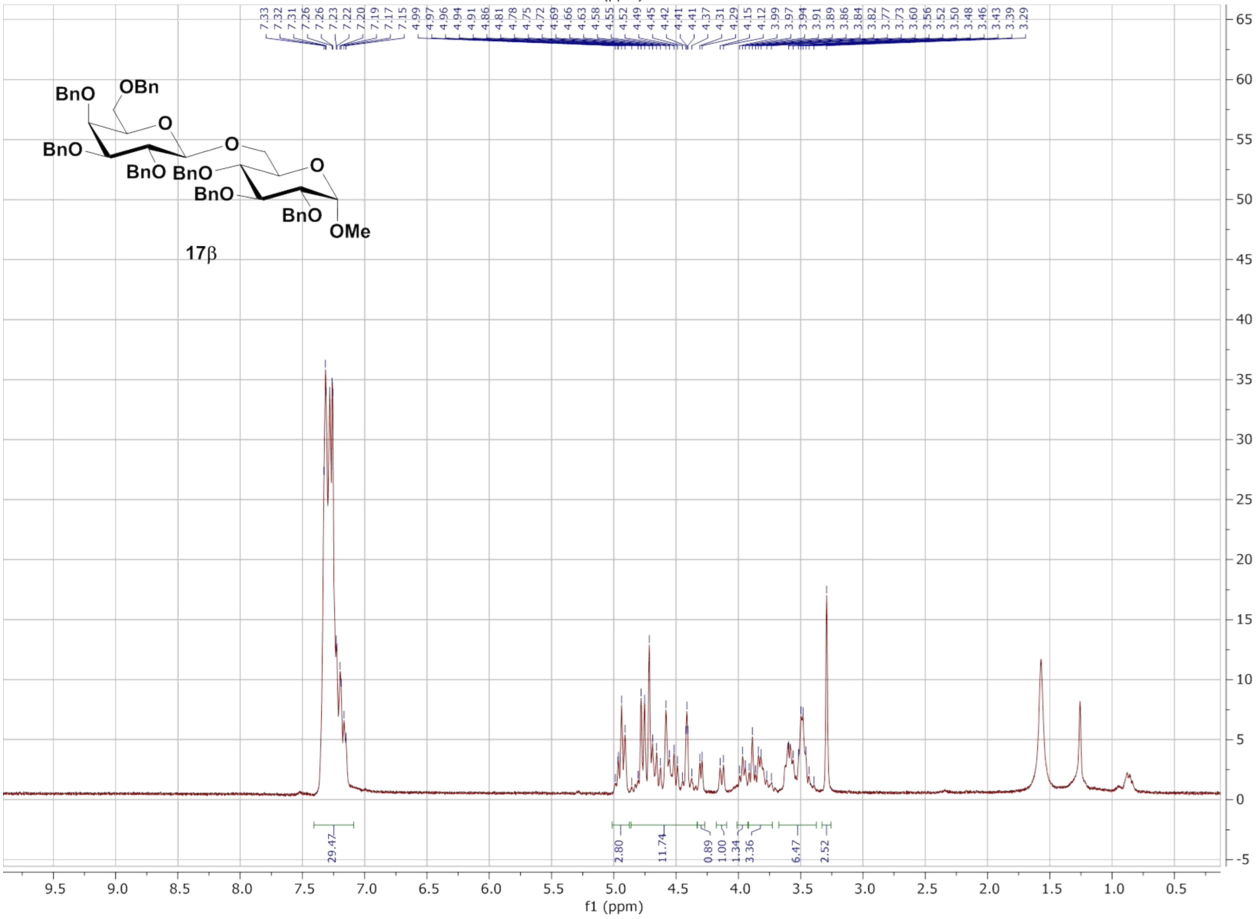
BnO
OBn
O
BnO
BnO
O
BnO
O
BnO
BnO
OMe
17β
29.47
11.74
f1 (ppm)
