# Supplementary material for: Predicting glycosylation stereoselectivity using machine learning
Source: Chem Sci. 2020 Dec 26;12(8):2931–9. doi: 10.1039/d0sc06222g (PMC8179398; doi:10.1039/d0sc06222g)
Supplement: SC-012-D0SC06222G-s002 [file SC-012-D0SC06222G-s002.pdf]

Electronic Supplementary Material (ESI) for Chemical Science.
This journal is © The Royal Society of Chemistry 2020

**Donor**	Glc1α	Glc1β	Glc2β	Gal1α	Gal1β	Gal2β	Man1α	Man2α	Fuc1α	Glc3α	Gal3α	Man3α
C1 shift	98.357	103.043	81.354	100.581	102.96	84.093	99.895	86.109	102.907	98.888	97.28	102.558
Binary C2	0	0	0	0	0	0	1	1	0	0	0	1
Binary C4	0	0	0	1	1	1	0	0	1	0	1	0
X1O2	56.83	-69.39	-50.24	54.79	-67.32	-66.38	171.39	146.95	57.59	60.71	54.86	177.51
O2O3	66.29	71.86	61.2	60.78	66.3	75.03	-57.35	-33.17	68.58	67.35	72.4	-60.52
O3O4	-64.53	-66.13	-70.91	58.87	55.74	31.43	-62.47	-73.49	32.84	-66.3	31.42	-63.04
O4C6	61.13	58.42	74.23	-56.73	-55.6	-40.86	60.28	76.45	-37.07	63.58	-39.21	62.22
H1-H2 j	3.39	7.8	9.5	3.46	7.9	9.19	2.51	0.97	3.68	3.09	3.83	2.95
H2-H3 j	9.6	8.67	9.41	10.25	9.72	7.88	3.42	5.15	9.28	9.45	8.78	3.06
H3-H4 j	9.14	8.85	8.42	2.84	3.35	5.31	9.43	8	5.02	8.9	5.19	9.26
H4-H5 j	9.65	9.74	9.54	0.98	1.1	1.93	9.68	9.36	2.24	9.51	2.21	9.54
HOMO	-6.43	-6.47	-6.15	-6.19	-6.5	-6.14	-6.41	-6.28	-6.61	-6.24	-6.37	-6.13
LUMO	-1.28	-1.12	-0.08	-1.07	-0.98	-0.17	-1.34	-0.13	-1	-0.06	-0.21	-0.55
Dipole moment	3.46	4.82	0.9	6.24	5.65	0.72	2.91	1.08	5.15	2.16	1.69	4.55
Area	695.33	699.82	649.85	695.26	701.7	652.19	692.9	651.56	577.97	824.67	823.23	824.15
PSA	53.394	54.124	29.423	49.699	52.643	28.987	52.269	29.144	44.29	70.782	68.484	68.159
Volume	663.26	663.21	626.04	663.61	663.56	626.05	662.94	626.37	550.8	774.81	774.61	795.81
Ovality	1.89	1.9	1.84	1.89	1.91	1.84	1.88	1.84	1.78	2.02	2.02	1.98
Min ElPot	-171.72	-189.38	-154.13	-199.92	-198.19	-147.88	-168.79	-164.16	-205.41	-194.16	-201.83	-214
Max ElPot	171.16	124.52	97.66	114.32	108.81	87.56	168.3	74.78	106.37	75.56	88.66	105.8
Polarizability	93.96	93.91	90.73	94	93.9	87.56	93.95	90.73	84.73	102.77	102.76	104.61
Electrostatic charge	0.387	0.287	0.239	0.015	0.157	0.071	0.307	-0.124	0.165	0.244	0.22	0.241
C1 Mulliken charge	0.295	0.349	-0.07	0.305	0.35	-0.051	0.304	-0.05	0.31	0.301	0.299	0.338
C1 Natural charge	0.387	0.395	-0.088	0.385	0.394	-0.068	0.385	-0.07	0.403	0.402	0.401	0.406
C1 exposed Area	5.583	5.515	4.456	6.108	5.484	5.681	5.5	5.018	6.46	7.42	7.296	7.41

Descriptors for donor

C1 Shift	^{13}Carbon NMR chemical shift (ppm) of C1 position
Binary	the orientation of the C2 and C4 substituents on the pyran ring (0 - equatorial, 1- axial)
X1O2	dihedral angle (°) of X1-C1-C2-O2
O2O3	dihedral angle (°) of O2-C2-C3-O3

O3O4	dihedral angle (°) of O3-C3-C4-O4
O4C6	dihedral angle (°) of O4-C4-C5-C6
H1-H2 j	j coupling constant (Hz) of H1-H2
H2-H3 j	j coupling constant (Hz) of H2-H3
H3-H4 j	j coupling constant (Hz) of H3-H4
H4-H5 j	j coupling constant (Hz) of H4-H5
HOMO	highest occupied molecular orbital (eV)
LUMO	lowest unoccupied molecular orbital (eV)
Dipole moment	(debye)
Area	Total surface area ($Å^2$) in a space-filling model
PSA	Polar surface area ($Å^2$) in a space-filling model
Volume	Total volume ($Å^3$) in a space-filling model
Ovality	Measure of deviation from a spherical shape, where 1.0 = a sphere and values > 1.0 indicate deviation
Min ElPot	minimum value of the electrostatic potential(kJ/mol)
Max ElPot	maximum value of the electrostatic potential(kJ/mol)
Polarizability	
C1 Electrostatic charge	Electrostatic charge of C1 position
C1 Mulliken charge	Mulliken charge of C1 position
C1 Natural charge	Natural charge of C1 position
C1 exposed Area	Carbon exposed area ($Å^2$) of C1 position in a space-filling model

Acceptor	MeOH	EtOH	iPrOH	tBuOH	2F-EtOH	3F-EtOH	GlcOH	ManOH
O chem shift	322.409	292.11	253.13	228.836	324.373	314.757	304.638	306.933
O exposed area	12.136	11.811	11.64	11.327	11.999	11.907	11.797	11.134
aC exposed area	24.304	14.908	7.066	1.196	14.469	14.32	13.355	13.276
O electrostatic charge	-0.581	-0.609	-0.685	-0.743	-0.563	-0.557	-0.65	-0.594
O mulliken charge	-0.561	-0.567	-0.562	-0.561	-0.542	-0.535	-0.573	-0.575
O natural charge	-0.709	-0.729	-0.739	-0.749	-0.705	-0.701	-0.725	-0.734
(O-H) IR Peak	3794	3780	3773	3760	3768	3773	3801	3804
(O-H) Lowdin Bond Order	0.913	0.909	0.907	0.906	0.898	0.899	0.91	0.9
(O-H) Mulliken Bond Order	0.814	0.812	0.812	0.811	0.794	0.795	0.81	0.8
aC electrostatic charge	-0.154	0.245	0.708	1.124	-0.04	-0.12	-0.136	-0.108
aC mulliken charge	-0.397	-0.172	0.007	0.133	-0.236	-0.305	-0.161	-0.191
aC natural charge	-0.177	0.003	0.162	0.321	-0.073	-0.097	-0.013	-0.014
aC chem shift	53.816	63.719	70.437	74.443	67.536	66.664	68.979	66.669

Descriptors for Acceptor

O chem shift	^{17}Oxygen NMR chemical shift (ppm) of hydroxyl group
O exposed area	Oxygen exposed area (Å^2) of hydroxyl group in a space-filling model
aC exposed area	Carbon exposed area (Å^2) of αCarbon in a space-filling model
O electrostatic charge	electrostatic charge for Oxygen of hydroxyl group
O mulliken charge	Mulliken charge for Oxygen of hydroxyl group
O natural charge	Natural charge for Oxygen of hydroxyl group
(O-H) IR Peak	IR peak (cm^{-1}) for (O-H) of hydroxyl group
(O-H) Lowdin Bond Order	Lowdin bond order for (O-H) of hydroxyl group
(O-H) Mulliken Bond Order	Mulliken bond order for (O-H) of hydroxyl group
aC electrostatic charge	Electrostatic charge of αCarbon
aC mulliken charge	Mulliken charge of αCarbon
aC natural charge	Natural charge of αCarbon
aC chem shift	^{13}Carbon NMR chemical shift (ppm) of αCarbon

**Activator**	TfOH	Tf2NH	FSO3H	MsOH	C3F6S2O4NH	TMSOTf
HOMO	-2.48	-4.06	-2.36	-1.54	-4.07	-2.48
LUMO	6.93	4.21	6.2	5.3	3.26	6.93
Area	115.34	203.13	82.6	97.58	194.81	115.34
PSA	53.204	80.622	54.752	53.655	81.792	53.204
Volume	85.33	157.35	56.12	70.95	162.28	85.33
Ovality	1.23	1.44	1.17	1.18	1.35	1.23
Min ElPot	-551.43	-518.95	-553.27	-612.23	-523.66	-551.43
Max ElPot	-287.8	-202.17	-398.22	-262.6	-240.25	-287.8
Polarizability	46.08	52.19	43.91	45.51	52.81	46.08
Dipole Moment	4.46	0.55	0.59	3.99	5.7	4.46
O-/N- Electrostatic charge	-0.667	-0.778	-0.685	-0.748	-0.791	-0.667
O-/N- Mulliken charge	-0.576	-0.706	-0.579	-0.616	-0.67	-0.576
O-/N- Natural charge	-0.979	-1.204	-0.96	-1.018	-1.182	-0.979
O/N NMR	102.741	-222.564	88.328	85.395	-213.443	102.741
Si NMR	151.164	172.984	110.241	153.289	176.308	151.164
O-/N-area	17.7	9.7	18.3	17.9	10.6	17.7

Descriptors for Activator (Conjugate Base)

HOMO	highest occupied molecular orbital (eV)
LUMO	lowest unoccupied molecular orbital (eV)
Area	Total surface area (Å^2) in a space-filling model
PSA	Polar surface area (Å^2) in a space-filling model
Volume	Total volume (Å^3) in a space-filling model
Ovality	Measure of deviation from a spherical shape, where 1.0 = a sphere and values > 1.0 indicate deviation
Min ElPot	minimum value of the electrostatic potential(kJ/mol)
Max ElPot	maximum value of the electrostatic potential(kJ/mol)
Polarizability	
Dipole Moment	(debye)
O-/N- Electrostatic charge	Electrostatic charge of oxygen (O-) or nitrogen anion (N-)
O-/N- Mulliken charge	Mulliken charge of oxygen (O-) or nitrogen anion (N-)

O-/N- Natural charge	Natural charge of oxygen (O-) or nitrogen anion (N-)
O-/N- NMR	Oxygen (O-) or nitrogen anion (N-) NMR chemical shift (ppm)
Si NMR	Silicon NMR chemical shift (ppm)
O-/N-area	Exposed area ($Å^2$) of oxygen (O-) or nitrogen anion (N-) in a space-filling model

Solvent	DCM	Toluene	ACN	MTBE	3F-Toluene	Chloroform	tBu-Benzene	1,4-Dioxane	Anisole
Dipole Moment	1.87	0.32	3.86	1.23	2.88	1.25	0.27	0	1.36
HOMO	-8.65	-6.64	-9.14	-6.89	-7.52	-8.81	-6.65	-6.61	-6.09
LUMO	-0.73	-0.18	0.72	1.01	-1.07	-1.57	-0.2	1.34	-0.21
Area	84.21	134.86	73.41	139.63	150.61	100.4	187.27	113.55	144.68
PSA	0	0	15.278	6.748	0	0	0	15.79	6.908
Volume	61.24	117.38	53.06	115.42	131.17	75.22	170.86	94.03	126.19
Ovality	1.12	1.16	1.08	1.22	1.21	1.17	1.26	1.14	1.19
Log P	1.01	2.52	0.67	0.96	2.96	1.67	3.74	-0.31	1.91
Polarizability	44.47	49.32	43.35	48.87	50.49	45.76	53.71	47.13	50.22
Min ElPot	-62.13	-95.18	-191.22	-170.72	-79.32	-37.44	-94.82	-156.08	-126.36
Max ElPot	146.58	69.25	136.70	59.82	105.79	170.92	74.38	75.70	92.35

Descriptors for Solvent

Dipole Moment	(debye)
HOMO	highest occupied molecular orbital (eV)
LUMO	lowest unoccupied molecular orbital (eV)
Area	Total surface area (Å^2) in a space-filling model
PSA	Polar surface area (Å^2) in a space-filling model
Volume	Total volume (Å^3) in a space-filling model
Ovality	Measure of deviation from a spherical shape, where 1.0 = a sphere and values > 1.0 indicate deviation
Log P	octanol water partition coefficient
Polarizability	
Min ElPot	minimum value of the electrostatic potential(kJ/mol)
Max ElPot	maximum value of the electrostatic potential(kJ/mol)
